# Synthesis and antidiabetic evaluation of novel Tetrahydroacridine derivatives with computational insights

**DOI:** 10.1038/s41598-025-28702-2

**Published:** 2025-12-13

**Authors:** Asmaa M. Kadry, Wafaa A. Abdellah, Mounir A. A. Mohamed, Mahmoud S. Tolba

**Affiliations:** 1 Chemistry Department, Faculty of Science, Sohag University, Sohag, 82524 Egypt; 2 Medical Pharmacology Department, Faculty of Medicine, Sohag University, Sohag, Egypt; 3 Chemistry Department, Faculty of Science, New Valley University, El-Kharja, 72511 Egypt

**Keywords:** Tetrahydroacridine, THAs, Synthesis, Pfitzinger reaction, Antidiabetic, Biochemistry, Chemical biology, Chemistry, Computational biology and bioinformatics, Drug discovery

## Abstract

A new series of thirty-two tetrahydroacridine (THA) derivatives were rationally designed and synthesized via efficient multistep reactions involving nucleophilic aromatic substitution and Mannich condensation. The structural integrity and purity of the obtained compounds were confirmed by their spectroscopic techniques including ^1^H-NMR, ^13^C-NMR, mass spectroscopy and elemental analyses. The synthesized tetrahydroacridine (THA) derivatives were initially screened for antidiabetic potential through a glucose diffusion assay, where compound **4b** (100 mg/kg) exhibited the highest inhibition rate (34.00%) at the lowest concentration tested, outperforming the reference drug Gliclazide. To deepen biological understanding, molecular docking studies were conducted against key diabetic targets: DPP-IV, SGLT1, and GLUT2. The docking results demonstrated that several compounds, particularly compound **4a**, compound **5a**, and compound **4c**, achieved excellent binding affinities and interactions with critical active-site residues, showing potential as dual or triple inhibitors. ADMET and drug-likeness evaluations based on Lipinski’s rule of five and computational pharmacokinetic modeling indicated acceptable oral bioavailability, metabolic stability, and low toxicity for selected lead candidates. *In-vivo* studies were conducted in streptozotocin-induced diabetic Wistar rats over a 4-week period. Compounds **5b**, **7a**, and **8b** (100 mg/kg) significantly decreased blood glucose level during fasting, approaching or surpassing the efficacy of Gliclazide. Compound **5b** showed the most pronounced effect, lowering glucose to 180.5 ± 12.3 mg/dL by week 4. These integrated findings highlight the potential of tetrahydroacridine derivatives as promising scaffolds for the development of new antidiabetic candidates with multitarget activity, favorable drug-like properties and minimal toxicity risk. Further in-depth SAR, enzyme inhibition, and pharmacodynamic studies are warranted to advance these candidates toward clinical relevance.

**Supplementary Information:**

The online version contains supplementary material available at 10.1038/s41598-025-28702-2.

## Introduction

Acridine and tetrahydroacridine (THA) derivatives are well-established DNA intercalators, with extensive research conducted on their synthesis^1,2^, physicochemical properties^3,4^, structural characteristics^5^, and biological effects^6,7^. Due to their planar structure, these compounds intercalate into DNA and RNA by forming hydrogen bonds and stacking between base pairs, leading to DNA cross-links and strand breaks^8^. Numerous natural and synthetic THA derivatives have been evaluated for their antimalarial^9^, anti-inflammatory^10,11^, and analgesic^12^ properties, with some approved for chemotherapeutic use.

In medicinal chemistry, THA derivatives hold significant importance due to their diverse biological activities, including antitumor effects^13^, DNA-binding and photo-damaging capabilities^14^, antileishmanial^15^, and antimicrobial actions^16–18^. Additionally, they exhibit acetylcholinesterase inhibitory properties^19–21^, making them valuable as memory-enhancing agents for Alzheimer’s disease treatment^22,23^. Acetylcholine, which constricts bronchi, enhancing salivation and digestion, and regulating intestinal motility, along with influencing blood glucose levels^24,25^. These observations motivated us to synthesize novel acridine derivatives and assess their antidiabetic potential^26^.

Diabetes mellitus (DM) is a widespread chronic condition marked by hyperglycemia^27,28^. According to the IDF (Diabetes Atlas 11th Edition (2025), about 11.1% of adults aged 20–79 years equivalent to 589 million people worldwide live with diabetes. Similarly, WHO estimates show that in 2022, about 14% of adults aged 18 years and over have diabetes, compared to about 7% in 1990, underscoring a significant global increase^29,30^. DM is primarily categorized into type 1 (DM) and type 2 (DMII)^31,32^, with over 90% of cases being DMII (non-insulin-dependent diabetes mellitus; NIDDM). This form is characterized by insulin resistance in tissues and impaired insulin secretion due to beta-cell dysfunction, necessitating insulin therapy for disease management^33^. DM contributes to substantial morbidity and mortality through microvascular complications (retinopathy, neuropathy, nephropathy) and macrovascular events (heart attack, stroke, peripheral vascular disease). These updated data highlight the growing burden of diabetes and provide the current epidemiological context for exploring novel therapeutic agents.

**Fig. 1:**
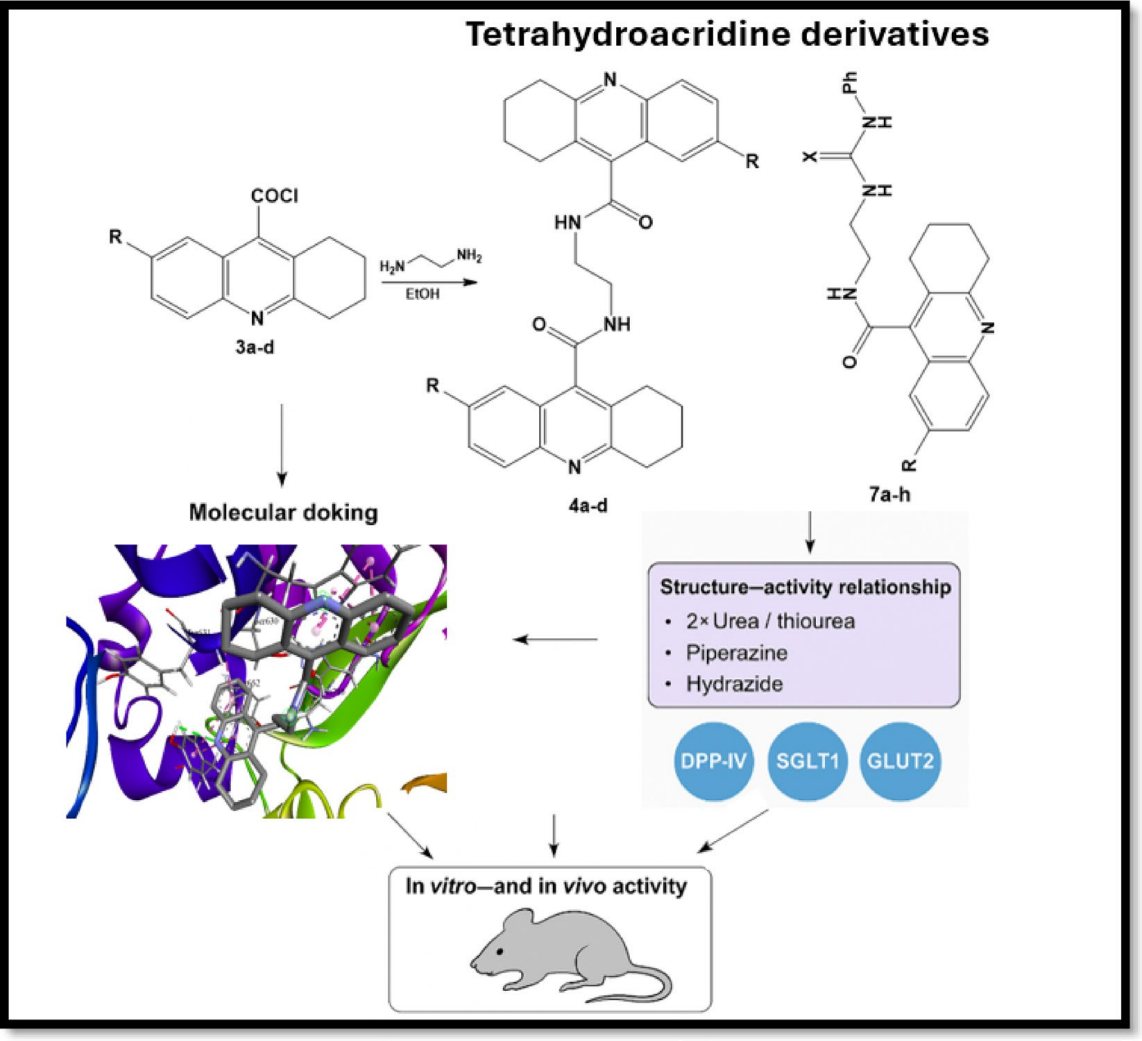
Proposed synthetic route for novel tetrahydroacridine derivatives.

The rationale for evaluating THA derivatives as potential antidiadetic agents stems from two major cosiderations. First, THA scaffolds are well-known acetylcholinesterase inhibitors, and acetylcholine plays a regulatory role in glucose homeostasis through modulation of insulin secretion and intestinal motility. This biochemical link suggested that modifying THA structures may influence blood glucose regulation. Second, despite the extensive investigation of THA derivatives in neurodegenerative and antimicrobial fields, their potential in metabolic disorders, particularly diabetes mellitus, remains largely unexplored. Therefore, in this study we designed, synthesized, and evaluated novel THA derivatives with the hypothesis that tailored structural modifications could yield compounds with dual or multitarget inhibitory activity (α-glucosidase, α-amylase, DPP-IV, SGLT1, and GLUT2), ultimately offering new scaffolds for antidiabetic drug discovery (Fig. 1). To our knowledge, this study represents the first comprehensive evaluation combining biological and computational analyses of these derivatives for antidiabetic applications.

## Results and discussion

**Scheme 1:**
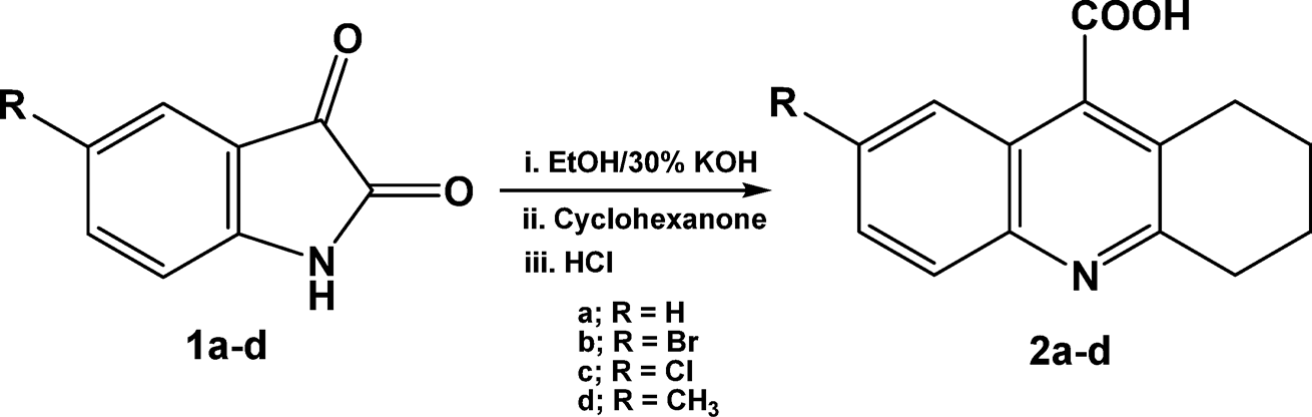
Synthesis of 1,2,3,4-Tetrahydroacridine-9-carboxylic acid derivatives **2a–d**.

Building upon our previous studies in synthetic organic chemistry, this work extends our exploration of biologically active heterocyclic frameworks by designing and synthesizing new tetrahydroacridine-based compounds with potential pharmacological relevance^34–39^**.** We applied Pfitzinger reaction of isatin derivatives **1a–d** with cyclohexanone as described in literature^40^. 1,2,3,4-Tetrahydroacridine-9-carboxylic acid derivatives **2a–d** obtained in good to excellent yield (78–88%), Scheme 1.

**Scheme 2:**
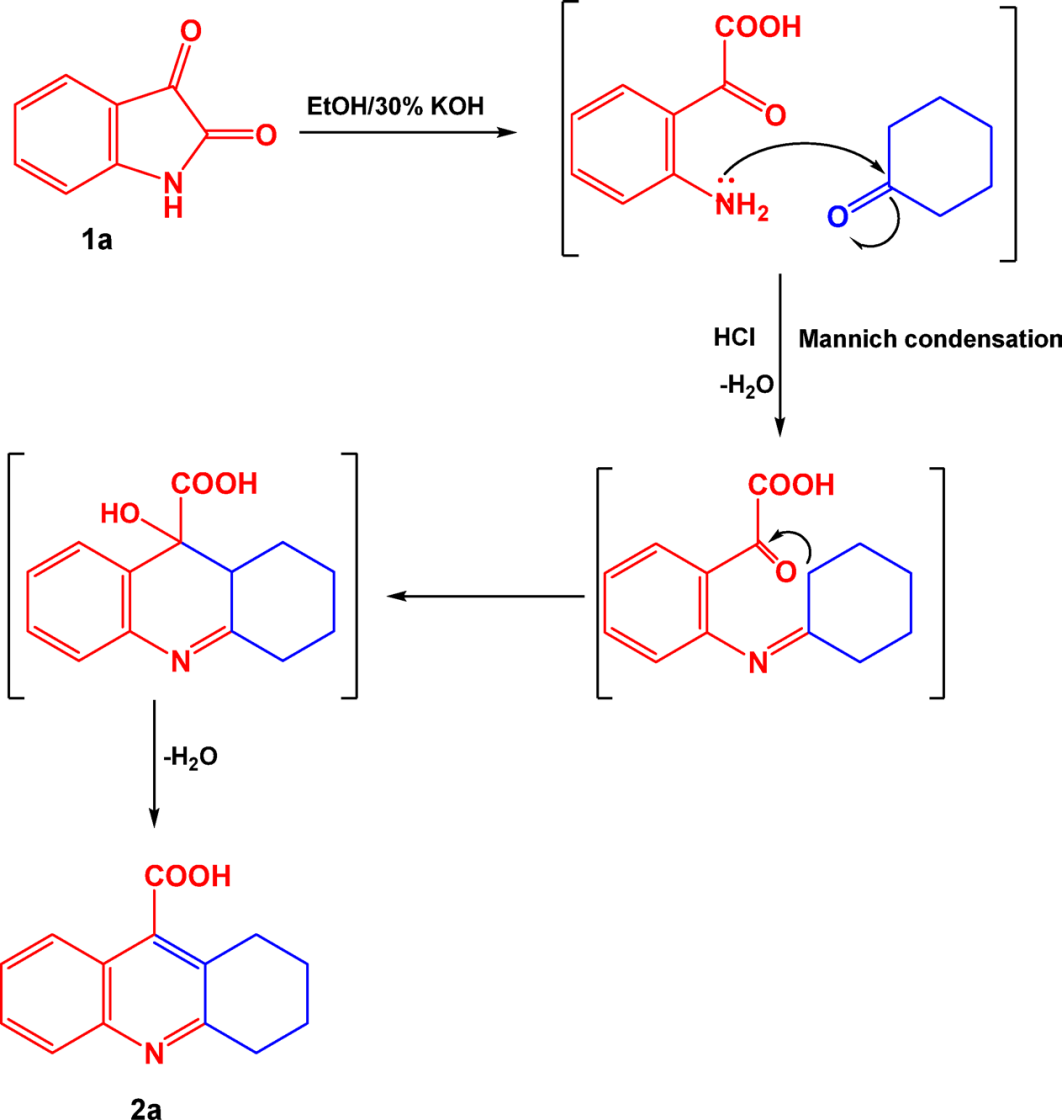
The expected reaction mechanism of preparation **compound 2a**.

The reaction mechanism of this reaction was assumed to proceed via a preliminary hydrolysis of isatin resulting in formation of isatoic acid which in turn undergoes in situ Mannich condensation with cyclohexanone forming compound 2a, Scheme 2.

Here, carbonyl group absorption band at 1642 cm^−1^, compound** 2a’s** infrared spectra had a distinctive absorption wide band at 3467–3318 cm^−1^ that corresponded to the COOH group. The ^1^H-NMR spectrum of compound **2a** revealed a set of triplet and multiplet bands between δ 1.22–1.87 ppm corresponding to the 4 cyclic CH_2_ protons and a characteristic signal (D_2_O exchangeable) at δ 12.24 ppm assigned to carboxyl group proton. Also mass spectrum of it showed molecular ion peak at m/z 227 [M^+1^].

**Scheme 3:**
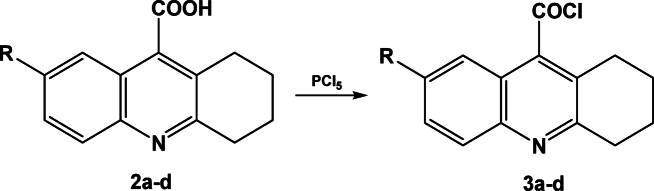
Synthesis of 1,2,3,4-Tetrahydroacridine-9-acid chloride derivatives **3a–d**.

1,2,3,4-Tetrahydroacridine-9-carboxylic acid derivatives **2a–d** were then converted to their corresponding acid chlorides **3a–d** using phosphorus pentachloride (PCl_5_) under dry conditions, Scheme 3. The IR spectra of compounds **3a–d** showed absorption bands at 690–650 cm^−1^ for C–Cl and another bands at 1780–1740 cm^−1^ for carbonyl groups.

**Scheme 4:**
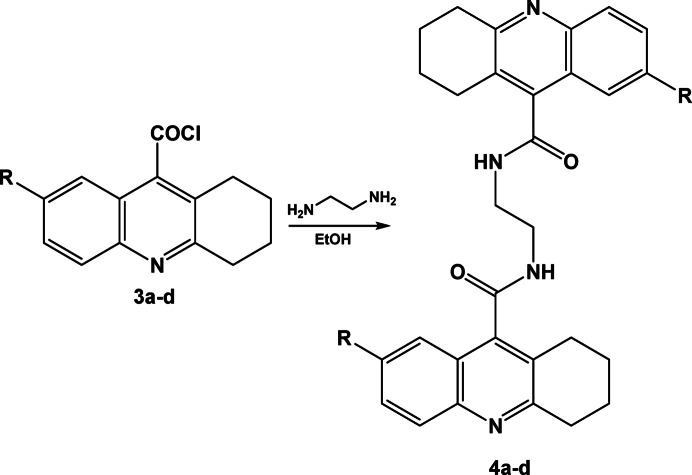
Synthesis of N,N’-(ethane-1,2-diyl)bis(1,2,3,4-tetrahydroacridine-9-carboxamide) derivatives **4a–d**.

Compounds **3a–d** were reacted with ethylenediamine in ethanol in different molar ratios, where the corresponding *N,N’*-(ethane-1,2-diyl)bis(1,2,3,4-tetrahydroacridine-9-carboxamide) derivatives **4a–d** were obtained, Scheme 4. First, the reaction was achieved using equimolar ratio of compound **3a–d** and ethylenediamine where compounds **4a–d** were obtained in poor yield (25–35%), but using two folds of acid chlorides **3a–d** increases the reaction yield to 60–70%. The IR spectrum of compounds **4a** was characterized with the appearance of moderate absorption bands ranged between 3285 and 3180 cm^−1^ for NH group, 1666 for C=O group. The the ^1^H-NMR spectrum of compound **4a** revealed a characteristic singlet at 3.22 ppm for ethylenediamine protons.

**Scheme 5:**
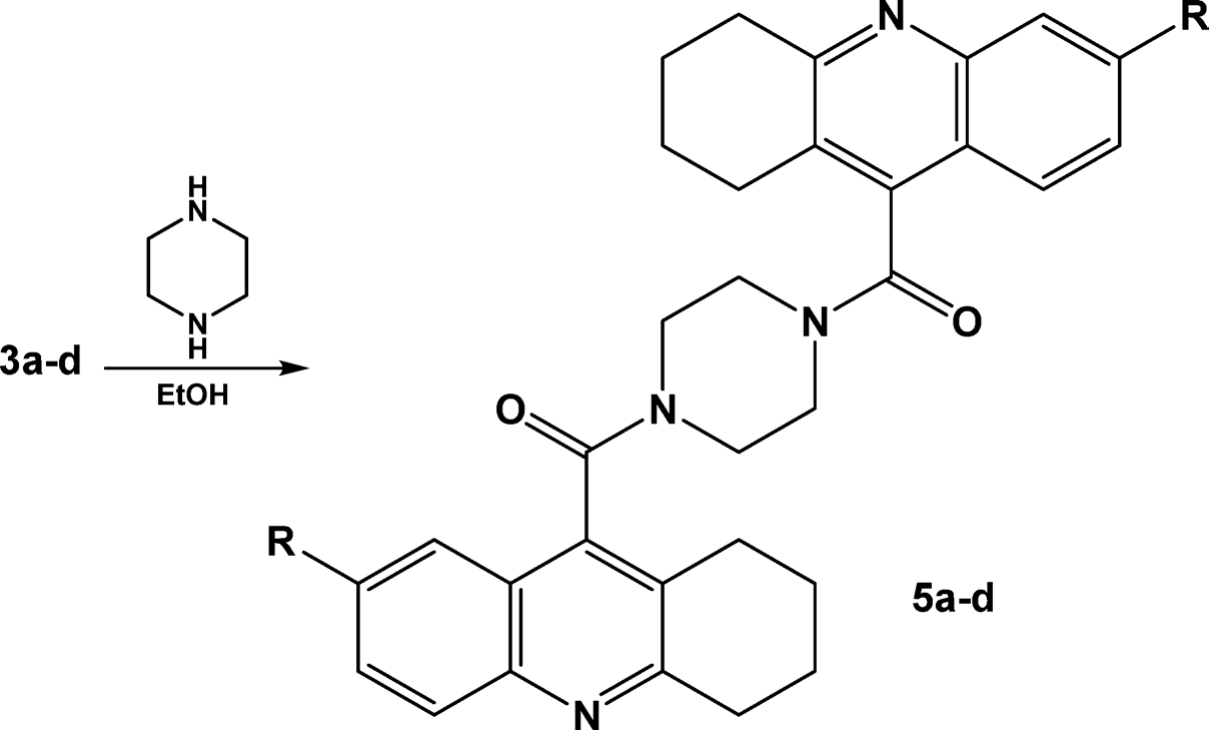
Synthesis of piperazine-1,4-diylbis((1,2,3,4-tetrahydroacridin-9-yl)methanone) derivative **5a–d**.

Similarly, compounds **3a–d** were allowed to react with piprazine in aqueous ethanol, after working up, piperazine-1,4-diylbis((1,2,3,4-tetrahydroacridin-9-yl)methanone) derivative **5a–d** were obtained, Scheme 5. The IR spectra of compounds **5a–d** were characterized with the appearance of moderate absorption bands corresponding to carbonyl groups at 1678, 1676, 1672 and 1666 cm^−1^ respectively. Where the ^1^H-NMR spectra were characterized with singlet signals at 3.34, 3.35, 3.36 and 3.35 ppm for piperazine symmetric protons.

**Scheme 6:**
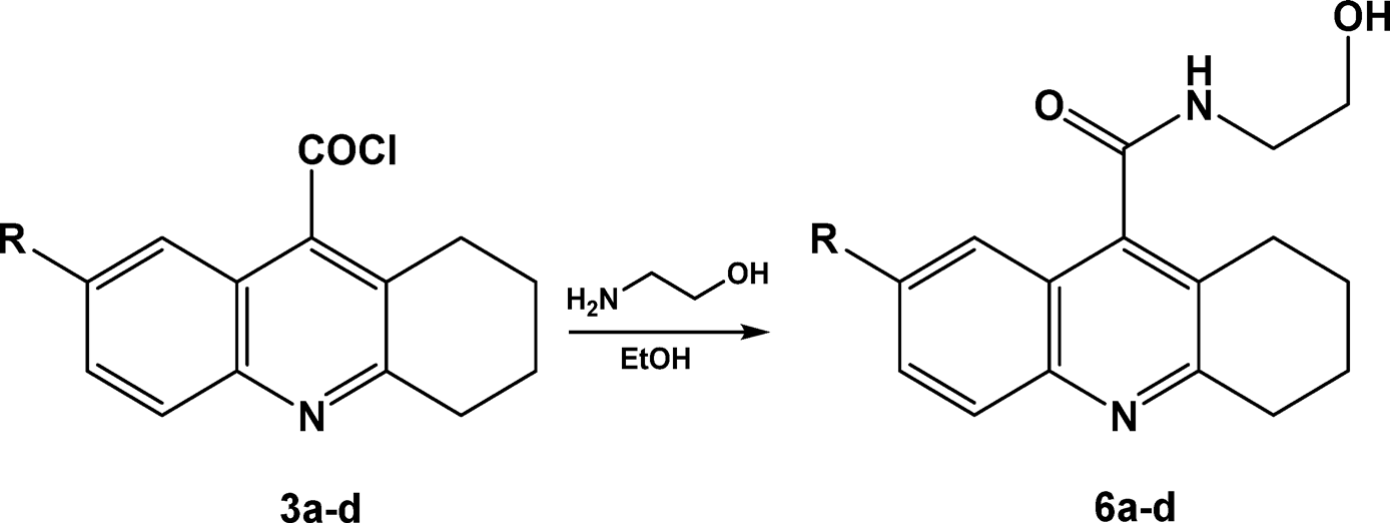
Synthesis of N-(2-hydroxyethyl)-1,2,3,4-tetrahydroacridine-9-carboxamide derivatives **6a–d**.

Also, treatment of compounds **3a–d** with ethanolamine in ethanol at room temperature afforded the corresponding N-(2-hydroxyethyl)-1,2,3,4-tetrahydroacridine-9-carboxamide derivatives **6a–d**, Scheme 6. The IR spectrum of compounds **6a** were revealed bands at 3411 and 3252 cm^−1^ characterized for OH and NH groups, where the ^1^H-NMR spectrum of compound **6a** showed exchangeable bands and 6.11 and 11.02 ppm for OH and NH goups respectively.

**Scheme 7:**
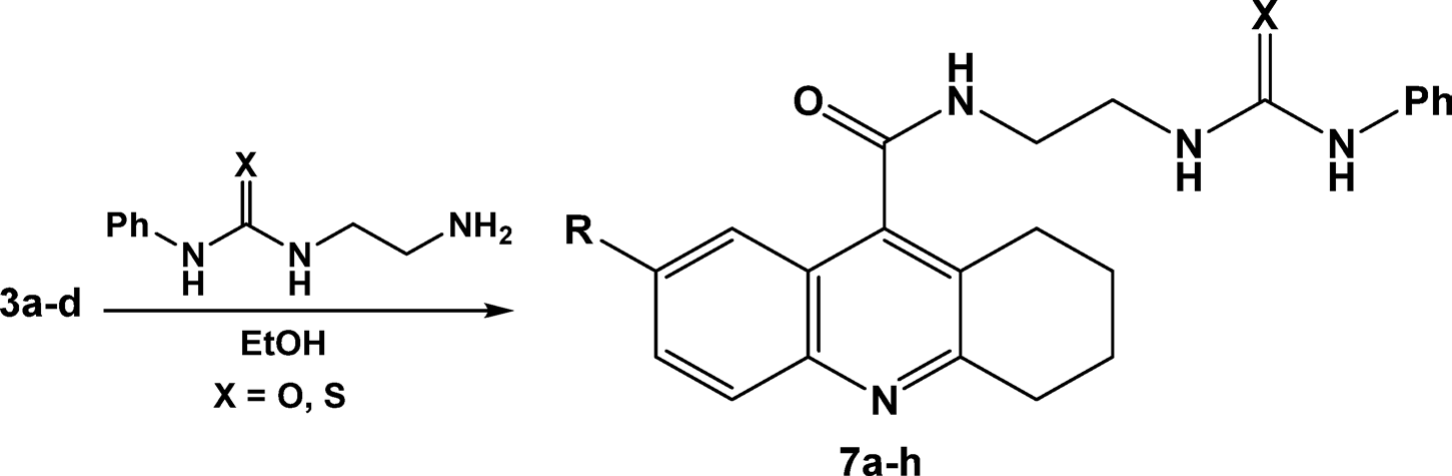
Synthesis of N-(2-(3-phenylureido)ethyl)-1,2,3,4-tetrahydroacridine-9-carboxamide derivatives **7a–h**.

Reaction of compounds **3a–d** with 1-(2-aminoethyl)-3-phenylurea or (1-(2-aminoethyl)-3-phenylthiourea) where the corresponding *N*-(2-(3-phenylureido)ethyl)-1,2,3,4-tetrahydroacridine-9-carboxamide derivatives **7a–h** were formed, Scheme 7. Three NH groups were appeared in the in the IR spectrum of compound **7b** at 3278, 3212 and 3185 cm^−1^, where the ^1^H-NMR spectrum revealed an increase in the aliphatic and aromatic protons as well as the appearance of 3 NH groups at 8.67, 9.22 and 11.34 ppm.

**Scheme 8:**
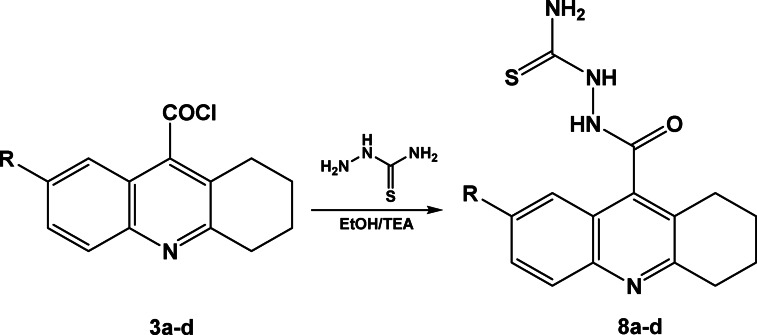
2-(1,2,3,4-tetrahydroacridine-9-carbonyl)hydrazine carbothioamide derivatives **8a–d**.

Additionally, compounds **3a–d** were permitted to react with thiosemicarbazide in ethanol and with a catalytic quantity of triethylamine. This resulted in the excellent yield of the corresponding 2-(1,2,3,4-tetrahydroacridine-9-carbonyl)hydrazine carbothioamide derivatives **8a–d**, Scheme 8. The IR spectrum of compound **8c** showed absorption peaks at 3318, 3268, 3215 and 3170 cm^−1^ for NH_2_ and 2NH groups, where the ^1^H-NMR spectrum of of compound **8c** revealed exchangeable bands at 6.62 ppm (broad band) for NH_2_ group, 9.22 and 11.23 ppm characteristic for 2 NH groups.

## Biological screening

### *In-vitro* antidiabetic evaluation of tetrahydroacridine derivatives (inhibition of α-amylase enzyme)

Starch solution (0.1%) was prepared by dissolving 0.1 g of starch in 100 mL of sodium acetate buffer (pH = 4.8, 16 mM). An enzyme solution was prepared by dissolving 27.5 mg of α-amylase in 100 mL of deionized H_2_O. A colorimetric reagent was prepared by dissolving 1 g of 3,5-dinitro salicylic acid in deionized H_2_O (20 mL) and 0.16 g sodium hydroxide (in 10 mL deionized H_2_O) and 4 g of sodium potassium tartrate was added gradually to the mixture. The mixture was mixed well and the volume was made up to 100 mL using deionized H_2_O. Both control (100 μL) and the THA derivatives (100 μL) were separately mixed with the starch solution (100 μL) and left for 30 min to react with the α-amylase solution (under alkaline conditions at 25 ºC). The action was recorded after 5 min. The liberated maltose was measured quantitatively by the reduction of 3,5-dinitro salicylic acid to 3-amino-5-nitrosalicylic acid. This reaction was measured at 540 nm^41–44^. 

A series of newly synthesized Tetrahydroacridine (THA) derivatives were evaluated for their antidiabetic potential using an *in-vitro* glucose diffusion assay. The experiment measured absorbance values of glucose at different molar concentrations (6.25–100 mg/dL) in the presence of different THA compounds, and the results were benchmarked against the standard antidiabetic drug Gliclazide (100 mg/kg)^45,46^, results of *in-vitro* antidiabetic evaluation of the newly synthesized THA derivatives were listed in Table 1.

**Fig. 2:**
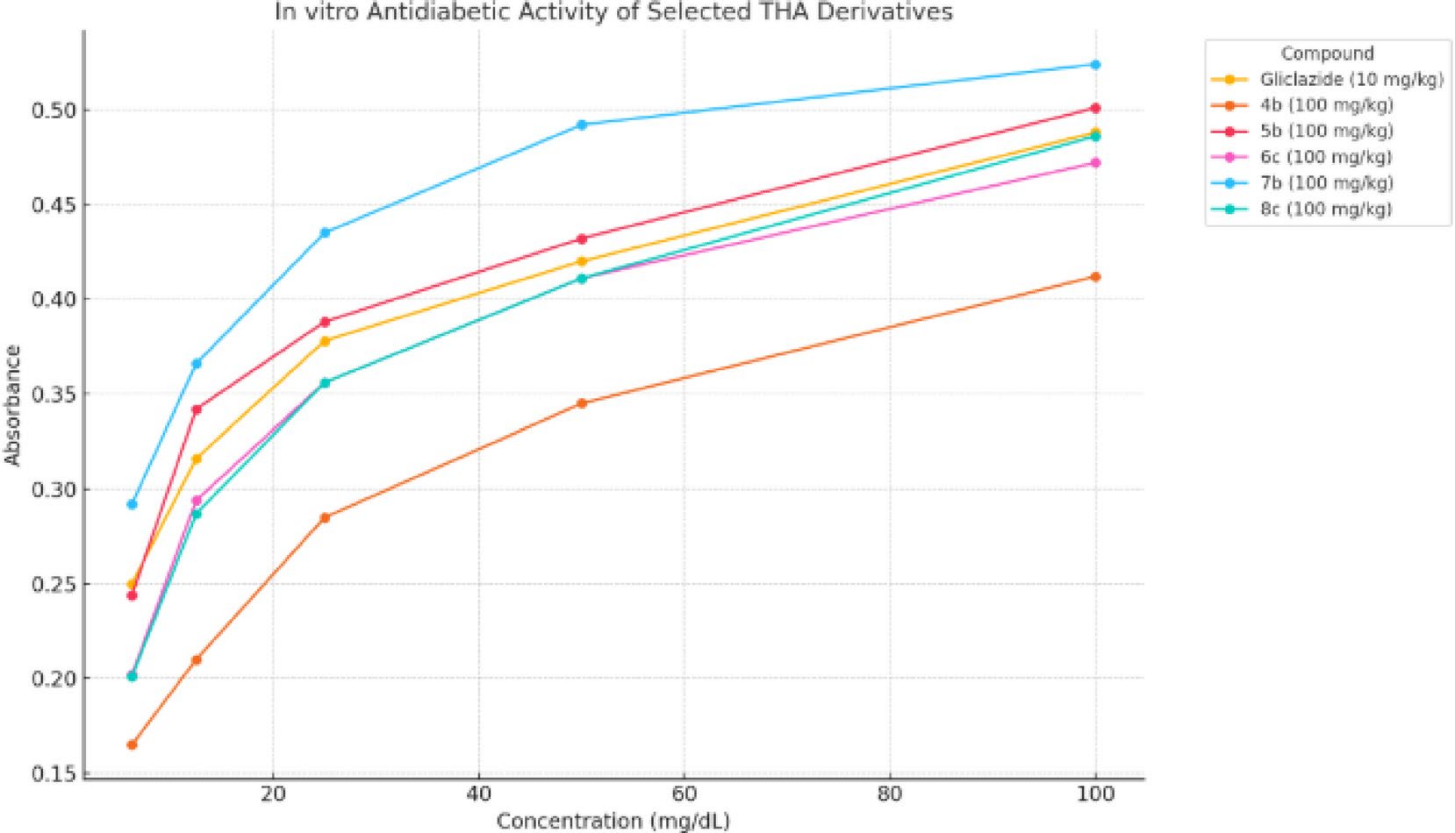
(**a**) Absorbance of glucose in the presence of gliclazide. (**b**) *In-vitro* antidiabetic activity of selected THA derivatives.

**Table 1:**
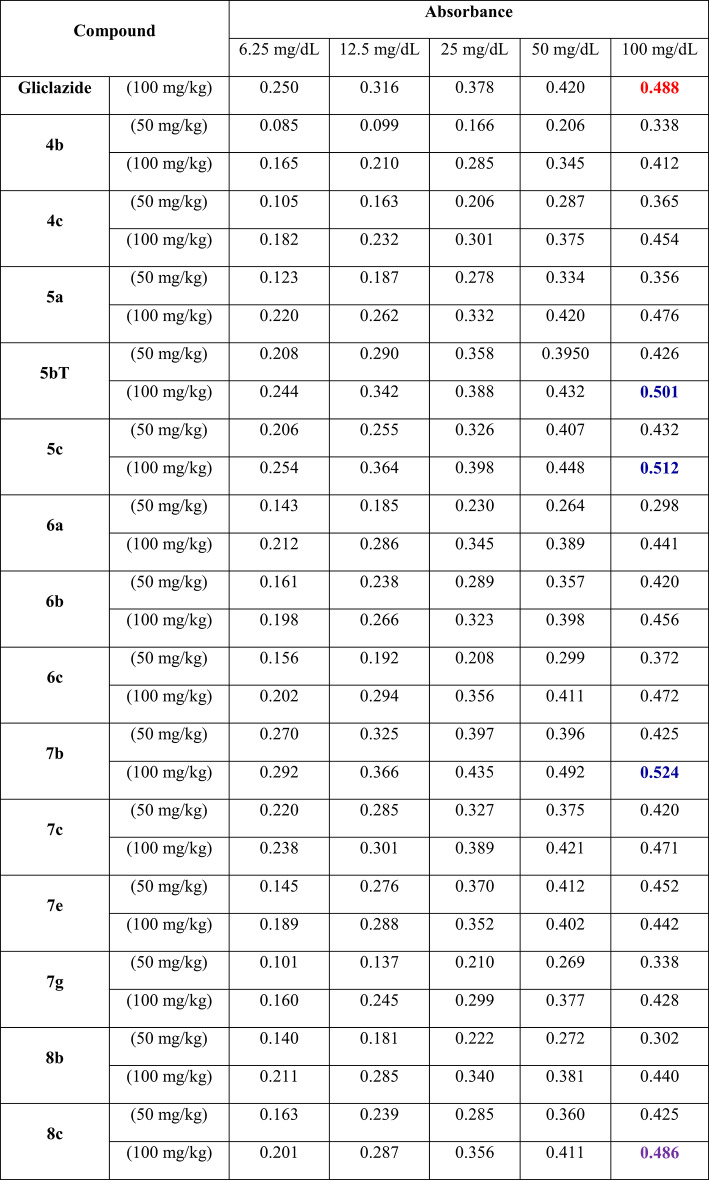
Effect of some tetrahydroacridine derivatives (THAs) on glucose diffusion compared to Gliclazide.

As shown in Fig. 2a, Gliclazide exhibited the highest absorbance values across all concentrations, indicating less restriction of glucose diffusion compared to many of the tested THA derivatives. Figure 2b, illustrates the antidiabetic activity of selected Tetrahydroacridine (THA) derivatives based on their *in-vitro* performance, assessed through absorbance measurements at various glucose concentrations. The results are compared to the reference drug, Gliclazide. Here, the most effective compounds at the 100 mg/kg dose were selected to clearly demonstrate the differences in activity.

Among these, compound **4b** at 100 mg/kg demonstrated the most potent inhibition of glucose diffusion, showing significantly lower absorbance values and achieving a maximum inhibition of 34.00% at 6.25 mg/dL (see Table 2). Other derivatives such as **6c** (100 mg/kg) and **8c** (100 mg/kg) exhibited moderate inhibition, with values ranging from 3 to 20% depending on concentration. Conversely, compounds like **5b** (100 mg/kg) and **7b** (100 mg/kg) showed negative inhibition values at certain concentrations, indicating lower efficacy than Gliclazide and potential promotion of glucose diffusion.

**Table 2:** Percentage inhibition of glucose diffusion compared to gliclazide.

Concentration (mg/dL)	Gliclazide (100 mg/kg)	4b (100 mg/kg)	5b (100 mg/kg)	6c (100 mg/kg)	7b (100 mg/kg)	8c (100 mg/kg)
6.25	0.00	34.00	2.40	19.20	− 16.80	19.60
12.50	0.00	33.54	− 8.23	6.96	− 15.82	9.18
25.00	0.00	24.60	− 2.65	5.82	− 15.08	5.82
50.00	0.00	17.86	− 2.86	2.14	− 17.14	2.14
100.00	0.00	15.57	− 2.66	3.28	− 7.38	0.41

**Fig. 3:**
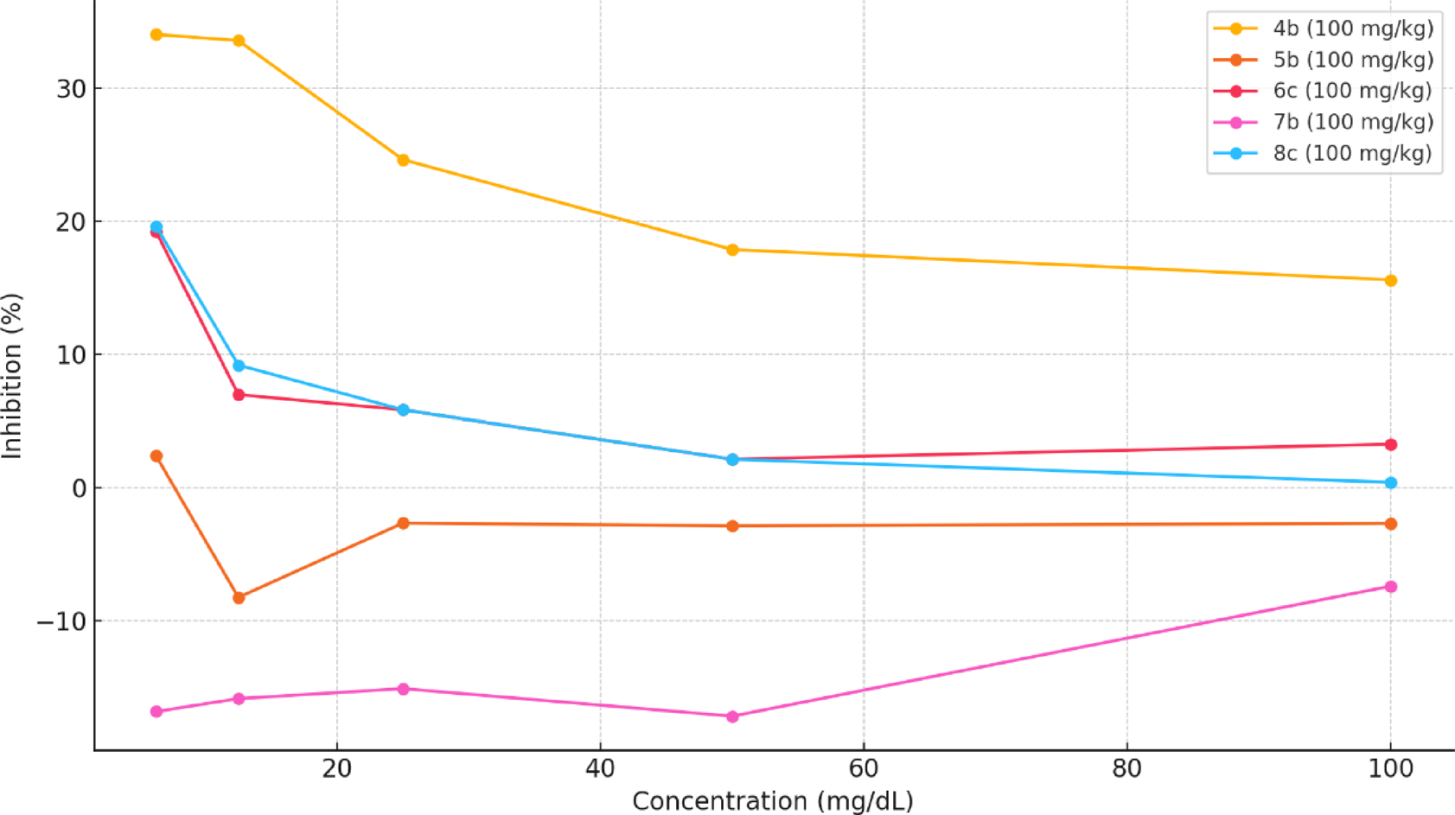
Percentage inhibition of glucose diffusion compared to gliclazide.

The percentage inhibition results, graphically represented in Fig. 3, highlights the concentration-dependent behavior of the THA compounds. Compound **4b** consistently outperformed gliclazide at all concentrations. These findings suggest that specific structural modifications in the THA scaffold can enhance antidiabetic activity, warranting further *in-vivo* and mechanistic studies.

### *In-vivo* antidiabetic evaluation of tetrahydroacridine derivatives

#### Animals

Male Wistar albino rats weighing between 200 and 250 grames were used in the experiment. They were raised and kept in the institution’s animal house with unlimited access to water and pelleted food. For around a week, the rats were kept in a controlled setting with a 12-h to allow them to acclimatise. The animals were locally anesthetized with lidocaine (Xylocaine) prior to sample collection to minimize pain and discomfort. The Sohag University Faculty of Science’s animal ethics committee gave its approval to the study’s procedure. The experimental plan was authorized by Sohag University, Faculty of Science Institutional Animal Care and Use Committee (IACUC), Protocol Number: SU-FS-29-25.

#### Dosage calculation

Gliclazide was taken orally in 1% gum acacia as a conventional antidiabetic (100 mg/kg)^47^. All synthetic THA derivatives’ equivalent dosages were determined based on their molecular weight [MWt].

#### Model sucrose-loaded (SLM)

Overnight fasting was done on male Wistar rats. Following the first collection of blood, the chemicals under investigation were administered orally by gavage to matched groups of six rats each. After a half-hour posttest treatment, each rat received a sucrose load of 2 gm/kg body weight. After the sugar load, blood was drawn 30, 60, 90, and 120 min later^48^. Blood glucose concentration was estimated using a commercial glucometer.

#### Toxicity study

Investigation of toxicity. The THA derivatives that shown antihyperglycemic action in this investigation were tested for their impact on serum liver and kidney markers as part of an *in-vivo* acute toxicity evaluation.

#### Experimental diabetes induction

The animals were locally anesthetized with lidocaine (Xylocaine) prior to sample collection to minimize pain and discomfort.

A single intraperitoneal injection of streptozotocin (STZ( at a concentration of 65 mg/kg was used to induce diabetes in rats that had been fasted overnight. Before injection, STZ was freshly dissolved in ice-cold citrate buffer (0.01 M, pH 4.5)^49^. Rats with blood glucose levels > 200 mg/dl after 48 h were included to the experiment^50^.

#### Design of an experiment

Twelve tetrahydroacridine derivatives were tested for their antihyperglycemic effects in seventy-six rats (fourteen groups of five to six rats each). Group 2 was diabetic plus Gliclazide (100 mg/kg), a standard antidiabetic medication; Group 1 was diabetic control. The different tetrahydroacridine derivatives^51,52^ were distributed to groups 3–14. Gliclazide and several THA compounds were given orally to the treatment groups.

#### Methodology

For each group, blood glucose was determined at zero, one, two, four, and six hours following oral administration of derivatives using glucometer (Faculty of Science, Sohag University).

#### Tetrahydroacridines’ impact on blood glucose levels during fasting

Normal, diabetic control, and diabetic treated rats all had altered blood glucose levels during the course of the 4-week therapy. Table 3. presents the findings. Normal rats’ fasting blood glucose levels remained unchanged until the conclusion of the time, while untreated diabetic rats’ blood glucose levels were noticeably higher than those of normal controls.

**Table 3:** Gliclazide and specific tetrahydroacridine derivatives’ effects on the fasting blood glucose levels of treated and normal diabetic rats.

Group		Blood Glucose Level During Fasting (mg/dl)				
		Week 0	Week 1	Week 2	Week 3	Week 4
Normal control		72.5 ± 5.20	73.8 ± 4.80	72.7 ± 6050	73.1 ± 5.50	75.6 ± 3.80
Diabetic control		322.4 ± 16.80	408.5 ± 20.50	422.3 ± 38.60	432.6 ± 32.50	440.5 ± 35.50
Gliclazide (100 m mg/kg)		335.2 ± 15.00	27,203 ± 20.6	230.2 ± 18.7	216.5 ± 10.8	201.2 ± 12.50
4b	(50 mg/kg)	321.5 ± 15.0	302.2 ± 12.5	288.1 ± 12.1	262.8 ± 13.6	243.5 ± 12.5
	(100 mg/kg)	324.5 ± 16.3	292.2 ± 12.3	265.0 ± 15.6	232.5 ± 10.4	213.8 ± 12.0
4c	(50 mg/kg)	324.0 ± 12.5	301.4 ± 14.5	285.1 ± 12.8	256.1 ± 12.1	240.1 ± 13.8
	(100 mg/kg)	330.7 ± 13.8	288.8 ± 13.3	253.5 ± 13.6	219.5 ± 16.5	205.5 ± 10.4
4d	(50 mg/kg)	314.2 ± 14.0	284.1 ± 13.3	245.8 ± 12.2	211.6 ± 12.3	212.5 ± 12.2
	(100 mg/kg)	314.7 ± 14.5	298.7 ± 14.5	272.2 ± 13.5	243.9 ± 14.0	227.0 ± 12.8
5a	(50 mg/kg)	317.5 ± 12.0	301.3 ± 13.0	274.5 ± 13.5	240.5 ± 12.0	222.0 ± 14.0
	(100 mg/kg)	314.7 ± 14.5	284.1 ± 13.3	245.8 ± 12.2	211.6 ± 12.3	212.5 ± 12.2
5b	(50 mg/kg)	313.8 ± 10.2	291.0 ± 13.5	261.8 ± 12.5	239.5 ± 12.0	208.5 ± 12.6
	(100 mg/kg)	310.4 ± 12.5	280.6 ± 13.7	237.3 ± 12.7	200.6 ± 12.5	180.5 ± 12.3
6a	(50 mg/kg)	312.4 ± 10.7	295.7 ± 14.5	253.5 ± 12.8	232.8 ± 14.1	202.1 ± 13.5
	(100 mg/kg)	316.4 ± 12.2	288.6 ± 12.8	244.4 ± 10.5	208.6 ± 12.4	192.5 ± 12.7
6b	(50 mg/kg)	312.4 ± 13.0	280.3 ± 14.8	255.4 ± 12.0	230.6 ± 14.5	210.5 ± 12.5
	(100 mg/kg)	315.4 ± 13.5	268.6 ± 14.1	230.4 ± 12.7	210.6 ± 12.5	186.5 ± 12.8
7a	(50 mg/kg)	325.0 ± 11.2	296.5 ± 13.0	252.0 ± 12.2	232.5 ± 12.5	198.0 ± 10.2
	(100 mg/kg)	328.8 ± 11.8	280.8 ± 13.6	238.5 ± 11.4	201.5 ± 12.3	178.5 ± 10.9
7b	(50 mg/kg)	325.5 ± 14.2	292.5 ± 13.0	255.3 ± 13.1	231.5 ± 12.8	201.5 ± 12.0
	(100 mg/kg)	325.5 ± 14.2	280.8 ± 12.5	245.0 ± 12.6	210.8 ± 12.1	182.4 ± 12.5
7c	(50 mg/kg)	305.2 ± 14.5	285.6 ± 15.0	256.6 ± 14.3	238.2 ± 12.0	210.6 ± 12.5
	(100 mg/kg)	305.2 ± 14.5	270.05 ± 13.6	237.2 ± 14.5	218.5 ± 14.6	188.5 ± 14.0
7f.	(50 mg/kg)	325.5 ± 14.2	292.5 ± 13.0	255.3 ± 13.1	231.5 ± 12.8	201.5 ± 12.0
	(100 mg/kg)	324.2 ± 12.5	287.9 ± 11.8	250.2 ± 12.0	215.6 ± 12.3	196.2 ± 11.4
7 g	(50 mg/kg)	325.5 ± 14.2	292.5 ± 13.0	255.3 ± 13.1	231.5 ± 12.8	201.5 ± 12.0
	(100 mg/kg)	325.5 ± 14.2	280.8 ± 12.5	245.0 ± 12.6	210.8 ± 12.1	182.4 ± 12.5
7 h	(50 mg/kg)	324.2 ± 12.5	303.5 ± 13.4	278.0 ± 12.5	240.6 ± 12.8	218.5 ± 14.7
	(100 mg/kg)	324.2 ± 12.5	287.9 ± 11.8	250.2 ± 12.0	215.6 ± 12.3	196.2 ± 11.4
8b	(50 mg/kg)	330.0 ± 12.2	299.5 ± 12.3	272.7 ± 13.2	235.6 ± 13.2	205.7 ± 12.5
	(100 mg/kg)	328.8 ± 11.8	280.8 ± 13.6	238.5 ± 11.4	201.5 ± 12.3	178.5 ± 10.9
8c	(50 mg/kg)	330.7 ± 12.4	303.9 ± 14.5	281.3 ± 12.5	255.5 ± 11.5	228.8 ± 12.3
	(100 mg/kg)	315.4 ± 13.5	268.6 ± 14.1	230.4 ± 12.7	210.6 ± 12.5	186.5 ± 12.8

## In Silico study

### Retrieval of the 3D structures of receptor proteins

The hypoglycemic effects of new tetrahydro acridine derivatives that were compatible with the target binding site’s characteristics in the molecular docking investigation were shown in this work using the three-dimensional (dipeptidyl peptidase-IV (PDB ID: 4A5S), sodium-dependent glucose cotransporter (PDB ID: 3DH4) and Glucose transporters GLUT2 (PDB ID: 4PYP) were retrieved from the RCSB Protein Data Bank (https://www.rcsb.org/).

### Ligand selection and receptor optimization

Thirty-two novel tetrahydroacridine derivatives used as ligands. Avogadro software used to minimize energy of ligands and saved it in PDB formate. For the ideal and precise docking, the receptor was optimised by eliminating water molecules, adding hydrogen atoms. Next, the minimised structures were compared to peptides that had been created.

### Molecular docking

The most important stage in silico drug development was active site prediction^53^. The DPP-IV, SGLT1, and GLUT2 active sites were each subjected to independent molecular docking of 32 ligands. The chosen receptor proteins’ active sites were predicted using MGL’s site finding tool (Auto Dock 1.5.7). To compute the relations between ligands and receptor proteins, the parameters left at their default settings. High dG and Ligand Efficiency values indicate that the compound achieves strong binding relative to its size, which is crucial for optimizing drug candidates. High pIC50 and pKd values suggest stronger potency and binding affinity of the compound, making it a more promising drug candidate^54,55^. The best dG, root mean squared deviation (RMSD), and energy validation rankings were used to choose the most suitable. AutoDock Vina and KDeep webserver were used to dock all of the developed peptides against the receptor proteins.

Because molecular docking provides structure-based interactions between ligand and receptor proteins, it has expedited the drug development process. To be docked as ligands, thirty-two new tetrahydroacridines were created. These receptor proteins were chosen because they play important roles in diabetes mellitus and glucose homeostasis. In this work, thirty-two compounds derived from acridines are docked against SGLT1, GLUT2, and DPP IV as inhibitors. KDeep webserver and AutoDock 1.5.7 software were used to conduct the docking analysis. As shown in Tables 4, 5 and 6, respectively, the computational docking findings demonstrated the efficacy of thirteen compounds as excellent inhibitors. The top thirteen conformations were chosen in light of their structural relationships, dG [kcal/mol], Ligand efficiency [kcal/mol], pIC50 and pKd.

**Table 4:**
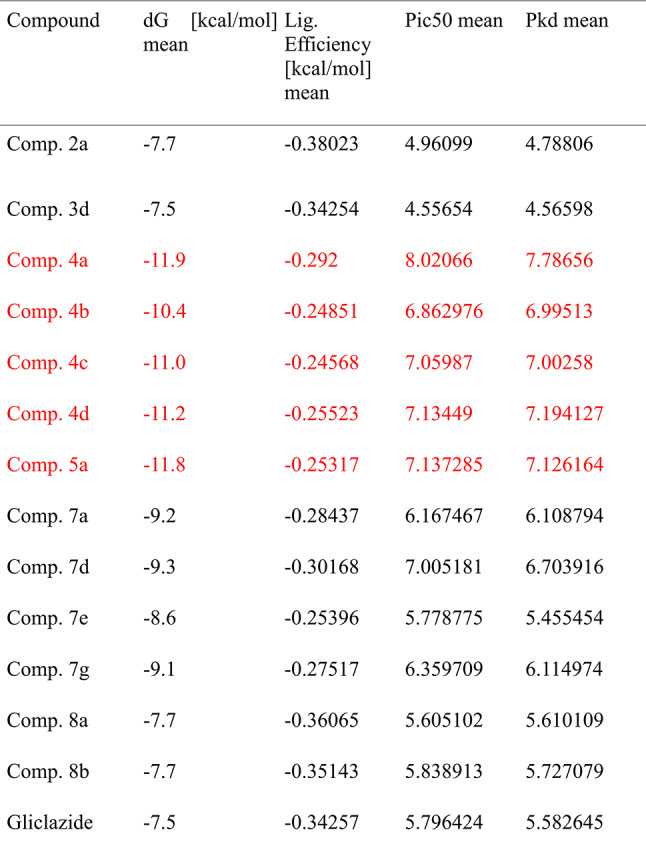
The relationships between DPP-IV (PDB Id: 4a5s) and the top thirteen synthesised acrylidines.

**Table 5:**
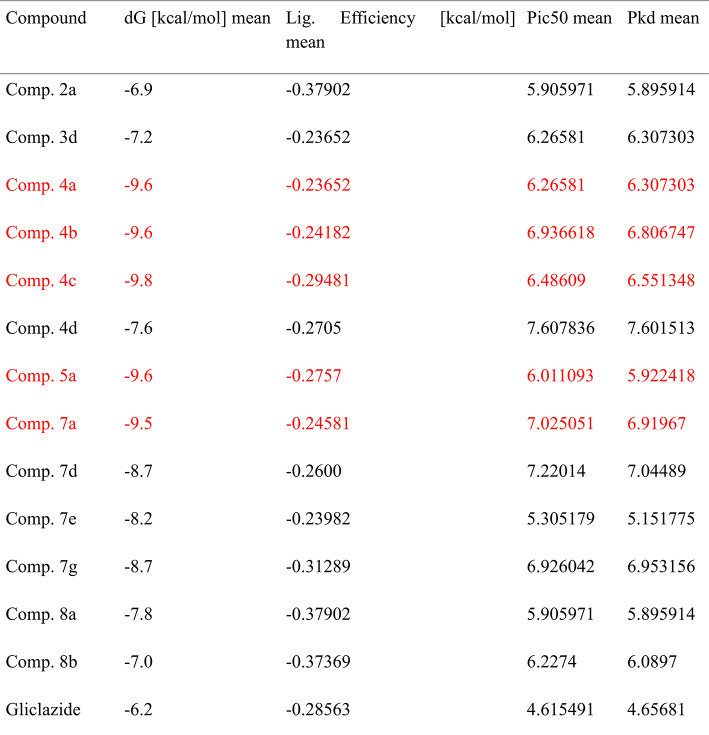
The interactions of top thirteen devised acrylidines with SGLT1 (PDB Id: 3dh4).

**Table 6:**
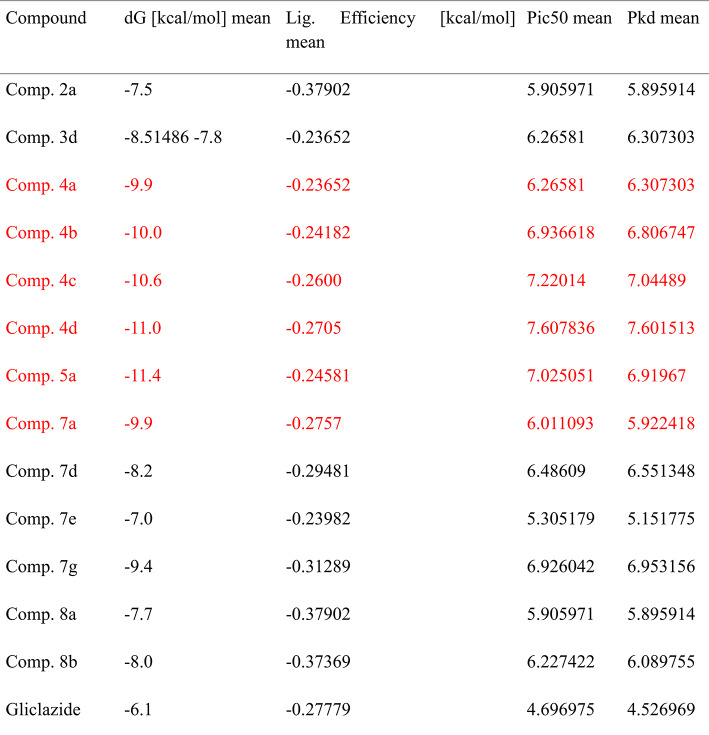
The relationships between GLUT2 (PDB Id : 4pyp) and the top thirteen synthesised acrylidines.

To our knowledge, there are no published docking studies that simultaneously evaluate the binding of gliclazide with DPP-IV, SGLT1, and GLUT2. While gliclazide’s interactions with metabolic enzymes (e.g., CYP2C9/CYP2C19) have been studied, its binding profiles against these transporters/cotransporters relevant to glucose handling remain unreported. Thus, in this study we include gliclazide as a reference ligand for docking against these targets, in order to benchmark the activity of our novel THA derivatives.

### Interaction analysis

**Fig. 4:**
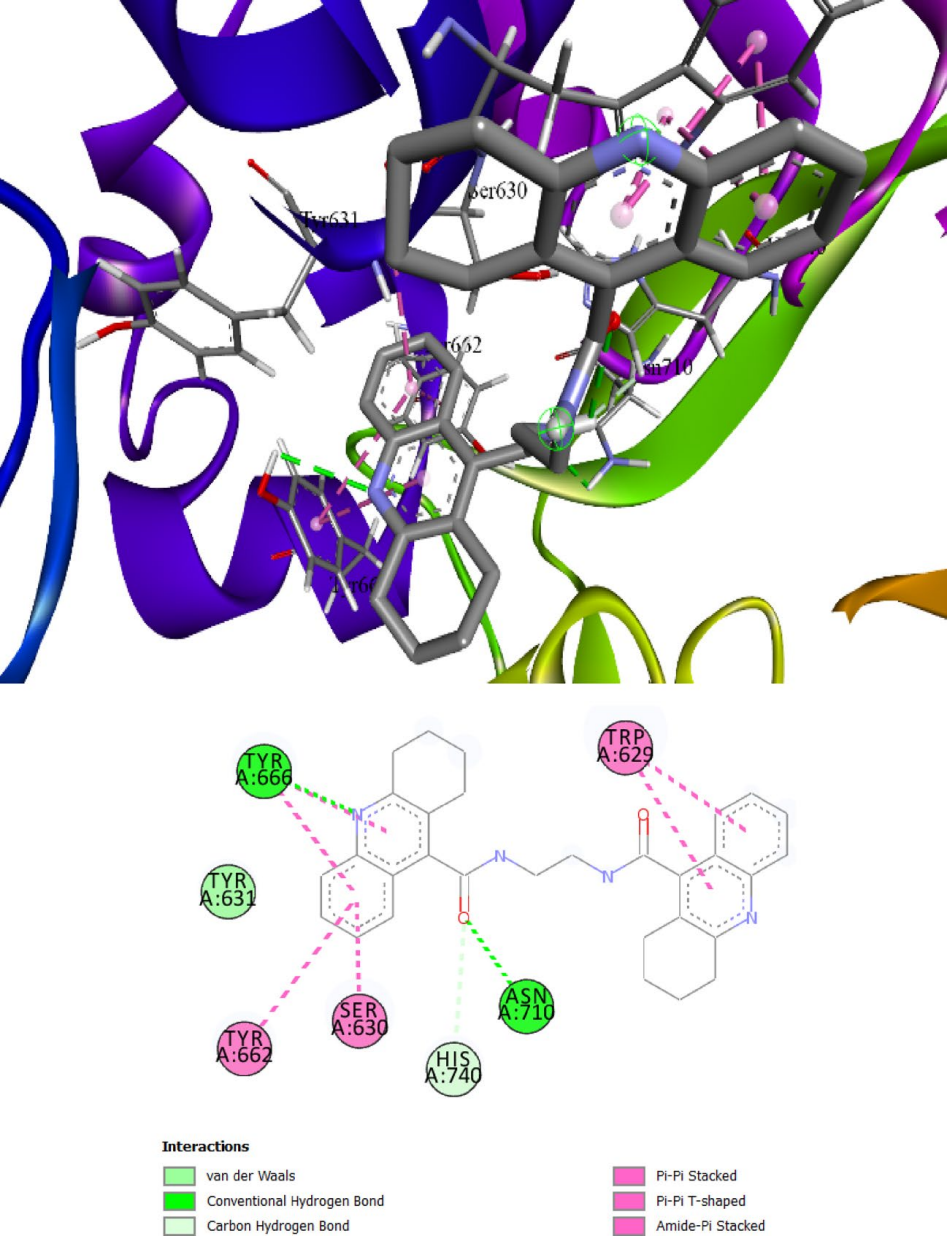
The best-chosen derivatives’ interactions (on the left) and binding patterns (on the right). **Comp. 4a’**s interactions and binding patterns with DPP-IV (**a**, **b**).

The binding pocket of DPP-IV receptor protein contains Glu205, Glu206, Tyr666, Tyr547, His740, Trp629 and Tyr662 as main interacting amino acids. Table 7. lists the top five ligands with the best dG and interactions with these active amino acids out of the threeteen docked ligands used in this investigation. **Comp. 4a** showed the best interaction (binding affinity − 11.9 kcal/mol) with the receptor protein, and Tyr666, Asn710, His740, Trp629, Tyr662, Ser630 and Tyr631 were found to be the leading interactive residues in these interactions (Fig. 4a). The binding mode of the **Comp. 4a** within the binding pocket of DPP-IV is shown in Fig. 3b. The other four derivatives **Comp. 5a, Comp. 4d, Comp. 4c** and **Comp. 4b** with docking scores of − 11.8,− 11.2, 11.0 and − 10.4 showed potent interactions with active amino acids of the binding pocket of DPP-IV (Figures S1-S4 of the Supplementary file).

**Table 7:**
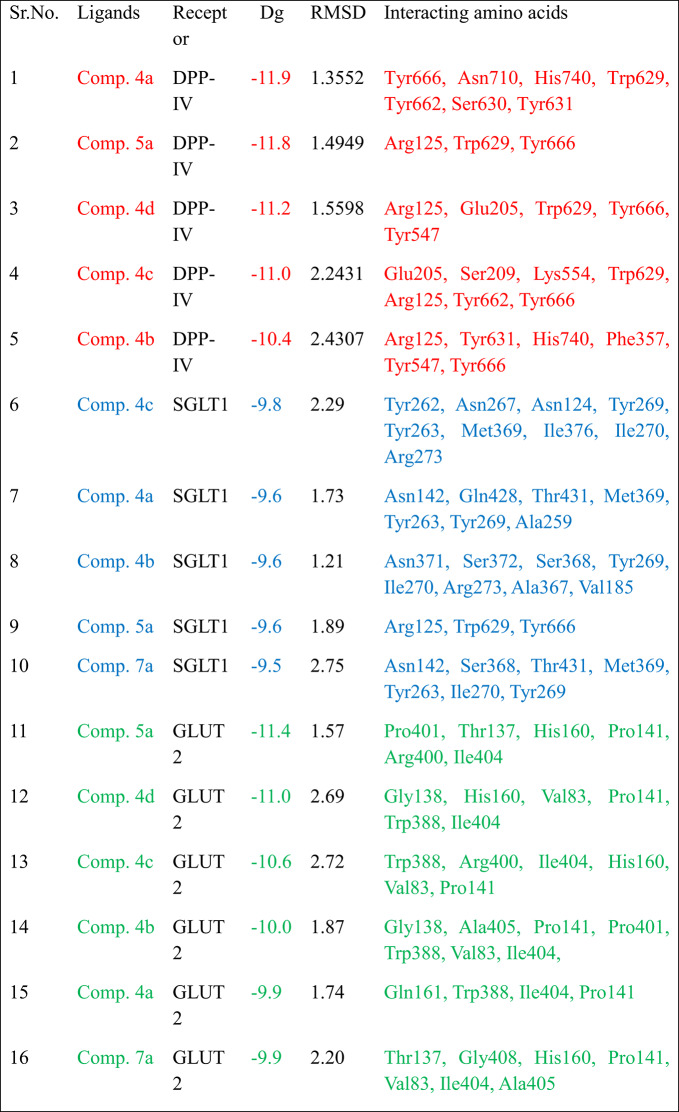
Property profile of selected derivatives against five selected receptor proteins.

**Fig. 5:**
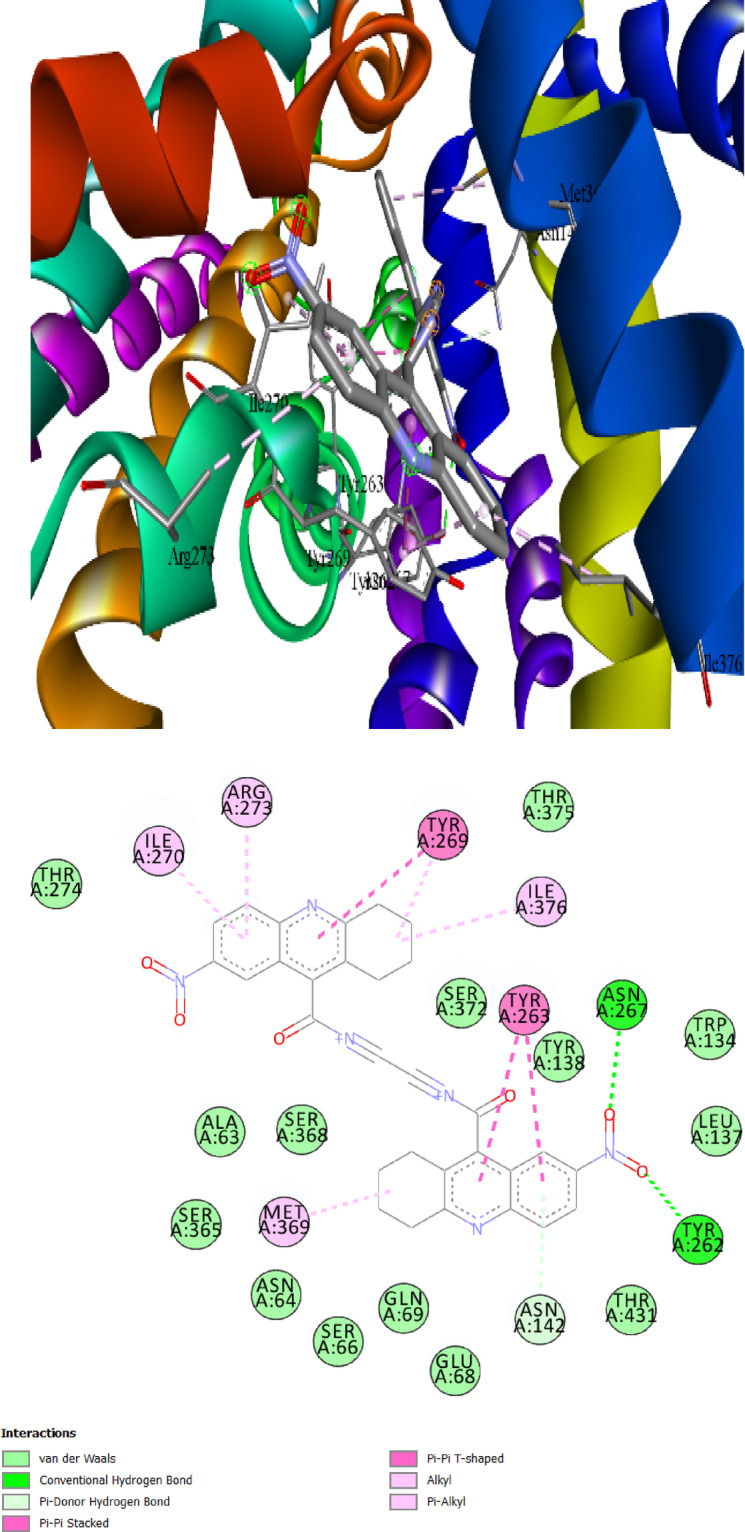
The best-chosen derivatives’ interactions (on the left) and binding patterns (on the right). (**a**,**b**): **Comp. 4c’s** interactions and binding pattern with SGLT1.

The primary interacting amino acids in the binding pocket of the SGLT1 receptor protein are Tyr267, Ile270, Tyr263, Ser368, and Tyr269 instead. In the case of **Comp. 4c** showed best interactions (binding affinity: − 9.8 kcal/mol) with the SGLT1 receptor, two conventional Hydrogen bonds are formed at distances of 3.02 and 3.17 Å between O with Tyr262 and Asn267. Moreover, three Pi-Pi bonds are formed between Tyr269 and two Tyr263. Two alkyl and one Pi-alkyl bonds between cyclohexyl ring with Met369, Ile376 and Tyr269. Another two Pi-alkyl bond between Ile270 and Arg273 with 6-ring. Figure 5b depicts the binding mechanism of this derivative inside the binding pocket of SGLT1 and Fig. 5a shows the primary interactive residues in these interactions. Figures (S5-S8) depict the binding location of the investigated compounds (**Comp. 4a, Comp. 4b, Comp. 5a and Comp. 7a**) in the active site of 3DH4 interaction 3D and 2D.

**Fig. 6:**
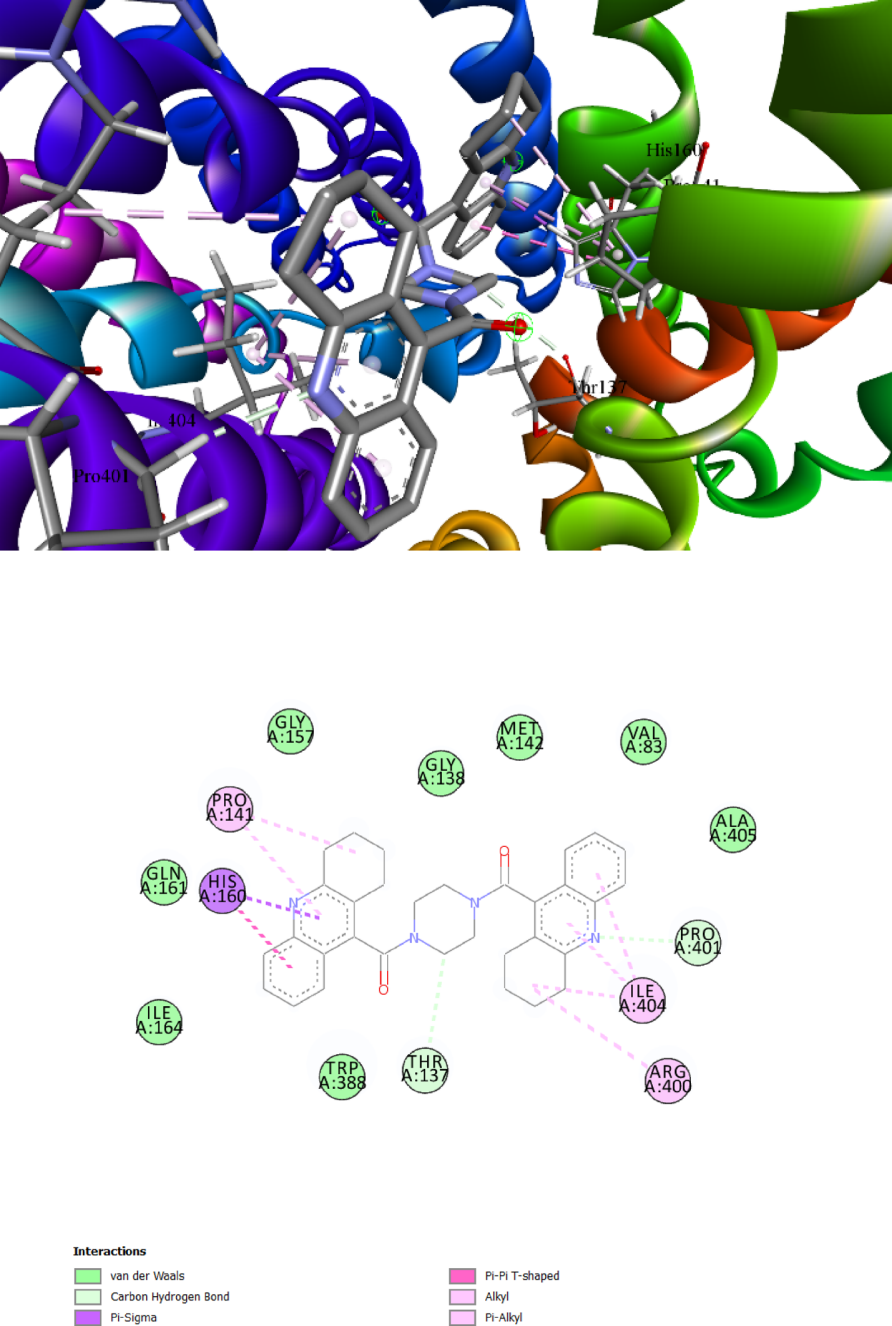
The best-chosen derivatives’ interactions (on the left) and binding patterns (on the right). (**a**,**b**) **Comp5a’s** binding pattern and interaction with GLUT2.

The intestinal and renal absorption and reabsorption of glucose are significantly influenced by glucose transporters. Pro141 and Ile404 were identified as a primary interacting amino acid in the present investigation and shown substantial interactions with the GLUT2 protein. With a dG of − 11.4 kcal/mol, **Comp. 5a** showed promise as therapeutic candidate among the chosen ligands. It had the highest binding affinity for Pro141, Ile404, Pro401, Thr137, His160, and Arg400. With the highest binding affinities and S-scores, the remaining ligands may also be the most effective GLUT2 inhibitors and be promising therapeutic options (Table 6) (Figs. 6a, b). Other promising derivatives (**Comp. 4d, Comp. 4c, Comp. 4b, Comp. 4a and Comp. 7a**) showed binding affinity: − 11.0, − 10.6, − 10.0, − 9.9 and − 9.9, respectively and its 3D interactions and binding mode illustrated in (Figures S9-S13).

For benchmarking, gliclazide was included as a reference ligand in our docking experiments. Gliclazide docking yielded moderate binding affinities against DPP-IV, SGLT1 and GLUT2 (ΔG =  − 7.5, − 6.2 and − 6.1 kcal·mol⁻^1^, respectively; Supplementary Table Sx). In comparison, several of our synthesized THA derivatives (e.g., **Comp. 4a**: ΔG =  − 11.9 kcal·mol⁻^1^ against DPP-IV; **Comp. 5a**: ΔG =  − 11.8 kcal·mol⁻^1^ against DPP-IV; **Comp. 5a**: ΔG =  − 11.4 kcal·mol⁻^1^ against GLUT2) exhibited substantially stronger predicted affinities, suggesting that these THA derivatives may bind more favorably to the selected diabetic targets than gliclazide. The docking results of gliclazide with DPP-IV, SGLT1, and GLUT2 are presented in Supplementary Figures S14–S16, where both 2D and 3D interaction maps illustrate the binding orientations and the key residues involved. This observation supports the potential of the described scaffolds as multitarget candidates; nevertheless, docking predictions are indicative and require enzymatic inhibition assays to confirm functional inhibition.

### Drug scan and ADMET profiling

SwissADME was used to identify the drug-likeness qualities in accordance with Lipinski’s rule of five^56^. Only molecules that meet all five parameters of these criteria can be considered as possible drug candidates. The ADMET-based chemical screening of specific drug candidates was conducted using the online bioinformatic server admetSAR^57^

32 chosen ligands were employed in this investigation, six of tetrahydro acrylidine (**Comp. 4a-d, Comp. 5a** and** Comp. 7a**) were found to be effective inhibitors of DPPI-IV, SGLT1, and GLUT2 as all these derivatives have no violated (**Comp. 4a** and **Comp. 7a**) or only violated one parameter (**Comp. 4d** and **Comp. 5a**) or two violated parameter (**Comp. 4b** and **Comp. 4c**) (Table 8.). Based on Lipinski’srule, **Comp. 4a** and **Comp. 7a** expected for having reasonable oral bioavailability. All six ligands were sent to the admetSAR system for evaluation of the five ADMET profile characteristics (absorption, distribution, metabolism, excretion, and toxicity) in order to further validate the drug-like behaviour of the chosen ligands. All of the chosen derivatives are non-AMES hazardous (with the exception of **Comp. 4c**) and non-carcinogenic, according to the data (Table 9). These lead ligands’ overall ADMET drug scanning findings were too acceptable, and these derivatives particularly **Comp. 7a** could be approved as effective therapeutic candidates against certain receptor proteins.

**Table 8:** According to Lipinski’s criterion, the suggested drug candidates’ drug-like qualities.

Compounds	Target	MW	HBD	HBA	N rot b	LogP	Log S	TPSA	Violations
Comp. 4a	DPP-IV/ SGLT1/ GLUT2	478.58	2	4	7	3.52	− 6.01	83.98	0
Comp. 4b	DPP-IV/ SGLT1/ GLUT2	547.47	2	4	7	4.44	− 7.21	83.98	2
Comp. 4c	DPP-IV/ SGLT1/ GLUT2	568.58	2	8	9	1.84	− 6.16	175.62	2
Comp. 4d	DPP-IV/ GLUT2	506.64	2	4	7	3.90	− 6.63	83.98	1
Comp. 5a	DPP-IV/ SGLT1/ GLUT2	504.62	0	4	4	3.90	− 6.44	66.40	1
Comp. 7a	SGLT1/ GLUT2	388.46	3	3	8	2.83	− 4.25	83.12	0

**Table 9:** The overall results of ADMET drug scanning of these lead ligands.

	Comp. 4a	Comp. 4b	Comp. 4c	Comp. 4d	Comp. 5a	Comp. 7a
Absorption Blood-brainbarrier	+	+	+	+	+	+
Humanintestinal absorption	+	+	+	+	+	+
Caco-2 permeability	+	+	−	+	+	+
P-Glycoprotein substrate	S	S	S	S	S	S
P-Glycoprotein Inhibitor	Non-I	Non-I	Non-I	Non-I	I	Non-I
Renal organic Cation transporter	Non-I	Non-I	Non-I	Non-I	I	Non-I
Metabolism CYP450 2C9 substrate	Non-S	Non-S	Non-S	Non-S	Non-S	Non-S
CYP450 2D6 substrate	Non-S	Non-S	Non-S	Non-S	S	Non-S
CYP450 3A4 substrate	Non-S	Non-S	Non-S	Non-S	Non-S	Non-S
CYP450 1A2 inhibitor	I	I	Non-I	I	Non-I	Non-I
CYP450 2C9 inhibitor	Non- I	Non- I	Non-I	Non-I	I	Non-I
CYP450 2D6 inhibitor	Non- I	Non- I	Non-I	Non-I	Non-I	Non-I
CYP450 2C19 inhibitor	Non- I	I	Non-I	Non-I	I	Non-I
CYP450 3A4 inhibitor	Non- I	Non- I	I	Non-I	I	Non-I
CYP inhibitory promiscuity	Low	High	Low	Low	High	Low
Toxicity AMES toxicity	Non	Non	AMES Toxic	Non	Non	Non
Carcinogens	Non	Non	Non	Non	Non	Non

## Structure–activity relationship (SAR)

The SAR analysis revealed that electron-withdrawing substituents (Cl, Br) on the tetrahydroacridine ring generally enhanced antidiabetic activity, likely due to improved polar interactions within biological targets. In contrast, electron-donating groups (e.g., CH_3_) showed moderate activity.

Regarding linkers, compounds bearing a piperazine bridge **(5a–d)** exhibited better *in-vivo* efficacy and docking scores, reflecting their enhanced flexibility and optimal spatial orientation. Hydrazide-linked derivatives **(4a–d)** were potent *in-vitro* but showed reduced *in-vivo* performance, possibly due to limited permeability. Meanwhile, urea/thiourea derivatives **(7a–h)** with phenyl termini demonstrated strong multitarget potential, particularly thiourea analogs, benefiting from stronger sulfur-based interactions.

The docking results supported experimental findings, where top-performing **compounds (4a, 4b, 5a, 7a)** showed stable interactions with critical residues in DPP-IV, SGLT1, and GLUT2. Overall, the SAR findings emphasize the role of scaffold modification, linker flexibility, and substituent electronics in modulating biological activity.

## Experimental

Merck, Aldrich, and Fluka were the suppliers of all commercially available reagents. Thin layer chromatography (TLC) was used to monitor all reactions. Using a Kofler melting points device, melting points were found and left uncorrected. IR spectra were obtained on a Nicolet 710 FT-IR spectrometer (KBr, v_max_ in cm^−1^). A Bruker Bio Spin AG spectrometer was used to record the ^1^H NMR and ^13^C NMR spectra of each chemical in DMSO-d6 at 400 MHz and 100 MHz, respectively. Chemical shifts (δ) were given in parts per million (ppm) with reference to tetramethylsilane (TMS) as an internal standard (δ = 0); coupling constants (*J*) were given in hertz (Hz). TMS (δ = 0) or DMSO (δ = 39.51) were used as internal standards for ^13^C NMR, and spectra were acquired with full proton decoupling. A Perkin-Elmer CHN-analyzer model was used to acquire elemental analyses.

### General procedure for the synthesis of 1,2,3,4-tetrahydroacridine-9-carboxylic acid derivatives 2a-d

The pure product 1,2,3,4-tetrahydrocridine-9-carboxylic acid derivative 2a-d was obtained by stirring a mixture of isatin derivative 1a-d (0.001 mol) in ethanol (5 mL) and potassium hydroxide (0.25 g in water 5 mL) at room temperature for 15 to 30 min, acidifying the mixture with concentrated hydrochloric acid, adding cyclohexanone (0.103 mL, 0.001 mol) and CuSO_4_.7H_2_O (0.025 g, 0.1 mmol) until a precipitate formed. The reaction progress was tracked using TLC (CHCl_3_/MeOH 9:1).

### 4-Tetrahydroacridine-9-carboxylic acid 2a

Pale yellow needles, mp: 282–285 °C ^28^, (0.178 g, 78%); (C_14_H_13_NO_2_ M.wt = 227.12); Calcd C:74.0, H: 5.72, N: 6.16; Found C: 73.64, H: 5.44, N: 5.89; IR (KBr, v, cm^−1^): 3467–3318 (broad band OH), 3045 (CH_arom._), 2933 (CH_aliphatic_), 1642 (C = O); ^1^H-NMR (400 MHz, δ, CDCl_3_): δ 1.22–1.36 (m, 4H, 2CH_2_), 1.82–1.77 (t, *J* = 8.0 Hz, 2H, CH_2_), 1.93–1.87 (t, *J* = 8.0 Hz, 2H, CH_2_), 7.34–7.60 (m, 4H, CH_arom_), 12.24 (br, 1H, COOH); ^13^C NMR (100 MHz, CDCl_3_): δ 22.90, 23.20, 29.10, 33.55, 125.50, 126.67, 127.32, 128.45, 130.95, 132.34, 135.11, 146.67, 151.35, 168.65; MS (EI, m/z): 227 M^+^.

### 7-Bromo-1,2,3,4-tetrahydroacridine-9-carboxylic acid 2b

Pale yellow crystals, mp: 240–42 °C, (0.25 g, 82%); (C_14_H_12_BrNO_2_, MWt = 306.15); Calcd C:54.90, H: 3.92, N: 4.57, Br: 26.11; Found C: 54.63, H: 3.75, N: 4.44, Br: 25.88; IR (KBr, v cm^−1^): 3460–3310 (broad band OH), 3056 (CH_arom._), 2942 (CH_aliphatic_), 1664 (C=O); ^1^H-NMR (400 MHz, δ, CDCl_3_): δ 1.25–1.35 (m, 4H, 2CH_2_), 1.77–1.82 (t, *J* = 8.0 Hz, 2H, CH_2_), 1.87–1.92 (t, *J* = 8.0 Hz, 2H, CH_2_), 7.35–7.48 (m, 3H, CH_arom_), 11.66 (br, 1H, COOH); ^13^C NMR (100 MHz, CDCl_3_): δ 22.90, 23.20, 29.10, 33.55, 125.50, 126.67, 127.32, 128.45, 130.95, 132.34, 135.11, 146.67, 150.35, 168.65; MS (EI, m/z): 308 M^+2^, 306 M^+^.

### 7-Chloro-1,2,3,4-tetrahydroacridine-9-carboxylic acid 2c

Pale yellow crystals, mp: 240–42 °C, (0.25 g, 82%); (C_14_H_12_ClNO_2_, MWt = 261.06); Calcd C:64.25, H: 4.62, N: 5.35, Cl 13.55; Found C: 64.11, H: 4.40, N: 5.08, Cl 13.07; IR (KBr, v cm^−1^): 3460–3310 (broad band OH), 3056 (CH_arom._), 2942 (CH_aliphatic_), 1664 (C=O); ^1^H-NMR (400 MHz, δ, CDCl_3_): δ 1.25–1.35 (m, 4H, 2CH_2_), 1.77–1.82 (t, *J* = 8.0 Hz, 2H, CH_2_), 1.87–1.92 (t, *J* = 8.0 Hz, 2H, CH_2_), 7.35–7.48 (m, 3H, CH_arom_), 12.36 (br, 1H, COOH); ^13^C NMR (100 MHz, CDCl_3_): δ 22.90, 23.20, 29.10, 33.55, 125.50, 126.67, 127.32, 128.45, 130.95, 132.34, 135.11, 146.67, 150.35, 168.65; MS (EI, m/z): 261 M^+2^, 259 M^+^.

### 7-Methyl-1,2,3,4-tetrahydroacridine-9-carboxylic acid 2d

Yellow crystals, mp: 228–30 °C, (0.25 g, 80%); (C_15_H_15_NO_2_, MWt = 241.11); Calcd C: 74.67, H: 6.27, N: 5.81; Found C: 74.11, H: 5.42, N: 5.25; IR (KBr, v, cm^−1^): 3450–3300 (broad band OH), 3043 (CH_arom._), 2942 (CH_aliphatic_), 1664 (C=O); ^1^H-NMR (400 MHz, δ, CDCl_3_): δ 1.22–1.30 (m, 4H, 2CH_2_), 1.74–1.82 (t, *J* = 8.0 Hz, 2H, CH_2_), 1.85–1.92 (t, *J* = 8.0 Hz, 2H, CH_2_), 2.11 (s, 3H, CH_3_), 7.30–7.45 (m, 3H, CH_arom_), 12.55 (br, 1H, COOH); ^13^C NMR (100 MHz, CDCl_3_): δ 22.90, 23.20, 29.10, 33.55, 42.15, 125.50, 126.67, 127.32, 128.45, 130.95, 132.34, 135.11, 146.67, 150.35, 168.65; MS (EI, m/z):, 241 M^+^.

### Synthesis of 1,2,3,4-tetrahydroacridine-9-carbonyl chloride derivatives 3a-d

17.8 g (10.9 mL, 0.150 mol) of freshly distilled thionyl chloride is added to the reaction flask. Tetrahydroacridine-9-carboxylic acid derivative 2a-c (0.10 mol) is added in several amounts while stirring. Since the final additions are first insoluble, using a magnetic stirrer to mix them is momentarily impossible. In an oil bath, the reaction mixture is first heated gradually while being stirred (as much as possible) to a bath temperature of 50 °C. After that, stirring is maintained for two more hours at a bath temperature of 80 °C. Following the reaction mixture’s cooling, a distillation bridge takes the place of the reflux condenser, and the excess thionyl chloride is eliminated by distillation at a lower pressure (about 20 hPa). The residue is a yellowish solid.

### 4-Tetrahydroacridine-9-carbonyl chloride 3a

Yellow crystals, mp: 182–185 °C, (C_14_H_12_ClNO, MWt = 245.7), Calcd C: 68.44, H: 4.92, N: 5.70, Cl 14.43; Found: C: 67.88, H: 4.50, N: 5.45, Cl 14.02; IR (KBr, v, cm^−1^): 3044 (CH_arom._), 2936 (CH_aliphatic_), 1748 (C=O), 668 (C–Cl); ^1^H-NMR (400 MHz, δ, CDCl_3_): δ 1.24–1.30 (m, 4H, 2CH_2_), 1.78–1.83 (t, *J* = 8.0 Hz, 2H, CH_2_), 1.87–1.92 (t, *J* = 8.0 Hz, 2H, CH_2_), 7.38–7.55 (m, 4H, CH_arom_); ^13^C NMR (100 MHz, CDCl_3_): δ 22.66, 23.20, 28.12, 32.15, 125.45, 126.40, 127.44, 128.32, 131.25, 132.16, 135.22, 147.55, 151.45, 183.89; MS (EI, m/z): 247 M^+2^.

### 7-Bromo-1,2,3,4-tetrahydroacridine-9-carbonyl chloride 3b

Brownish crystals, mp: 144–46 °C, (C_14_H_11_BrClNO, MWt=322.97), Calcd C: 51.80, H: 3.42, N: 4.32, Br: 24.62, Cl 10.92; Found: C: 51.44, H: 3.02, N: 3.87, Br: 24.12, Cl 10.56; IR (KBr, vcm^−1^): 3046 (CH_arom._), 2956 (CH_aliphatic_), 1755 (C=O), 687 (C–Cl); ^1^H-NMR (400 MHz, δ, CDCl_3_): δ 1.24–1.28 (m, 4H, 2CH_2_), 1.77–1.82 (t, *J*=8.0 Hz, 2H, CH_2_), 1.88–1.92 (t, *J* = 8.0 Hz, 2H, CH_2_), 7.35–7.53 (m, 3H, CH_arom_); ^13^C NMR (100 MHz, CDCl_3_): δ 22.23, 23.18, 28.17, 32.65, 123.43, 126.44, 127.67, 128.65, 131.28, 132.20, 135.28, 147.30, 151.41, 188.60; MS (EI, m/z): 326 M^+2^.

### 7-Chloro-1,2,3,4-tetrahydroacridine-9-carbonyl chloride 3c

Brownish crystals, mp: 144–46 °C, (C_14_H_11_Cl_2_NO, MWt = 297.02), Calcd C: 60.02, H: 3.96, N: 5.00, Cl 23.31; Found: C: 59.49, H: 3.62, N: 4.66, Cl 22.87; IR (KBr, v, cm^−1^): 3046 (CH_arom._), 2956 (CH_aliphatic_), 1755 (C=O), 687 (C–Cl); ^1^H-NMR (400 MHz, δ, CDCl_3_): δ 1.24–1.28 (m, 4H, 2CH_2_), 1.77–1.82 (t, *J* = 8.0 Hz, 2H, CH_2_), 1.88–1.92 (t, *J* = 8.0 Hz, 2H, CH_2_), 7.35–7.53 (m, 3H, CH_arom_); ^13^C NMR (100 MHz, CDCl_3_): δ 22.23, 23.18, 28.17, 32.65, 123.43, 126.44, 127.67, 128.65, 131.28, 132.20, 135.28, 147.30, 151.41, 188.60; MS (EI, m/z): 282 M^+2^.

### 7-Methyl-1,2,3,4-tetrahydroacridine-9-carbonyl chloride 3d

Brownish crystals, mp: 134–36 °C, (C_15_H_14_ClNO, MWt = 259.08.02), Calcd C: 69.36, H: 5.43, N: 5.39, Cl 13.65; Found: C: 59.49, H: 3.62, N: 4.66, Cl 22.87; IR (KBr, v, cm^−1^): 3041 (CH_arom._), 2950 (CH_aliphatic_), 1757 (C=O), 685 (C–Cl); ^1^H-NMR (400 MHz, δ, CDCl_3_): δ 1.22–1.28 (m, 4H, 2CH_2_), 1.76–1.82 (t, *J* = 8.0 Hz, 2H, CH_2_), 1.86–1.92 (t, *J* = 8.0 Hz, 2H, CH_2_), 2.12 (s, 3H, CH_3_), 7.35–7.53 (m, 3H, CH_arom_); ^13^C NMR (100 MHz, CDCl_3_): δ 22.23, 23.18, 28.17, 32.65, 42.26, 123.43, 126.44, 127.67, 128.65, 131.28, 132.20, 135.28, 147.30, 151.41, 188.60; MS (EI, m/z): 259 M^+2^.

### Synthesis of N,N’-(Ethane-1,2-diyl)bis(1,2,3,4-tetrahydroacridine-9-carboxamide) derivatives 4a-d

A mixture of compound **3a–d** (0.01 mol) in absolute ethanol (25 mL) was treated with ethylenediamine (07 mL, 0.01 mol) was refluxed for 3 h. Solvent was removed under reduced pressure and the residual mass was then triturated with light petroleum (40–60). The formed solid was collected by filtration and recrystallized from acetonitrile into compounds **4a–d**.

### N,N’-(Ethane-1,2-diyl)bis(1,2,3,4-tetrahydroacridine-9-carboxamide) 4a

Pale yellow crystals, mp: 230–233 °C, yield 70%, (C_30_H_30_N_4_O_2_, MWt = 478.58), Calcd C: 75.29, H: 6.32, N: 11.71; Found: C: 74.88, H: 3.18, N: 11.56; IR (KBr, v, cm^−1^): 3275 (NH), 3051 (CH_arom._), 2948 (CH_aliphatic_), 1668 (C=O); ^1^H-NMR (400 MHz, δ, CDCl_3_): δ 1.25–1.30 (m, 8H, 2CH_2_), 1.78–1.82 (t, *J* = 8.0 Hz, 4H, CH_2_), 1.88–1.92 (t, *J* = 8.0 Hz, 4H, CH_2_), 3.33 (s, 4H, 2CH_2_), 7.32–7.53 (m, 8H, CH_arom_), 11.68 (s, 2H, NH exchangeable with D_2_O); ^13^C NMR (100 MHz, CDCl_3_): δ 22.23, 23.18, 28.17, 32.65, 37.2, 123.43, 126.44, 127.67, 128.65, 131.28, 132.20, 135.28, 147.30, 151.41, 188.60; MS (EI, m/z): 478 M^+^.

### N,N’-(Ethane-1,2-diyl)bis(7-bromo-1,2,3,4-tetrahydroacridine-9-carboxamide) 4b

Orange needles, mp: 196–198 °C, yield 78%, (C_30_H_28_Br_2_N_4_O_2_, MWt = 634.06), Calcd C: 56.62, H: 4.43, N: 8.80, Br: 25.11; Found: C: 56.22, H: 4.15, N: 8.55, Br: 24.89; IR (KBr, v, cm^−1^): 3272 (NH), 3055 (CH_arom._), 2936 (CH_aliphatic_), 1665 (C=O); ^1^H-NMR (400 MHz, δ, CDCl_3_): δ 1.25–1.31 (m, 8H, 2CH_2_), 1.77–1.82 (t, *J* = 8.2 Hz, 4H, CH_2_), 1.88–1.92 (t, *J* = 8.2 Hz, 4H, CH_2_), 3.34 (s, 4H, 2CH_2_), 7.35–7.50 (m, 6H, CH_arom_), 11.45 (s, 2H, NH exchangeable with D_2_O); ^13^C NMR (100 MHz, CDCl_3_): δ 22.20, 23.12, 28.15, 32.61, 37.0, 123.41, 126.40, 127.61, 128.60, 131.25, 132.25, 135.25, 147.33, 151.44, 186.68; MS (EI, m/z): 638 M^+2^.

### N,N’-(Ethane-1,2-diyl)bis(7-chloro-1,2,3,4-tetrahydroacridine-9-carboxamide) 4c

Brown powder, mp: 208–210 °C, yield 74%, (C_30_H_28_Cl_2_N_4_O_2_, MWt = 546.16), Calcd C: 65.82, H: 5.15, N: 10.23, Cl 12.95; Found: C: 65.67, H: 4.90, N: 10.01, Cl 12.64; IR (KBr, v, cm^−1^): 3187 (2NH), 3064 (CH_arom._), 2956 (CH_aliphatic_), 1677 (C=O; ^1^H-NMR (400 MHz, δ, CDCl_3_): δ 1.25–1.33 (m, 8H, 2CH_2_), 1.78–1.82 (t, *J* = 8.2 Hz, 4H, CH_2_), 1.88–1.93 (t, *J* = 8.2 Hz, 4H, CH_2_), 7.38–7.55 (m, 6H, CH_arom_), 12.77 (s, 2H, NH exchangeable with D_2_O); ^13^C NMR (100 MHz, CDCl_3_): δ 22.15, 23.18, 28.25, 32.72, 37.3, 42.02, 123.55, 126.48, 127.78, 128.67, 131.33, 132.41, 135.32, 147.45, 151.53, 169.66, 188.22; MS (EI, m/z): 546 M^+^.

### N,N’-(Ethane-1,2-diyl)bis(7-methyl-1,2,3,4-tetrahydroacridine-9-carboxamide) 4d

Brown powder, mp: 203–205 °C, yield 70%, (C_32_H_34_N_4_O_2_, MWt = 506.27), Calcd C: 75.86, H: 6.76, N: 11.06; Found: C: 75.67, H: 6.50, N: 10.88; IR (KBr, v, cm^−1^): 3187 (2NH), 3064 (CH_arom._), 2956 (CH_aliphatic_), 1677 (C=O; ^1^H-NMR (400 MHz, δ, CDCl_3_): δ 1.25–1.33 (m, 8H, 2CH_2_), 1.78–1.82 (t, *J* = 8.2 Hz, 4H, CH_2_), 1.88–1.93 (t, *J* = 8.2 Hz, 4H, CH_2_), 2.12 (s, 6H, 2CH_3_), 7.38–7.55 (m, 6H, CH_arom_), 12.77 (s, 2H, NH exchangeable with D_2_O); ^13^C NMR (100 MHz, CDCl_3_): δ 22.15, 23.18, 28.25, 32.72, 42.02, 123.55, 126.48, 127.78, 128.67, 131.33, 132.41, 135.32, 147.45, 151.53, 169.66, 188.22; MS (EI, m/z): 546 M^+^.

### Synthesis of piperazine-1,4-diylbis((1,2,3,4-tetrahydroacridin-9-yl)methanone) derivatives 5a-d

A solution of piperazine (1 g, 0.125 mol) in absolute ethanol 50 mL was treated with compound **3a-d** (0.1 mol). The reaction mixture was heated under reflux for 1 h then left to cool. The white needles of compound **5a-d** was collected by filtration, washed with cold ethanol and dried.

### Piperazine-1,4-diylbis((1,2,3,4-tetrahydroacridin-9-yl)methanone) 5a

White needles, mp: 282–284 °C, yield 74%, (C_32_H_32_N_4_O_2_, MWt = 504.62), Calcd C: 76.16, H: 6.39, N: 11.10; Found: C: 75.65, H: 6.11, N: 10.88; IR (KBr, v, cm^−1^): 3044 (CH_arom._), 2962 (CH_aliphatic_), 1678 (C=O); ^1^H-NMR (400 MHz, δ, CDCl_3_): δ 1.26–1.32 (m, 8H, 2CH_2_), 1.78–1.82 (t, *J* = 8.0 Hz, 8H, CH_2_), 3.34 (s, 8H, 4CH_2piprazine_), 7.35–7.50 (m, 8H, CH_arom_); ^13^C NMR (100 MHz, CDCl_3_): δ 23.80, 27.12, 31.15, 32.61, 37.00, 39.11, 122.60, 126.40, 127.61, 128.60, 131.05, 132.11, 135.13, 147.45, 151.44, 186.68; MS (EI, m/z): 504 M^+^.

### Piperazine-1,4-diylbis((7-bromo-1,2,3,4-tetrahydroacridin-9-yl)methanone) 5b

Light brown sheets, mp: 260–262 °C, yield 76%, (C_32_H_30_Br_2_N_4_O_2_, MWt = 660.07), Calcd C: 58.02, H: 4.56, N: 8.46, Br: 24.13; Found: C: 57.88, H: 4.22, N: 8.11, Br: 23.88; IR (KBr, v, cm^−1^): 3056 (CH_arom._), 2960 (CH_aliphatic_), 1676 (C=O); ^1^H-NMR (400 MHz, δ, CDCl_3_): δ 1.27–1.32 (m, 8H, 2CH_2_), 1.78–1.82 (t, *J* = 8.0 Hz, 4H, CH_2_), 1.88–1.92 (t, *J* = 8.0 Hz, 4H, CH_2_), 3.35 (s, 8H, 4CH_2piprazine_), 7.38–7.55 (m, 6H, CH_arom_); ^13^C NMR (100 MHz, CDCl_3_): δ 23.84, 27.10, 38.19, 32.66, 37.09, 39.45, 122.62, 126.54, 127.44, 128.55, 131.12, 132.32, 135.11, 147.43, 158.23, 188.22; MS (EI, m/z): 660 M^+2^.

### Piperazine-1,4-diylbis((7-chloroo-1,2,3,4-tetrahydroacridin-9-yl)methanone) 5c

Greenish crystals, mp: 290–292 °C, yield 70%, (C_32_H_30_Cl_2_N_4_O_2_, MWt = 572.17), Calcd C: 67.02, H: 5.27, N: 9.77, Cl 12.36; Found: C: 66.84, H: 5.03, N: 9.56, Cl 12.04; IR (KBr, v, cm^−1^): 2948 (CH_aliphatic_), 1696 (C=O), 1672 (C=O); ^1^H-NMR (400 MHz, δ, CDCl_3_): δ 1.28–1.32 (m, 8H, 2CH_2_), 1.77–1.82 (t, *J* = 8.0 Hz, 4H, CH_2_), 1.87–1.92 (t, *J* = 8.0 Hz, 4H, CH_2_), 1.18 (s, 6H, 2CH_3_), 3.34 (s, 8H, 4CH_2piprazine_), 7.35–7.50 (m, 6H, CH_arom_; ^13^C NMR (100 MHz, CDCl_3_): δ 23.50,. 27.10, 38.19, 32.66, 37.09, 39.45, 41.88, 122.75, 126.75, 127.52, 128.72, 131.33, 132.44, 135.25, 147.56, 158.44, 182.78, 188.35; MS (EI, m/z): 572 M^+^.

### Piperazine-1,4-diylbis((7-methyl-1,2,3,4-tetrahydroacridin-9-yl)methanone) 5d

Pale yellow crystals, mp: 272–274 °C, yield 70%, (C_34_H_36_N_4_O_2_, MWt = 532.28), Calcd C: 76.66, H: 6.81, N: 10.52; Found: C: 76.34, H: 6.53, N: 10.50; IR (KBr, v, cm^−1^): 2948 (CH_aliphatic_), 1696 (C=O), 1672 (C=O); ^1^H-NMR (400 MHz, δ, CDCl_3_): δ 1.28–1.32 (m, 8H, 2CH_2_), 1.77–1.82 (t, *J* = 8.0 Hz, 4H, CH_2_), 1.87–1.92 (t, *J* = 8.0 Hz, 4H, CH_2_), 1.18 (s, 6H, 2CH_3_), 3.34 (s, 8H, 4CH_2piprazine_), 7.35–7.50 (m, 6H, CH_arom_; ^13^C NMR (100 MHz, CDCl_3_): δ 23.50,. 27.10, 38.19, 32.66, 37.09, 39.45, 41.88, 122.75, 126.75, 127.52, 128.72, 131.33, 132.44, 135.25, 147.56, 158.44, 182.78, 188.35; MS (EI, m/z): 532 M^+^.

### Synthesis of N-(2-hydroxyethyl)-1,2,3,4-tetrahydroacridine-9-carboxamide derivatives 6a-d

Compound 3a-d (0.01 mol) was dissolved in 50 mL absolute ethanol and then was treated with ethanolamine (0.08 mL, 0.0125 mol). The reaction mixture was heated under reflux for 3 h, after reaction completion (as monitored by TLC) ethanol was removed under reduced pressure and the residual mass was then triturated with light petroleum (40–60). The formed solid was collected and recrystallized from acetonitrile into 6a-d.

### N-(2-Hydroxyethyl)-1,2,3,4-tetrahydroacridine-9-carboxamide 6a

Pale yellow crystals, mp: 252–255 °C, yield 65%, (C_16_H_18_N_2_O_2_, MWt = 270.32), Calcd C: 71.09, H: 6.71, N: 10.36; Found: C: 70.66, H: 6.38, N: 10.08; IR (KBr, v, cm^−1^): 3411 (OH), 3252 (NH), 3048 (CH_arom._), 2932 (CH_aliphatic_), 1670 (C=O); ^1^H-NMR (400 MHz, δ, CDCl_3_): δ 1.27–1.32 (m, 4H, 2CH_2_), 1.78–1.82 (t, *J* = 8.1 Hz, 2H, CH_2_), 1.88–1.92 (t, *J* = 8.0 Hz, 2H, CH_2_), 3.23–3.36 (m, 4H, CH_2_), 6.11 (s, 1H, OH exchangeable with D_2_O), 7.30–7.44 (m, 4H, CH_arom_), 11.02 (s, 1H, NH exchangeable with D_2_O); ^13^C NMR (100 MHz, CDCl_3_): δ 21.20, 23.25, 28.33, 32.45, 37.55, 39.60 121.22, 123.43, 127.65, 128.60, 131.38, 132.76, 135.55, 147.56, 151.88, 186.65; MS (EI, m/z): 270 M^+^.

### 7-Bromo-N-(2-hydroxyethyl)-1,2,3,4-tetrahydroacridine-9-carboxamide 6b

Pale yellow crystals, mp: 212–215 °C, yield 65%, (C_16_H_17_BrN_2_O_2_, MWt = 348.05), Calcd C: 55.03, H: 4.91, N: 8.01, Br: 22.87; Found: C: 54.66, H: 4.18, N: 7.76, Br: 22.60; IR (KBr, v, cm^−1^): 3406 (OH), 3245 (NH), 3040 (CH_arom._), 2944 (CH_aliphatic_), 1674 (C=O); ^1^H-NMR (400 MHz, δ, CDCl_3_): δ 1.26–1.30 (m, 4H, 2CH_2_), 1.77–1.82 (t, *J* = 8.1 Hz, 2H, CH_2_), 1.88–1.93 (t, *J* = 8.0 Hz, 2H, CH_2_), 3.25–3.36 (m, 4H, 2CH_2_), 6.10 (s, 1H, OH exchangeable with D_2_O), 7.36–7.48 (m, 3H, CH_arom_), 11.36 (s, 1H, NH exchangeable with D_2_O); ^13^C NMR (100 MHz, CDCl_3_): δ 21.33, 23.65, 28.55, 32.56, 37.63, 39.73 122.23, 123.88, 127.87, 128.67, 131.44, 132.74, 135.43, 147.22, 158.65, 188.32; MS (EI, m/z): 348 M^+^.

### 7-Chloro-N-(2-hydroxyethyl)-1,2,3,4-tetrahydroacridine-9-carboxamide 6c

Pale yellow crystals, mp: 212–215 °C, yield 65%, (C_16_H_17_ClN_2_O_2_, MWt = 304.10), Calcd C: 63.05, H: 5.62, Cl 11.63, N: 9.19; Found: C: 62.80, H: 5.34, Cl 11.38, N: 8.78; IR (KBr, v, cm^−1^): 3412 (OH), 3245 (NH), 3040 (CH_arom._), 2944 (CH_aliphatic_), 1674 (C=O); ^1^H-NMR (400 MHz, δ, CDCl_3_): δ 1.26–1.30 (m, 4H, 2CH_2_), 1.77–1.82 (t, *J* = 8.1 Hz, 2H, CH_2_), 1.88–1.93 (t, *J* = 8.0 Hz, 2H, CH_2_), 3.25–3.36 (m, 4H, 2CH_2_), 6.15 (s, 1H, OH exchangeable with D_2_O), 7.36–7.48 (m, 3H, CH_arom_), 11.36 (s, 1H, NH exchangeable with D_2_O); ^13^C NMR (100 MHz, CDCl_3_): δ 21.33, 23.65, 28.55, 32.56, 37.63, 39.73 122.23, 123.88, 127.87, 128.67, 131.44, 132.74, 135.43, 147.22, 158.65, 188.32; MS (EI, m/z): 304 M^+^.

### N-(2-hydroxyethyl)-7-methyl-1,2,3,4-tetrahydroacridine-9-carboxamide 6d

Pale yellow crystals, mp: 212–215 °C, yield 61%, (C_17_H_20_N_2_O_2_, MWt = 284.15), Calcd C: 71.81, H: 7.09, N: 9.85; Found: C: 71.55, H: 6.72, N: 9.60; IR (KBr, v, cm^−1^): 3412 (OH), 3245 (NH), 3040 (CH_arom._), 2944 (CH_aliphatic_), 1674 (C=O); ^1^H-NMR (400 MHz, δ, CDCl_3_): δ 1.26–1.30 (m, 4H, 2CH_2_), 1.77–1.82 (t, *J* = 8.1 Hz, 2H, CH_2_), 1.88–1.93 (t, *J* = 8.0 Hz, 2H, CH_2_), 2.11, (s, 3H, CH_3_), 3.25–3.36 (m, 4H, 2CH_2_), 6.10 (s, 1H, OH exchangeable with D_2_O), 7.36–7.48 (m, 3H, CH_arom_), 11.36 (s, 1H, NH exchangeable with D_2_O); ^13^C NMR (100 MHz, CDCl_3_): δ 21.33, 23.65, 28.55, 32.56, 37.63, 39.73, 42.23, 122.23, 123.88, 127.87, 128.67, 131.44, 132.74, 135.43, 147.22, 158.65, 188.32; MS (EI, m/z): 284 M^+^.

### Synthesis of N-(2-(3-phenylureido[thioueido])ethyl)-1,2,3,4-tetrahydroacridine-9-carboxamide derivatives 7a-f

To a solution of compound 3a-d (0.01 mol) in 50 mL absolute ethanol, 4-phenyl semicarbazide or 4-phenyl thiosemicarbazide (0.012 mol) was added. The reaction mixture was heated under reflux and after reaction completion (2 h as monitored by TLC), solvent was removed under reduced pressure and the residual mass was quenched with crushed ice water, left overnight. The formed solid was collected by filtration, washed thoroughly (water) and then recrystallized from proper solvent where compounds 7a-f were obtained.

### N-(2-(3-Phenylureido)ethyl)-1,2,3,4-tetrahydroacridine-9-carboxamide 7a

Yellow needles (MeOH), mp: 266–268 °C, yield 80%, (C_23_H_24_N_4_O_2_, MWt = 388.46), Calcd C: 71.04, H: 6.17, N: 14.41; Found: C: 69.68, H: 5.90, N: 14.02; IR (KBr, v, cm^−1^): 3255, 3210, 3176 (3NH), 3054 (CH_arom._), 2938 (CH_aliphatic_), 1678 (C=O), 1655 (C=O); ^1^H-NMR (400 MHz, δ*,* CDCl_3_): δ 1.26–1.30 (m, 4H, 2CH_2_), 1.77–1.82 (t, *J* = 8.0 Hz, 2H, CH_2_), 1.87–1.92 (t, *J* = 8.0 Hz, 2H, CH_2_), 3.28–3.36 (m, 4H, 2CH_2_), 7.32–7.44 (m, 4H, CH_arom_), 8.66 (s, 1H, NH exchangeable with D_2_O), 9.26 (s, 1H, NH exchangeable with D_2_O), 11.25 (s, 1H, NH exchangeable with D_2_O); ^13^C NMR (100 MHz, CDCl_3_): δ 21.03, 23.22, 28.34, 32.50, 41.88, 42.14, 121.66, 121.98, 122.25, 123.02, 123.55, 124.51, 127.89, 128.40, 131.51, 132.75, 135.21, 147.30, 158.65, 169.78, 188.65; MS (EI, m/z): 388 M^+^.

### 7-Bromo-N-(2-(3-phenylureido)ethyl)-1,2,3,4-tetrahydroacridine-9-carboxamide 7b

Brownish crystals (MeCN), mp: 230–23 °C, yield 86%, (C_23_H_23_BrN_4_O_2_, MWt = 467.35), Calcd C: 59.05, H: 4.92, N: 11.98, Br: 17.09; Found: C: 58.67, H: 4.56, N: 11.62, Br: 16.76; IR (KBr, v, cm^−1^): 3278, 3212, 3185 (3NH), 3050 (CH_arom._), 2942 (CH_aliphatic_), 1676 (C=O), 1658 (C=O); ^1^H-NMR (400 MHz, δ, CDCl_3_): δ 1.27–1.32 (m, 4H, 2CH_2_), 1.78–1.82 (t, *J* = 8.0 Hz, 2H, CH_2_), 1.88–1.92 (t, *J* = 8.0 Hz, 2H, CH_2_), 3.26–3.36 (m, 4H, 2CH_2_), 7.38–7.45 (m, 3H, CH_arom_), 8.67 (s, 1H, NH exchangeable with D_2_O), 9.22 (s, 1H, NH exchangeable with D_2_O), 11.34 (s, 1H, NH exchangeable with D_2_O); ^13^C NMR (100 MHz, CDCl_3_): δ 21.25, 23.43, 28.56, 32.76, 41.89, 42.23, 121.71, 121.90, 122.33, 123.21, 123.32, 124.42, 127.61, 128.68, 131.31, 132.67, 135.05, 147.62, 158.73, 169.44, 188.63; MS (EI, m/z): 469 M^+2^.

### 7-Chloro-N-(2-(3-phenylureido)ethyl)-1,2,3,4-tetrahydroacridine-9-carboxamide 7c

Brownish crystals (MeCN), mp: 230–23 °C, yield 86%, (C_23_H_23_ClN_4_O_2_, MWt = 422.15), Calcd C: 65.32, H: 5.48, Cl 8.38, N: 13.25; Found: C: 65.02, H: 5.14, Cl 8.01, N: 10.87; IR (KBr, v, cm^−1^): 3278, 3212, 3185 (3NH), 3050 (CH_arom._), 2942 (CH_aliphatic_), 1676 (C=O), 1658 (C=O); ^1^H-NMR (400 MHz, δ*,* CDCl_3_): δ 1.27–1.32 (m, 4H, 2CH_2_), 1.78–1.82 (t, *J* = 8.0 Hz, 2H, CH_2_), 1.88–1.92 (t, *J* = 8.0 Hz, 2H, CH_2_), 3.26–3.36 (m, 4H, 2CH_2_), 7.38–7.45 (m, 3H, CH_arom_), 8.67 (s, 1H, NH exchangeable with D_2_O), 9.22 (s, 1H, NH exchangeable with D_2_O), 11.34 (s, 1H, NH exchangeable with D_2_O); ^13^C NMR (100 MHz, CDCl_3_): δ 21.25, 23.43, 28.56, 32.76, 41.89, 42.23, 121.71, 121.90, 122.33, 123.21, 123.32, 124.42, 127.61, 128.68, 131.31, 132.67, 135.05, 147.62, 158.73, 169.44, 188.63; MS (EI, m/z): 424 M^+2^.

### 7-Methyl-N-(2-(3-phenylureido)ethyl)-1,2,3,4-tetrahydroacridine-9-carboxamide 7d

Brownish crystals (MeCN), mp: 230–23 °C, yield 86%, (C_24_H_26_N_4_O_2_, MWt = 402.21), Calcd C: 71.62, H: 6.51, N: 13.92; Found: C: 71.40, H: 6.23, N: 13.67; IR (KBr, v, cm^−1^): 3278, 3212, 3185 (3NH), 3050 (CH_arom._), 2942 (CH_aliphatic_), 1676 (C=O), 1658 (C=O); ^1^H-NMR (400 MHz, δ, CDCl_3_): δ 1.27–1.32 (m, 4H, 2CH_2_), 1.78–1.82 (t, *J* = 8.0 Hz, 2H, CH_2_), 1.88–1.92 (t, *J* = 8.0 Hz, 2H, CH_2_), 2.12 (s, 3H, CH_3_), 3.26–3.36 (m, 4H, 2CH_2_), 7.38–7.45 (m, 3H, CH_arom_), 8.67 (s, 1H, NH exchangeable with D_2_O), 9.22 (s, 1H, NH exchangeable with D_2_O), 11.34 (s, 1H, NH exchangeable with D_2_O); ^13^C NMR (100 MHz, CDCl_3_): δ 21.25, 23.43, 28.56, 32.76, 41.89, 42.23, 44.68, 121.71, 121.90, 122.33, 123.21, 123.32, 124.42, 127.61, 128.68, 131.31, 132.67, 135.05, 147.62, 158.73, 169.44, 188.63; MS (EI, m/z): 402 M^+^.

### N-(2-(3-Phenylthioureido)ethyl)-1,2,3,4-tetrahydroacridine-9-carboxamide 7e

Pale yellow crystals (dioxane), mp: 186–188 °C, yield 72%, (C_23_H_24_N_4_OS, MWt = 404.52), Calcd C: 68.22, H: 5.93, N: 13.84, S: 7.91; Found: C: 67.87, H: 5.90, N: 13.56, S: 7.50; IR (KBr, v, cm^−1^): 3260, 3217, 3176 (3NH), 3066 (CH_arom._), 2940 (CH_aliphatic_), 1676 (C=O), 1657 (C=O), 1325 (C=S); ^1^H-NMR (400 MHz, δ, CDCl_3_): δ 1.26–1.30 (m, 4H, 2CH_2_), 1.77–1.82 (t, *J* = 8.0 Hz, 2H, CH_2_), 1.87–1.92 (t, *J* = 8.0 Hz, 2H, CH_2_), 3.28–3.36 (m, 4H, 2CH_2_), 7.32–7.44 (m, 4H, CH_arom_), 8.66 (s, 1H, NH exchangeable with D_2_O), 9.26 (s, 1H, NH exchangeable with D_2_O), 11.25 (s, 1H, NH exchangeable with D_2_O); ^13^C NMR (100 MHz, CDCl_3_): δ 21.33, 23.41, 28.42, 32.56, 41.81, 42.22, 121.34, 121.90, 122.21, 123.22, 123.50, 124.67, 127.87, 128.55, 131.67, 132.88, 135.32, 147.42, 158.65, 181.08, 188.45; MS (EI, m/z): 404 M^+^.

### 7-Bromo-N-(2-(3-phenylthioureido)ethyl)-1,2,3,4-tetrahydroacridine-9-carboxamide 7f

Light brown crystals (CH_3_CN), mp: 166–168 °C, yield 76%, (C_23_H_23_BrN_4_OS, MWt = 483.42), Calcd C: 57.09, H: 4.75, N: 11.58, S: 6.61, Br: 16.52; Found: C: 56.76, H: 4.43, N: 11.42, S: 6.35, Br: 16.08; IR (KBr, v, cm^−1^): 3266, 3216, 3170 (3NH), 3065 (CH_arom._), 2944 (CH_aliphatic_), 1677 (C=O), 1658 (C=O), 1320 (C=S); ^1^H-NMR (400 MHz, δ, CDCl_3_): δ 1.27–1.31 (m, 4H, 2CH_2_), 1.78–1.82 (t, *J* = 8.0 Hz, 2H, CH_2_), 1.88–1.92 (t, *J* = 8.0 Hz, 2H, CH_2_), 3.28–3.35 (m, 4H, 2CH_2_), 7.32–7.44 (m, 3H, CH_arom_), 8.63 (s, 1H, NH exchangeable with D_2_O), 9.25 (s, 1H, NH exchangeable with D_2_O), 11.27 (s, 1H, NH exchangeable with D_2_O); ^13^C NMR (100 MHz, CDCl_3_): δ 21.30, 23.43, 28.40, 32.56, 41.87, 42.28, 121.37, 121.93, 122.24, 123.23, 123.54, 124.66, 127.84, 128.50, 131.63, 132.80, 135.35, 147.47, 158.60, 181.23, 188.87; MS (EI, m/z): 485 M^+2^.

### 7-Chloro-N-(2-(3-phenylthioureido)ethyl)-1,2,3,4-tetrahydroacridine-9-carboxamide 7 g

Light brown crystals (CH_3_CN), mp: 181–183 °C, yield 75%, (C_23_H_23_ClN_4_OS, MWt = 438.97), Calcd C: 62.93, H: 5.28, Cl 8.08, N: 12.76, S: 7.30; Found: C: 62.55, H: 5.01, N: 12.66, S: 7.09; IR (KBr, v*,* cm^−1^): 3266, 3216, 3170 (3NH), 3065 (CH_arom._), 2944 (CH_aliphatic_), 1677 (C=O), 1658 (C=O), 1320 (C=S); ^1^H-NMR (400 MHz, δ*,* CDCl_3_): δ 1.27–1.31 (m, 4H, 2CH_2_), 1.78–1.82 (t, *J* = 8.0 Hz, 2H, CH_2_), 1.88–1.92 (t, *J* = 8.0 Hz, 2H, CH_2_), 3.28–3.35 (m, 4H, 2CH_2_), 7.32–7.44 (m, 3H, CH_arom_), 8.63 (s, 1H, NH exchangeable with D_2_O), 9.25 (s, 1H, NH exchangeable with D_2_O), 11.27 (s, 1H, NH exchangeable with D_2_O); ^13^C NMR (100 MHz, CDCl_3_): δ 21.30, 23.43, 28.40, 32.56, 41.87, 42.28, 121.37, 121.93, 122.24, 123.23, 123.54, 124.66, 127.84, 128.50, 131.63, 132.80, 135.35, 147.47, 158.60, 181.23, 188.87; MS (EI, m/z): 440 M^+2^.

### 7-Methyl-N-(2-(3-phenylthioureido)ethyl)-1,2,3,4-tetrahydroacridine-9-carboxamide 7 h

Light brown crystals (CH_3_CN), mp: 202–205 °C, yield 75%, (C_24_H_26_N_4_OS, MWt = 418.18), Calcd C: 68.87, H: 6.26, N: 13.39, S: 7.66; Found: C: 68.54, H: 6.01, N: 13.12, S: 7.39; IR (KBr, v*,* cm^−1^): 3266, 3216, 3170 (3NH), 3065 (CH_arom._), 2944 (CH_aliphatic_), 1677 (C=O), 1658 (C=O), 1320 (C=S); ^1^H-NMR (400 MHz, δ, CDCl_3_): δ 1.27–1.31 (m, 4H, 2CH_2_), 1.78–1.82 (t, *J* = 8.0 Hz, 2H, CH_2_), 1.88–1.92 (t, *J* = 8.0 Hz, 2H, CH_2_), 2.11 (s, 3H, CH_3_), 3.28–3.35 (m, 4H, 2CH_2_), 7.32–7.44 (m, 3H, CH_arom_), 8.63 (s, 1H, NH exchangeable with D_2_O), 9.25 (s, 1H, NH exchangeable with D_2_O), 11.27 (s, 1H, NH exchangeable with D_2_O); ^13^C NMR (100 MHz, CDCl_3_): δ 21.30, 23.43, 28.40, 32.56, 41.87, 42.28, 121.37, 121.93, 122.24, 123.23, 123.54, 124.66, 127.84, 128.50, 131.63, 132.80, 135.35, 147.47, 158.60, 181.23, 188.87; MS (EI, m/z): 418 M^+^.

### Synthesis of 2-(1,2,3,4-tetrahydroacridine-9-carbonyl)hydrazinecarbothioamide derivatives 8a-d

A mixture of compound **3a-d** (0.01 mol) in 50 mL absolute ethanol was treated with thiosemicarbazide (1.1 g, 0.012 mol). The reaction mixture was heated under reflux for 3 h, after reaction completion (monitored by TLC), solvent was removed under reduced pressure and the crushed-ice water was added. The formed solid was collected by filtration, washed (water) and recrystallized from acetonitrile into compounds **8a-d**.

### 2-(1,2,3,4-Tetrahydroacridine-9-carbonyl)hydrazinecarbothioamide 8a

Pale yellow needles mp: 174–176 °C, yield 82%, (C_15_H_16_N_4_OS, MWt = 300.38), Calcd C: 59.92, H: 5.32, N: 18.64, S: 10.65; Found: C: 59.58, H: 5.01, N: 18.34, S: 10.33; IR (KBr, v*,* cm^−1^): 3318, 3232, 3170 (NH_2_, 2NH), 3058 (CH_arom._), 2943 (CH_aliphatic_), 1672 (C=O), 1452 (C=S); ^1^H-NMR (400 MHz, δ*,* CDCl_3_): δ 1.27–1.31 (m, 4H, 2CH_2_), 1.78–1.82 (t, *J* = 8.0 Hz, 2H, CH_2_), 1.88–1.92 (t, *J* = 8.0 Hz, 2H, CH_2_), 7.32–7.44 (m, 4H, CH_arom_), 8.12 (s, 2H, NH_2_ exchangeable with D_2_O), 9.23 (s, 1H, NH exchangeable with D_2_O), 11.20 (s, 1H, NH exchangeable with D_2_O); ^13^C NMR (100 MHz, CDCl_3_): δ 21.19, 23.28, 28.74, 32.51, 121.62, 122.28, 123.50, 124.54, 127.81, 128.46, 135.27, 147.33, 158.61, 169.67, 180.60; MS (EI, m/z): 300 M^+^.

### 2-(7-Bromo-1,2,3,4-tetrahydroacridine-9-carbonyl)hydrazinecarbothioamide 8b

Brownish crystals mp: 158–160 °C, yield 86%, (C_15_H_15_BrN_4_OS, MWt = 379.27), Calcd C: 47.45, H: 3.95, N: 14.76, S: 8.43, Br: 21.06; Found: C: 47.07, H: 4.01, N: 14.39, S: 8.20, Br: 20.77; IR (KBr, v, cm^−1^): 3323, 3230, 3178 (NH_2_, 2NH), 3045 (CH_arom._), 2956 (CH_aliphatic_), 1678 (C=O), 1458 (C=S); ^1^H-NMR (400 MHz, δ, CDCl_3_): δ 1.26–1.31 (m, 4H, 2CH_2_), 1.77–1.82 (t, *J* = 8.0 Hz, 2H, CH_2_), 1.88–1.92 (t, *J* = 8.0 Hz, 2H, CH_2_), 7.32–7.44 (m, 3H, CH_arom_), 8.18 (s, 2H, NH_2_ exchangeable with D_2_O), 9.25 (s, 1H, NH exchangeable with D_2_O), 11.28 (s, 1H, NH exchangeable with D_2_O); ^13^C NMR (100 MHz, CDCl_3_): δ 21.22, 23.23, 28.77, 32.55, 121.66, 122.23, 123.51, 124.57, 127.85, 128.41, 135.20, 147.88, 158.66, 169.87, 180.77; MS (EI, m/z): 381 M^+2^.

### 2-(7-Chloro-1,2,3,4-tetrahydroacridine-9-carbonyl)hydrazinecarbothioamide 8c

Yellow crystals, mp: 162–164 °C, yield 80%, (C_17_H_19_N_5_O_2_S, MWt=357.43), Calcd C: 57.07, H: 5.13, N: 19.58, S: 8.95; Found: C: 56.76, H: 4.87, N: 19.26, S: 8.65; IR (KBr, v*,* cm^−1^): 3318, 3268, 3215, 3170 (2NH, NH_2_), 3054 (CH_arom._), 2948 (CH_aliphatic_), 1677 (C=O), 1658 (C=O), 1350 (C=S); ^1^H-NMR (400 MHz, δ, CDCl_3_): δ 1.28–1.32 (m, 4H, 2CH_2_), 1.78–1.82 (t, *J* = 8.0 Hz, 2H, CH_2_), 1.88–1.92 (t, *J* = 8.0 Hz, 2H, CH_2_), 2.22 (s, 3H, CH_3_), 6.62 (br, 2H, NH_2_ exchangeable with D_2_O), 7.36–7.45 (m, 3H, CH_arom_), 9.22 (s, 1H, NH exchangeable with D_2_O), 11.23 (s, 1H, NH exchangeable with D_2_O); ^13^C NMR (100 MHz, CDCl_3_): δ 21.30, 23.44, 28.35, 32.53, 42.28, 121.61, 122.26, 123.15, 127.22, 128.47, 131.50, 132.73, 135.24, 147.31, 169.61, 185.12, 188.60; MS (EI, m/z): 359 M^+^.

### 2-(7-Methyl-1,2,3,4-tetrahydroacridine-9-carbonyl)hydrazinecarbothioamide 8d

Pale yellow crystals mp: 173–175 °C, yield 84%, (C_16_H_18_N_4_OS, MWt = 314.12), Calcd C: 61.12, H: 5.77, N: 17.82, S: 10.20; Found: C: 60.89, H: 5.50, N: 17.61, S: 10.02; IR (KBr, v*,* cm^−1^): 3323, 3230, 3178 (NH_2_, 2NH), 3045 (CH_arom._), 2956 (CH_aliphatic_), 1678 (C=O), 1458 (C=S); ^1^H-NMR (400 MHz, δ, CDCl_3_): δ 1.26–1.31 (m, 4H, 2CH_2_), 1.77–1.82 (t, *J* = 8.0 Hz, 2H, CH_2_), 1.88–1.92 (t, *J* = 8.0 Hz, 2H, CH_2_), 2.12 (s, 3H, CH_3_), 7.32–7.44 (m, 3H, CH_arom_), 8.18 (s, 2H, NH_2_ exchangeable with D_2_O), 9.25 (s, 1H, NH exchangeable with D_2_O), 11.28 (s, 1H, NH exchangeable with D_2_O); ^13^C NMR (100 MHz, CDCl_3_): δ 21.22, 23.23, 28.77, 32.55, 121.66, 122.23, 123.51, 124.57, 127.85, 128.41, 135.20, 147.88, 158.66, 169.87, 180.77; MS (EI, m/z): 314 M^+^.

## Conclusion

This study highlights the successful application of structure-based design principles in the development of tetrahydroacridine derivatives as potential antidiabetic agents. By incorporating strategic substitutions and molecular features into the THA core, we were able to modulate both physicochemical properties and biological performance. The structural diversity introduced into the acridine framework allowed for fine-tuning of key interactions with diabetic targets, as confirmed by molecular docking analyses against DPP-IV, SGLT1, and GLUT2. The consistency between binding site interactions, *in-vitro* glucose diffusion inhibition, and *in-vivo* antihyperglycemic effects demonstrates the value of rational design rooted in scaffold modification. Compounds such as **4a, 4b, and 5a** exemplify how targeted changes at the molecular level can yield derivatives with enhanced biological profiles and drug-like characteristics. These results reinforce the importance of heterocyclic scaffold optimization in drug discovery and suggest that further structure–activity relationship (SAR) exploration and target-specific derivatization could lead to highly selective and potent antidiabetic candidates within this chemotype.

## Supplementary Information

Below is the link to the electronic supplementary material.

Supplementary Material 1

Supplementary Material 2

Supplementary Material 3

## Data Availability

All information generated or examined during this inquiry is contained in this published article and it’s supporting information files.

## References

[1] Bouffier, L., Demeunynck, M., Milet, A. & Dumy, P. Reactivity of pyrido[4,3,2-kl]acridines: Regioselective formation of 6-substituted derivatives. *J. Org. Chem.* **69**, 8144–8147. 10.1021/jo0487855 (2004). 15527309 10.1021/jo0487855

[2] Chiron, J. & Galy, J. P. Reactivity of the acridine ring: A review. *Synthesis* **3**, 313–325. 10.1055/s-2003-44379 (2004).

[3] Chen, H. *Huaxue Yanjiu Yu Yingyong*, 12, 164‐168 (2000).

[4] Sivan, S., Tuchman, S. & Lotan, N. A biochemical logic gate using an enzyme and its inhibitor Part II: The logic gate. *Biosystems* **70**(1), 21–33 (2003). 12753934 10.1016/s0303-2647(03)00039-x

[5] Flock, S. et al. Interaction of two peptide-acridine conjugates containing the SPKK peptide motif with DNA and chromatin. *J. Biomol. Struct. Dyn.* **11**(4), 881–900 (1994). 8204221 10.1080/07391102.1994.10508039

[6] Gooch, B. D. & Beal, P. A. Recognition of duplex RNA by helix-threading peptides. *J. Am. Chem. Soc.* **126**(34), 10603–10610 (2004). 15327318 10.1021/ja047818v

[7] Stefanska, B. et al. 2, 7-Dihydro-3H-pyridazino [5, 4, 3-kl] acridin-3-one derivatives, novel type of cytotoxic agents active on multidrug-resistant cell lines. Synthesis and biological evaluation. *Bioorg. Med. Chem.* **13**, 1969–1975. 10.1016/j.bmc.2005.01.023 (2005). 15727851 10.1016/j.bmc.2005.01.023

[8] Demeunynck, M. Antitumour acridines. *Expert Opin. Ther. Patents* **14**, 55–70. 10.1517/13543776.14.1.55 (2004).

[9] Abdel‐Halim, A. M.; Tawfik, A. M.; Ibrahim, S. S. & El‐Kazak, A. M. *Indian J. Heterocyclic. Chem.* 3, 165‐170 (1994)

[10] Chen, Y. L., Lu, C. M., Chen, I. L., Tsao, L. T. & Wang, J. P. Synthesis and antiinflammatory evaluation of 9-anilinoacridine and 9-phenoxyacridine derivatives. *J. Med. Chem.* **45**(21), 4689–4694 (2002). 12361395 10.1021/jm020102v

[11] Chen, Y. L. et al. Synthesis and anti-inflammatory evaluation of 9-phenoxyacridine and 4-phenoxyfuro [2, 3-b] quinoline derivatives Part 2. *Bioorg. & Med. Chem.* **11**(18), 3921–3927 (2003). 12927852 10.1016/s0968-0896(03)00439-5

[12] Skotnicki, J. S. & Gilman, S. C. US 851536. *Chem. Abstr.* **112**, 118672 (1990).

[13] Sánchez, I., Reches, R., Caignard, D. H., Renard, P. & Pujol, M. D. Synthesis and biological evaluation of modified acridines: The effect of N-and O-substituent in the nitrogenated ring on antitumor activity. *Eur. J. Med. Chem.* **41**(3), 340–352 (2006). 16413635 10.1016/j.ejmech.2005.11.006

[14] Yang, P., Yang, Q., Qian, X., Tong, L. & Li, X. *J. Photochem. Photobiol. B: Biol.* **84**(3), 221–226. 10.1016/j.jphotobiol.2006.03.005 (2006). 10.1016/j.jphotobiol.2006.03.005 16714120

[15] Julien, C. et al. Synthesis and antileishmanial activities of 4, 5-di-substituted acridines as compared to their 4-mono-substituted homologues. *Bioorg. Med. Chem.* **13**(19), 5560–5568 (2005). 16081295 10.1016/j.bmc.2005.06.045

[16] Cremieux, A. et al. Antimicrobial activity of 9-oxo and 9-thio acridines: Correlation with intercalation into DNA and effects on macromolecular biosynthesis. *Res. Microbiol.* **146**(1), 73–83 (1995). 7538688 10.1016/0923-2508(96)80272-5

[17] Helal, M. H. et al. Rational design, synthesis, and *in silico* evaluation of novel pyridine-based heterocyclic compounds as multifunctional antidiabetic agents: Molecular docking and ADMET profiling. *J. Mol. Struct.* **1349**, 143703. 10.1016/j.molstruc.2025.143703 (2026).

[18] Aktar, B. S. K. et al. Discovery of novel pyrrolidine-based chalcones as dual inhibitors of α-amylase and α-glucosidase: Synthesis Molecular Docking, ADMET Profiles, and Pharmacological Screening. *ACS Omega*10.1021/acsomega.4c10095 (2025). 10.1021/acsomega.4c10095 PMC11904715 40092796

[19] Recanatini, M., Cavalli, A. & Bellut, F. SAR of 9-amino-1, 2, 3, 4-tetrahydroacridine-based acetylcholineestrasen inhibitors, synthesis, enzyme inhibitory activity, QSAR and structure-based CoMFA of tacrine analogues. *J Med Chem.*10.1021/jm990971t (2000). 10821713 10.1021/jm990971t

[20] Bindra, J. S., Rastogi, S. N., Patansik, G. K. & Qnand, N. Synthesis, pharmacological activities and physicochemical properties of 4-substituted amino/N4-arylpipeperazinylaminocarbonyl-2,3-polymethylenequinoline. *Indian J. Chem.*10.1007/s11172-023-3844-8 (1987).

[21] Dinesen, J., Jacobsen, J. P., Hanzen, F., Pedersen, E. & Eggere, H. DNA intercalating properties of tetrahyddroacridines. *J. Med. Chem.* **33**, 93–97. 10.1021/jm00163a015 (1990). 2296037 10.1021/jm00163a015

[22] Rosini, M. et al. Prazosin-related compounds. Effect of transforming the piperazinyl quinazoline moiety into aminomethyl tetrahydroacridine system on the affinity for α1-adrenoreceptors. *J. Med. Chem.* **46**, 4895–4903. 10.1021/jm030952q (2003). 14584940 10.1021/jm030952q

[23] Rosini, M, Andrisano, V, Bartolini, M & Melchiorre, C. Tetrahydroacridine and dithiolane derivatives as a treatment of Alzheimer’s disease. US Patent 7307083 B2; 11 December **2007**.

[24] Carlier, P. R., Du, D. M., Han, Y., Liu, J. & Pang, Y. P. Potent easily synthesized huperzine A-tacrine hybrid acetylcholinesterase inhibitors. *Bioorg. Med. Chem. Lett.* **9**, 2335–2338 (1999). 10476864 10.1016/s0960-894x(99)00396-0

[25] Nguyen, T., Yang, T. M. & Go, M. L. Functionalized acridin-9-yl-phenylamines protected neuronal HT22 cell from glutamate-induced cell death by reducing intracellular levels of free radical species. *Bioorg. Med. Chem. Lett.* **24**, 1830–1838. 10.1016/j.bmcl.2014.02.006 (2014). 24602904 10.1016/j.bmcl.2014.02.006

[26] Mounir, M., Asmaa, K., Marwa, F. & Ahmed, E. S. Synthesis, characterization and antibacterial activity of some novel spiro[naphtho[1,2-e][1,3]oxazine-3,4’-pyran] derivatives. *J. Pharm. Appl. Chem.* **7**(3), 1–10 (2021).

[27] Bhutani, R. et al. Synthesis, Molecular modelling studies and ADME prediction of benzothiazole clubbed oxadiazole-Mannich bases, and evaluation of their Anti-diabetic activity through *in-vivo* model. *Bioorganic Chem.* **77**, 6–15. 10.1016/j.bioorg.2017.12.037 (2018). 10.1016/j.bioorg.2017.12.037 29316509

[28] Cho, N. H. et al. IDF Diabetes Atlas: Global estimates of diabetes prevalence for 2017 and projections for 2045. *Diabetes Res. Clin. Pract.* **138**, 271–281. 10.1016/j.diabres.2018.02.023 (2018). 29496507 10.1016/j.diabres.2018.02.023

[29] International Diabetes Federation. IDF Diabetes Atlas, 11th edn. (International Diabetes Federation, Brussels, Belgium, 2025). Available at: https://diabetesatlas.org

[30] World Health Organization. Diabetes–Fact sheet. WHO, November 2024. Available at: https://www.who.int/news-room/fact-sheets/detail/diabetes

[31] Diamond, J. The double puzzle of diabetes. *Nature* **423**, 599–602. 10.1038/423599a (2003). 12789325 10.1038/423599a

[32] King, H., Aubert, R. E. & Herman, W. H. Global burden of diabetes, 1995–2025: Prevalence, numerical estimates, and projections. *Diabetes Care* **21**, 1414–1431. 10.2337/DIACARE.21.9.1414 (1998). 9727886 10.2337/diacare.21.9.1414

[33] Thévenod, F. Pathophysiology of diabetes mellitus type 2: Roles of obesity, insulin resistance and -cell dysfunction. *Front Diabetes Basel Karger* **19**, 1–18. 10.1159/000152019 (2008).

[34] Mohamed, M. A. et al. Spiro heterocycles bearing piperidine moiety as potential scaffold for antileishmanial activity: Synthesis, biological evaluation, and in silico studies. *J. Enzyme Inhib. Med. Chem.* **38**(1), 330–342 (2023). 36444862 10.1080/14756366.2022.2150763 PMC11003478

[35] Abdelmonsef, A. H., El-Saghier, A. M. & Kadry, A. M. Ultrasound-assisted green synthesis of triazole-based azomethine/thiazolidin-4-one hybrid inhibitors for cancer therapy through targeting dysregulation signatures of some Rab proteins. *Green Chem. Lett. Rev.* **16**(1), 2150394 (2023).

[36] El-Saghier A. M. M., Mohamed M. A, A., Abdalla O. A. & Kadry A. M., Utility of amino acid coupled 1,2,4-triazoles in organic synthesis: Synthesis of some new antileishmainal agents. *Bull. Chem. Soc. Ethiopia* **32** (3), 559–570. 10.4314/bcse.v32i3.15 (2018).

[37] Abd Allah, O. A., El-Saghier, A. M. & Kadry, A. M. Synthesis, structural stability calculation, and antibacterial evaluation of novel 3, 5-diphenylcyclohex-2-en-1-one derivatives. *Synth. Commun.* **45**(8), 944–957 (2015).

[38] Abd Allah O. A., El-Saghier A. M., Kadry A. M. & Seleem A. A. Synthesis and evaluation of some novel curcumin derivatives as anti-inflammatory agents. *Int. J. Pharm. Sci. Rev. Res.* **32** (1), 87–92 (2015).

[39] El-Saghier, A. M., Enaili, S. S., Abdou, A., Hamed, A. M. & Kadry, A. M. Synthesis, docking and biological evaluation of purine-5-N-isosteresas anti-inflammatory agents. *RSC Adv.* **14**(25), 17785–17800 (2024). 38832248 10.1039/d4ra02970d PMC11146149

[40] Ivachtchenko, A. V. & Kobak, V. V. Il’yin AP, Trifilenkov AS, Busel AA. *J. Comb. Chem.* **5**, 645 (2003). 10.1021/cc0100316 12217013

[41] H. Beyzaei, A. Beygi, R. Aryan, *Iran. J. Chem. Chem. Eng.*, 35(4) 31-37 (2016)

[42] Malik, C. P. & Singh, M. B. *Plant Enzymology and Histoenzymology* 278 (Kalyani Publishers, 1980).

[43] Krishnaveni, S., Balasubramanian, T. & Sadasivam, S. Sugar distribution in sweet stalk sorghum. *Food Chem.* **15**(3), 229–232 (1984).

[44] Gohar, N. A. et al. Fluorinated indeno-quinoxaline bearing thiazole moieties as hypoglycaemic agents targeting α-amylase, and α-glucosidase: synthesis, molecular docking, and ADMET studies. *J. Enzyme Inhib. Med. Chem.* **39**(1), 2367128. 10.1080/14756366.2024.2367128 (2024). 38913598 10.1080/14756366.2024.2367128 PMC467095

[45] Beyzaei H., Beygi A., Aryan R., *Iran. J. Chem. Chem. Eng.*, 35(4), 31. 10.30492/IJCCE.2016.23554 (2016)

[46] Mohamed, M. A., Abd Allah, O. A., Bekhit, A. A., Kadry, A. M. & El-Saghier, A. M. Synthesis and antidiabetic activity of novel triazole derivatives containing amino acids. *J. Heterocycl. Chem.* **57**(6), 2365–2378 (2020).

[47] Mowla, A., Alauddin, M., Rahman, M. A. & Ahmed, K. Antihyperglycemic efect of Trigonella foenum-graecum (fenugreek) seed extract in alloxan-induced diabetic rats and its use in diabetes mellitus: A brief qualitative phytochemical and acute toxicity test on the extract. *Afr. J. Tradit. Complement. Altern. Med.* **6**(3), 255–261 (2009). 20448850 10.4314/ajtcam.v6i3.57165 PMC2816457

[48] Singh, F. V. et al. 5,6-Diarylanthranilo-1,3-dinitriles as a new class of antihyperglycemic agents. *Bioorg. Med. Chem. Lett.* **19**(8), 2158–2161. 10.1016/j.bmcl.2009.02.118 (2009). 19303291 10.1016/j.bmcl.2009.02.118

[49] D’Andrea, G. et al. Protein glycans alteration and a diferentdistribution of some enzymatic activities involvedin the glycan processing are found in AZT-treated K562 cells. *Mol. Cell. Biochem.* **22**(1–2), 45–51. 10.1023/A:1025561009412 (2003). 10.1023/a:1025561009412 14577575

[50] Kim, N. N. et al. Streptozotocin-induced diabetes in the rat is associated with changes in vaginal hemodynamics, morphology and biochemicalmarkers. *BMC Physiol.* **6**, 14 (2006). 10.1186/1472-6793-6-4 PMC1481539 16734901

[51] Reitman, S. & Frankel, S. A. A colorimetric method for the determination of serumglutamic oxalacetic and glutamic pyruvic transaminases. *Am. J.Clin. Pathol.* **28**(1), 56–63. 10.1093/ajcp/28.1.56 (1957). 13458125 10.1093/ajcp/28.1.56

[52] Henry, R. J. *Clinical Chemistry: Principles and Techniques* (Harper & Row, 1964).

[53] Ferreira, L. L. & Andricopulo, A. D. ADMET modeling approaches in drug discovery. *Drug Discov. Today* **24**(5), 1157–1165 (2019). 30890362 10.1016/j.drudis.2019.03.015

[54] El-Saghier A. M., Abdul-Baset A., El-Hady O. M., Abd El-Raheem W. M. & Kadry A. M. Synthesis, docking and characterization of some novel 5-(S-alkyl)-1.3.4-thiadiazole-2-carboxamide derivatives as anti-inflammatory and antibacterial agents. *BMC Chem.* **18** (1), 138. 10.1186/s13065-024-01237-9 (2024). 10.1186/s13065-024-01237-9 PMC11282722 39068479

[55] Kadry, A. M., Soliman, A. G. & Abbas, M. M. Computational prediction and QSAR-based design of novel curcumin derivatives: Enhancing insulin receptor binding and pharmacokinetic properties for improving therapeutic efficacy. *Biol. Biomed. J.* **3**(2), 73–78 (2025).

[56] Ali, O. A., Ragab, A., Ammar, Y. A. & Abusaif, M. S. Discovery of new thiazolidin-4-one and thiazole nucleus incorporation sulfaguanidine scaffold as new class of antimicrobial agents: Design, synthesis, in silico ADMET, and docking simulation. *J. Mol. Struct.* **5**(1334), 141879 (2025).

[57] Abusaif, M. S. et al. Exploring novel of 1, 2, 4-triazolo [4, 3-a] quinoxaline sulfonamide regioisomers as anti-diabetic and anti-Alzheimer agents with in-silico molecular docking simulation. *Sci. Rep.* **15**(1), 19409 (2025). 40461519 10.1038/s41598-025-03139-9 PMC12134304

