# Supplementary material for: Synthesis and antidiabetic evaluation of novel Tetrahydroacridine derivatives with computational insights
Source: Sci Rep. 2025 Dec 13;15:43837. doi: 10.1038/s41598-025-28702-2 (PMC12706072; doi:10.1038/s41598-025-28702-2)
Supplement: Supplementary file 2 — Supplementary Material 2 [file 41598_2025_28702_MOESM2_ESM.docx]

**Multidimensional Evaluation of Novel Tetrahydroacridine Derivatives: Design, Synthesis, Molecular Docking, In Vitro and In Vivo Antidiabetic Studies with ADMET Profiling**

Asmaa M. Kadry^a*^, Wafaa A. Abdellah^b^ and Mounir A. A. Mohamed^a^

*^a^ Chemistry Department, Faculty of Science, Sohag University, 82524 Sohag, Egypt.*

*^b^ Medical Pharmacology department, Faculty of medicine, Sohag University, Egypt.*

^*^Corresponding Author: [asmaa.kadry@science.sohag.edu.eg](mailto:asmaa.kadry@science.sohag.edu.eg)

**Experimental:**

All commercially available reagents were purchased from Merck, Aldrich and Fluka and were used without further purification. All reactions were monitored by thin layer chromatography (TLC) using precoated plates of silica gel G/UV-254 of 0.25 mm thickness (Merck 60F254) using UV light (254 nm/365 nm) for visualization. Melting points were detected with a Kofler melting points apparatus and uncorrected. Infrared spectra were recorded with a FT-IR-ALPHBROKER-Platinum-ATR spectrometer and are given as cm^-1^ using the attenuated total reflection (ATR) method. ^1^H NMR and ^13^C NMR spectra for all compounds were recorded in DMSO-d_6_ on a Bruker Bio Spin AG spectrometer at 400 MHz and 100 MHz, respectively. For ^1^H NMR, chemical shifts (δ) were given in parts per million (ppm) with reference to tetramethylsilane (TMS) as an internal standard (δ=0); coupling constants (J) were given in hertz (Hz) and data are reported as follows: chemical shift, integration, multiplicity (s = singlet, d = doublet, t = triplet, q = quartet, m = multiplet). For ^13^CMR, TMS (δ=0) or DMSO (δ=39.51) was used as internal standard and spectra were obtained with complete proton decoupling. Elemental analyses were obtained on a Perkin-Elmer CHN-analyzer model.

***General procedure for the synthesis of 1,2,3,4-tetrahydroacridine-9-carboxylic acid derivatives 2a-d:***

A mixture of isatin derivative **1a-d** (0.001 mol) in ethanol (5 mL) and potassium hydroxide (0.25 g in water 5 mL) was stirred at room temperature for 15–30 min. The mixture was then acidiﬁed to pH 2–3 with concentrated hydrochloric acid, and then cyclohexanone (0.103 mL, 0.001 mol) and CuSO_4_.7H_2_O (0.025 g, 0.1 mmol) were added. The resulting mixture was stirred until precipitate appeared. The reaction progress was monitored by TLC (CHCl_3_/MeOH 9:1). After the starting material had vanished, the precipitate was ﬁltered off, washed with water, and recrystallized to afford the pure product 1,2,3,4-tetrahydrocridine-9-carboxylic acid derivative **2a-d**.

***1,2,3,4-Tetrahydroacridine-9-carboxylic acid 2a:***

|  | Pale yellow needles, mp: 282-285 °C [28], (0.178 g, 78%); (C_14_H_13_NO_2_ M.wt=227.12); Calcd C:74.0, H: 5.72, N: 6.16; Found C: 73.64, H: 5.44, N: 5.89; IR (KBr, ν, cm^-1^): 3467-3318 (broad band OH), 3045 (CH_arom._), 2933 (CH_aliphatic_), 1642 (C=O); ^1^H-NMR (400 MHz, δ, CDCl_3_): δ 1.22-1.36 (m, 4H, 2CH_2_), 1.82-1.77 (t, *J* = 8.0 Hz, 2H, CH_2_), 1.93-1.87 (t, *J* = 8.0 Hz, 2H, CH_2_), 7.34-7.60 (m, 4H, CH_arom_), 12.24 (br, 1H, COOH); ^13^C NMR (100 MHz, CDCl_3_): δ 22.90, 23.20, 29.10, 33.55, 125.50, 126.67, 127.32, 128.45, 130.95, 132.34, 135.11, 146.67, 151.35, 168.65; MS (EI, m/z): 227 M^+^. |
| --- | --- |

***7-Bromo-1,2,3,4-tetrahydroacridine-9-carboxylic acid 2b:***

|  | Pale yellow crystals, mp: 240-42 °C, (0.25 g, 82%); (C_14_H_12_BrNO_2_, MWt=306.15); Calcd C:54.90, H: 3.92, N: 4.57, Br: 26.11; Found C: 54.63, H: 3.75, N: 4.44, Br: 25.88; IR (KBr, ν, cm^-1^): 3460-3310 (broad band OH), 3056 (CH_arom._), 2942 (CH_aliphatic_), 1664 (C=O); ^1^H-NMR (400 MHz, δ, CDCl_3_): δ 1.25-1.35 (m, 4H, 2CH_2_), 1.77-1.82 (t, *J* = 8.0 Hz, 2H, CH_2_), 1.87-1.92 (t, *J* = 8.0 Hz, 2H, CH_2_), 7.35-7.48 (m, 3H, CH_arom_), 11.66 (br, 1H, COOH); ^13^C NMR (100 MHz, CDCl_3_): δ 22.90, 23.20, 29.10, 33.55, 125.50, 126.67, 127.32, 128.45, 130.95, 132.34, 135.11, 146.67, 150.35, 168.65; MS (EI, m/z): 308 M^+2^, 306 M^+^. |
| --- | --- |

***7-Chloro-1,2,3,4-tetrahydroacridine-9-carboxylic acid 2c:***

|  | Pale yellow crystals, mp: 240-42 °C, (0.25 g, 82%); (C_14_H_12_ClNO_2_, MWt=261.06); Calcd C:64.25, H: 4.62, N: 5.35, Cl: 13.55; Found C: 64.11, H: 4.40, N: 5.08, Cl: 13.07; IR (KBr, ν, cm^-1^): 3460-3310 (broad band OH), 3056 (CH_arom._), 2942 (CH_aliphatic_), 1664 (C=O); ^1^H-NMR (400 MHz, δ, CDCl_3_): δ 1.25-1.35 (m, 4H, 2CH_2_), 1.77-1.82 (t, *J* = 8.0 Hz, 2H, CH_2_), 1.87-1.92 (t, *J* = 8.0 Hz, 2H, CH_2_), 7.35-7.48 (m, 3H, CH_arom_), 12.36 (br, 1H, COOH); ^13^C NMR (100 MHz, CDCl_3_): δ 22.90, 23.20, 29.10, 33.55, 125.50, 126.67, 127.32, 128.45, 130.95, 132.34, 135.11, 146.67, 150.35, 168.65; MS (EI, m/z): 261 M^+2^, 259 M^+^. |
| --- | --- |

***7-Methyl-1,2,3,4-tetrahydroacridine-9-carboxylic acid 2d:***

|  | Yellow crystals, mp: 228-30 °C, (0.25 g, 80%); (C_15_H_15_NO_2_, MWt=241.11); Calcd C: 74.67, H: 6.27, N: 5.81; Found C: 74.11, H: 5.42, N: 5.25; IR (KBr, ν, cm^-1^): 3450-3300 (broad band OH), 3043 (CH_arom._), 2942 (CH_aliphatic_), 1664 (C=O); ^1^H-NMR (400 MHz, δ, CDCl_3_): δ 1.22-1.30 (m, 4H, 2CH_2_), 1.74-1.82 (t, *J* = 8.0 Hz, 2H, CH_2_), 1.85-1.92 (t, *J* = 8.0 Hz, 2H, CH_2_), 2.11 (s, 3H, CH_3_), 7.30-7.45 (m, 3H, CH_arom_), 12.55 (br, 1H, COOH); ^13^C NMR (100 MHz, CDCl_3_): δ 22.90, 23.20, 29.10, 33.55, 42.15, 125.50, 126.67, 127.32, 128.45, 130.95, 132.34, 135.11, 146.67, 150.35, 168.65; MS (EI, m/z):, 241 M^+^. |
| --- | --- |

***Synthesis of 1,2,3,4-tetrahydroacridine-9-carbonyl chloride derivatives 3a-d:***

The reaction flask is charged with 17.8 g (10.9 mL, 0.150 mol) freshly distilled thionyl chloride. Whilst stirring, (0.10 mol) tetrahydroacridine-9-carboxylic acid derivative **2a-d** is added in several portions. The last added portions are firstly insoluble; accordingly stirring with the magnetic stirrer is temporarily not possible. The reaction mixture is initially slowly heated whilst stirring (as far as possible) in an oil bath up to 50 °C bath temperature, afterwards stirring is continued for 2 additional hours at 80 °C bath temperature. After the cooling down of the reaction mixture, the reflux condenser is replaced by a distillation bridge and the excess of thionyl chloride is removed by distillation under reduced pressure (about 20 hPa). A yellowish solid remains as residue.

***1,2,3,4-Tetrahydroacridine-9-carbonyl chloride 3a:***

|  | Yellow crystals, mp: 182-185 °C, (C_14_H_12_ClNO, MWt=245.7), Calcd C: 68.44, H: 4.92, N: 5.70, Cl: 14.43; Found: C: 67.88, H: 4.50, N: 5.45, Cl: 14.02; IR (KBr, ν, cm^-1^): 3044 (CH_arom._), 2936 (CH_aliphatic_), 1748 (C=O), 668 (C-Cl); ^1^H-NMR (400 MHz, δ, CDCl_3_): δ 1.24-1.30 (m, 4H, 2CH_2_), 1.78-1.83 (t, *J* = 8.0 Hz, 2H, CH_2_), 1.87-1.92 (t, *J* = 8.0 Hz, 2H, CH_2_), 7.38-7.55 (m, 4H, CH_arom_); ^13^C NMR (100 MHz, CDCl_3_): δ 22.66, 23.20, 28.12, 32.15, 125.45, 126.40, 127.44, 128.32, 131.25, 132.16, 135.22, 147.55, 151.45, 183.89; MS (EI, m/z): 247 M^+2^. |
| --- | --- |

***7-Bromo-1,2,3,4-tetrahydroacridine-9-carbonyl chloride 3b:***

|  | Brownish crystals, mp: 144-46 °C, (C_14_H_11_BrClNO, MWt=322.97), Calcd C: 51.80, H: 3.42, N: 4.32, Br: 24.62, Cl: 10.92; Found: C: 51.44, H: 3.02, N: 3.87, Br: 24.12, Cl: 10.56; IR (KBr, ν, cm^-1^): 3046 (CH_arom._), 2956 (CH_aliphatic_), 1755 (C=O), 687 (C-Cl); ^1^H-NMR (400 MHz, δ, CDCl_3_): δ 1.24-1.28 (m, 4H, 2CH_2_), 1.77-1.82 (t, *J* = 8.0 Hz, 2H, CH_2_), 1.88-1.92 (t, *J* = 8.0 Hz, 2H, CH_2_), 7.35-7.53 (m, 3H, CH_arom_); ^13^C NMR (100 MHz, CDCl_3_): δ 22.23, 23.18, 28.17, 32.65, 123.43, 126.44, 127.67, 128.65, 131.28, 132.20, 135.28, 147.30, 151.41, 188.60; MS (EI, m/z): 326 M^+2^. |
| --- | --- |

***7-Chloro-1,2,3,4-tetrahydroacridine-9-carbonyl chloride 3c:***

|  | Brownish crystals, mp: 144-46 °C, (C_14_H_11_Cl_2_NO, MWt=297.02), Calcd C: 60.02, H: 3.96, N: 5.00, Cl: 23.31; Found: C: 59.49, H: 3.62, N: 4.66, Cl: 22.87; IR (KBr, ν, cm^-1^): 3046 (CH_arom._), 2956 (CH_aliphatic_), 1755 (C=O), 687 (C-Cl); ^1^H-NMR (400 MHz, δ, CDCl_3_): δ 1.24-1.28 (m, 4H, 2CH_2_), 1.77-1.82 (t, *J* = 8.0 Hz, 2H, CH_2_), 1.88-1.92 (t, *J* = 8.0 Hz, 2H, CH_2_), 7.35-7.53 (m, 3H, CH_arom_); ^13^C NMR (100 MHz, CDCl_3_): δ 22.23, 23.18, 28.17, 32.65, 123.43, 126.44, 127.67, 128.65, 131.28, 132.20, 135.28, 147.30, 151.41, 188.60; MS (EI, m/z): 282 M^+2^. |
| --- | --- |

***7-Methyl-1,2,3,4-tetrahydroacridine-9-carbonyl chloride 3d:***

|  | Brownish crystals, mp: 134-36 °C, (C_15_H_14_ClNO, MWt=259.08.02), Calcd C: 69.36, H: 5.43, N: 5.39, Cl: 13.65; Found: C: 59.49, H: 3.62, N: 4.66, Cl: 22.87; IR (KBr, ν, cm^-1^): 3041 (CH_arom._), 2950 (CH_aliphatic_), 1757 (C=O), 685 (C-Cl); ^1^H-NMR (400 MHz, δ, CDCl_3_): δ 1.22-1.28 (m, 4H, 2CH_2_), 1.76-1.82 (t, *J* = 8.0 Hz, 2H, CH_2_), 1.86-1.92 (t, *J* = 8.0 Hz, 2H, CH_2_), 2.12 (s, 3H, CH_3_), 7.35-7.53 (m, 3H, CH_arom_); ^13^C NMR (100 MHz, CDCl_3_): δ 22.23, 23.18, 28.17, 32.65, 42.26, 123.43, 126.44, 127.67, 128.65, 131.28, 132.20, 135.28, 147.30, 151.41, 188.60; MS (EI, m/z): 259 M^+2^. |
| --- | --- |

***Synthesis of N,N'-(ethane-1,2-diyl)bis(1,2,3,4-tetrahydroacridine-9-carboxamide) derivatives 4a-d:***

Method A: In a round bottomed flask, compound **3a-d** (0.01 mol) was dissolved in absolute ethanol (25 mL) and then was treated with ethylenediamine (different molar ratios, 0.01, 0.02 or 0.03 mol). The reaction mixture was heated under reflux for 3 hrs and after the reaction completion (as monitored by TLC), solvent was evaporated under reduced pressure and the residual mass was then triturated by light petroleum (40-60). The obtained solid was collected and recrystallized from acetonitrile into compounds **4a-d**.

***N,N'-(Ethane-1,2-diyl)bis(1,2,3,4-tetrahydroacridine-9-carboxamide) 4a:***

|  | Pale yellow crystals, mp: 230-233 °C, yield 70%, (C_30_H_30_N_4_O_2_, MWt=478.58), Calcd C: 75.29, H: 6.32, N: 11.71; Found: C: 74.88, H: 3.18, N: 11.56; IR (KBr, ν, cm^-1^): 3275 (NH), 3051 (CH_arom._), 2948 (CH_aliphatic_), 1668 (C=O); ^1^H-NMR (400 MHz, δ, CDCl_3_): δ 1.25-1.30 (m, 8H, 2CH_2_), 1.78-1.82 (t, *J* = 8.0 Hz, 4H, CH_2_), 1.88-1.92 (t, *J* = 8.0 Hz, 4H, CH_2_), 3.33 (s, 4H, 2CH_2_), 7.32-7.53 (m, 8H, CH_arom_), 11.68 (s, 2H, NH exchangeable with D_2_O); ^13^C NMR (100 MHz, CDCl_3_): δ 22.23, 23.18, 28.17, 32.65, 37.2, 123.43, 126.44, 127.67, 128.65, 131.28, 132.20, 135.28, 147.30, 151.41, 188.60; MS (EI, m/z): 478 M^+^. |
| --- | --- |

***N,N'-(Ethane-1,2-diyl)bis(7-bromo-1,2,3,4-tetrahydroacridine-9-carboxamide) 4b:***

|  | Orange needles, mp: 196-198 °C, yield 78%, (C_30_H_28_Br_2_N_4_O_2_, MWt = 634.06), Calcd C: 56.62, H: 4.43, N: 8.80, Br: 25.11; Found: C: 56.22, H: 4.15, N: 8.55, Br: 24.89; IR (KBr, ν, cm^-1^): 3272 (NH), 3055 (CH_arom._), 2936 (CH_aliphatic_), 1665 (C=O); ^1^H-NMR (400 MHz, δ, CDCl_3_): δ 1.25-1.31 (m, 8H, 2CH_2_), 1.77-1.82 (t, *J* = 8.2 Hz, 4H, CH_2_), 1.88-1.92 (t, *J* = 8.2 Hz, 4H, CH_2_), 3.34 (s, 4H, 2CH_2_), 7.35-7.50 (m, 6H, CH_arom_), 11.45 (s, 2H, NH exchangeable with D_2_O); ^13^C NMR (100 MHz, CDCl_3_): δ 22.20, 23.12, 28.15, 32.61, 37.0, 123.41, 126.40, 127.61, 128.60, 131.25, 132.25, 135.25, 147.33, 151.44, 186.68; MS (EI, m/z): 638 M^+2^. |
| --- | --- |

***N,N'-(Ethane-1,2-diyl)bis(7-chloro-1,2,3,4-tetrahydroacridine-9-carboxamide) 4c:***

|  | Brown powder, mp: 208-210 °C, yield 74%, (C_30_H_28_Cl_2_N_4_O_2_, MWt = 546.16), Calcd C: 65.82, H: 5.15, N: 10.23, Cl: 12.95; Found: C: 65.67, H: 4.90, N: 10.01, Cl: 12.64; IR (KBr, ν, cm^-1^): 3187 (2NH), 3064 (CH_arom._), 2956 (CH_aliphatic_), 1677 (C=O; ^1^H-NMR (400 MHz, δ, CDCl_3_): δ 1.25-1.33 (m, 8H, 2CH_2_), 1.78-1.82 (t, *J* = 8.2 Hz, 4H, CH_2_), 1.88-1.93 (t, *J* = 8.2 Hz, 4H, CH_2_), 7.38-7.55 (m, 6H, CH_arom_), 12.77 (s, 2H, NH exchangeable with D_2_O); ^13^C NMR (100 MHz, CDCl_3_): δ 22.15, 23.18, 28.25, 32.72, 37.3, 42.02, 123.55, 126.48, 127.78, 128.67, 131.33, 132.41, 135.32, 147.45, 151.53, 169.66, 188.22; MS (EI, m/z): 546 M^+^. |
| --- | --- |

***N,N'-(Ethane-1,2-diyl)bis(7-methyl-1,2,3,4-tetrahydroacridine-9-carboxamide) 4d:***

|  | Brown powder, mp: 203-205 °C, yield 70%, (C_32_H_34_N_4_O_2_, MWt = 506.27), Calcd C: 75.86, H: 6.76, N: 11.06; Found: C: 75.67, H: 6.50, N: 10.88; IR (KBr, ν, cm^-1^): 3187 (2NH), 3064 (CH_arom._), 2956 (CH_aliphatic_), 1677 (C=O; ^1^H-NMR (400 MHz, δ, CDCl_3_): δ 1.25-1.33 (m, 8H, 2CH_2_), 1.78-1.82 (t, *J* = 8.2 Hz, 4H, CH_2_), 1.88-1.93 (t, *J* = 8.2 Hz, 4H, CH_2_), 2.12 (s, 6H, 2CH_3_), 7.38-7.55 (m, 6H, CH_arom_), 12.77 (s, 2H, NH exchangeable with D_2_O); ^13^C NMR (100 MHz, CDCl_3_): δ 22.15, 23.18, 28.25, 32.72, 42.02, 123.55, 126.48, 127.78, 128.67, 131.33, 132.41, 135.32, 147.45, 151.53, 169.66, 188.22; MS (EI, m/z): 546 M^+^. |
| --- | --- |

***Synthesis of piperazine-1,4-diylbis((1,2,3,4-tetrahydroacridin-9-yl)methanone) derivatives 5a-d:***

A solution of piperazine (1 g, 0.125 mol) in absolute ethanol 50 mL was treated with compound **3a-d** (0.1 mol). The reaction mixture was heated under reflux for 1 h then left to cool. The white needles of compound **5a-d** was collected by filtration, washed with cold ethanol and dried.

***Piperazine-1,4-diylbis((1,2,3,4-tetrahydroacridin-9-yl)methanone) 5a:***

|  | White needles, mp: 282-284 °C, yield 74%, (C_32_H_32_N_4_O_2_, MWt=504.62), Calcd C: 76.16, H: 6.39, N: 11.10; Found: C: 75.65, H: 6.11, N: 10.88; IR (KBr, ν, cm^-1^): 3044 (CH_arom._), 2962 (CH_aliphatic_), 1678 (C=O); ^1^H-NMR (400 MHz, δ, CDCl_3_): δ 1.26-1.32 (m, 8H, 2CH_2_), 1.78-1.82 (t, *J* = 8.0 Hz, 8H, CH_2_), 3.34 (s, 8H, 4CH_2piprazine_), 7.35-7.50 (m, 8H, CH_arom_); ^13^C NMR (100 MHz, CDCl_3_): δ 23.80, 27.12, 31.15, 32.61, 37.00, 39.11, 122.60, 126.40, 127.61, 128.60, 131.05, 132.11, 135.13, 147.45, 151.44, 186.68; MS (EI, m/z): 504 M^+^. |
| --- | --- |

***Piperazine-1,4-diylbis((7-bromo-1,2,3,4-tetrahydroacridin-9-yl)methanone) 5b:***

|  | Light brown sheets, mp: 260-262 °C, yield 76%, (C_32_H_30_Br_2_N_4_O_2_, MWt=660.07), Calcd C: 58.02, H: 4.56, N: 8.46, Br: 24.13; Found: C: 57.88, H: 4.22, N: 8.11, Br: 23.88; IR (KBr, ν, cm^-1^): 3056 (CH_arom._), 2960 (CH_aliphatic_), 1676 (C=O); ^1^H-NMR (400 MHz, δ, CDCl_3_): δ 1.27-1.32 (m, 8H, 2CH_2_), 1.78-1.82 (t, *J* = 8.0 Hz, 4H, CH_2_), 1.88-1.92 (t, *J* = 8.0 Hz, 4H, CH_2_), 3.35 (s, 8H, 4CH_2piprazine_), 7.38-7.55 (m, 6H, CH_arom_); ^13^C NMR (100 MHz, CDCl_3_): δ 23.84, 27.10, 38.19, 32.66, 37.09, 39.45, 122.62, 126.54, 127.44, 128.55, 131.12, 132.32, 135.11, 147.43, 158.23, 188.22; MS (EI, m/z): 660 M^+2^. |
| --- | --- |

***Piperazine-1,4-diylbis((7-chloroo-1,2,3,4-tetrahydroacridin-9-yl)methanone) 5c:***

|  | Greenish crystals, mp: 290-292 °C, yield 70%, (C_32_H_30_Cl_2_N_4_O_2_, MWt=572.17), Calcd C: 67.02, H: 5.27, N: 9.77, Cl: 12.36; Found: C: 66.84, H: 5.03, N: 9.56, Cl: 12.04; IR (KBr, ν, cm^-1^): 2948 (CH_aliphatic_), 1696 (C=O), 1672 (C=O); ^1^H-NMR (400 MHz, δ, CDCl_3_): δ 1.28-1.32 (m, 8H, 2CH_2_), 1.77-1.82 (t, *J* = 8.0 Hz, 4H, CH_2_), 1.87-1.92 (t, *J* = 8.0 Hz, 4H, CH_2_), 1.18 (s, 6H, 2CH_3_), 3.34 (s, 8H, 4CH_2piprazine_), 7.35-7.50 (m, 6H, CH_arom_; ^13^C NMR (100 MHz, CDCl_3_): δ 23.50,. 27.10, 38.19, 32.66, 37.09, 39.45, 41.88, 122.75, 126.75, 127.52, 128.72, 131.33, 132.44, 135.25, 147.56, 158.44, 182.78, 188.35; MS (EI, m/z): 572 M^+^. |
| --- | --- |

***Piperazine-1,4-diylbis((7-methyl-1,2,3,4-tetrahydroacridin-9-yl)methanone) 5d:***

|  | Pale yellow crystals, mp: 272-274 °C, yield 70%, (C_34_H_36_N_4_O_2_, MWt=532.28), Calcd C: 76.66, H: 6.81, N: 10.52; Found: C: 76.34, H: 6.53, N: 10.50; IR (KBr, ν, cm^-1^): 2948 (CH_aliphatic_), 1696 (C=O), 1672 (C=O); ^1^H-NMR (400 MHz, δ, CDCl_3_): δ 1.28-1.32 (m, 8H, 2CH_2_), 1.77-1.82 (t, *J* = 8.0 Hz, 4H, CH_2_), 1.87-1.92 (t, *J* = 8.0 Hz, 4H, CH_2_), 1.18 (s, 6H, 2CH_3_), 3.34 (s, 8H, 4CH_2piprazine_), 7.35-7.50 (m, 6H, CH_arom_; ^13^C NMR (100 MHz, CDCl_3_): δ 23.50,. 27.10, 38.19, 32.66, 37.09, 39.45, 41.88, 122.75, 126.75, 127.52, 128.72, 131.33, 132.44, 135.25, 147.56, 158.44, 182.78, 188.35; MS (EI, m/z): 532 M^+^. |
| --- | --- |

***Synthesis of N-(2-hydroxyethyl)-1,2,3,4-tetrahydroacridine-9-carboxamide derivatives 6a-d:***

Compound **3a-d** (0.01 mol) was dissolved in 50 mL absolute ethanol and then was treated with ethanolamine (0.08 mL, 0.0125 mol). The reaction mixture was heated under reflux for 3 hrs, after reaction completion (as monitored by TLC) ethanol was removed under reduced pressure and the residual mass was then triturated with light petroleum (40-60). The formed solid was collected and recrystallized from acetonitrile into **6a-d**.

***N-(2-Hydroxyethyl)-1,2,3,4-tetrahydroacridine-9-carboxamide 6a:***

|  | Pale yellow crystals, mp: 252-255 °C, yield 65%, (C_16_H_18_N_2_O_2_, MWt=270.32), Calcd C: 71.09, H: 6.71, N: 10.36; Found: C: 70.66, H: 6.38, N: 10.08; IR (KBr, ν, cm^-1^): 3411 (OH), 3252 (NH), 3048 (CH_arom._), 2932 (CH_aliphatic_), 1670 (C=O); ^1^H-NMR (400 MHz, δ, CDCl_3_): δ 1.27-1.32 (m, 4H, 2CH_2_), 1.78-1.82 (t, *J* = 8.1 Hz, 2H, CH_2_), 1.88-1.92 (t, *J* = 8.0 Hz, 2H, CH_2_), 3.23-3.36 (m, 4H, CH_2_), 6.11 (s, 1H, OH exchangeable with D_2_O), 7.30-7.44 (m, 4H, CH_arom_), 11.02 (s, 1H, NH exchangeable with D_2_O); ^13^C NMR (100 MHz, CDCl_3_): δ 21.20, 23.25, 28.33, 32.45, 37.55, 39.60 121.22, 123.43, 127.65, 128.60, 131.38, 132.76, 135.55, 147.56, 151.88, 186.65; MS (EI, m/z): 270 M^+^. |
| --- | --- |

***7-Bromo-N-(2-hydroxyethyl)-1,2,3,4-tetrahydroacridine-9-carboxamide 6b:***

|  | Pale yellow crystals, mp: 212-215 °C, yield 65%, (C_16_H_17_BrN_2_O_2_, MWt=348.05), Calcd C: 55.03, H: 4.91, N: 8.01, Br: 22.87; Found: C: 54.66, H: 4.18, N: 7.76, Br: 22.60; IR (KBr, ν, cm^-1^): 3406 (OH), 3245 (NH), 3040 (CH_arom._), 2944 (CH_aliphatic_), 1674 (C=O); ^1^H-NMR (400 MHz, δ, CDCl_3_): δ 1.26-1.30 (m, 4H, 2CH_2_), 1.77-1.82 (t, *J* = 8.1 Hz, 2H, CH_2_), 1.88-1.93 (t, *J* = 8.0 Hz, 2H, CH_2_), 3.25-3.36 (m, 4H, 2CH_2_), 6.10 (s, 1H, OH exchangeable with D_2_O), 7.36-7.48 (m, 3H, CH_arom_), 11.36 (s, 1H, NH exchangeable with D_2_O); ^13^C NMR (100 MHz, CDCl_3_): δ 21.33, 23.65, 28.55, 32.56, 37.63, 39.73 122.23, 123.88, 127.87, 128.67, 131.44, 132.74, 135.43, 147.22, 158.65, 188.32; MS (EI, m/z): 348 M^+^. |
| --- | --- |

***7-Chloro-N-(2-hydroxyethyl)-1,2,3,4-tetrahydroacridine-9-carboxamide 6c:***

|  | Pale yellow crystals, mp: 212-215 °C, yield 65%, (C_16_H_17_ClN_2_O_2_, MWt=304.10), Calcd C: 63.05, H: 5.62, Cl: 11.63, N: 9.19; Found: C: 62.80, H: 5.34, Cl: 11.38, N: 8.78; IR (KBr, ν, cm^-1^): 3412 (OH), 3245 (NH), 3040 (CH_arom._), 2944 (CH_aliphatic_), 1674 (C=O); ^1^H-NMR (400 MHz, δ, CDCl_3_): δ 1.26-1.30 (m, 4H, 2CH_2_), 1.77-1.82 (t, *J* = 8.1 Hz, 2H, CH_2_), 1.88-1.93 (t, *J* = 8.0 Hz, 2H, CH_2_), 3.25-3.36 (m, 4H, 2CH_2_), 6.15 (s, 1H, OH exchangeable with D_2_O), 7.36-7.48 (m, 3H, CH_arom_), 11.36 (s, 1H, NH exchangeable with D_2_O); ^13^C NMR (100 MHz, CDCl_3_): δ 21.33, 23.65, 28.55, 32.56, 37.63, 39.73 122.23, 123.88, 127.87, 128.67, 131.44, 132.74, 135.43, 147.22, 158.65, 188.32; MS (EI, m/z): 304 M^+^. |
| --- | --- |

***N-(2-hydroxyethyl)-7-methyl-1,2,3,4-tetrahydroacridine-9-carboxamide 6d:***

|  | Pale yellow crystals, mp: 212-215 °C, yield 61%, (C_17_H_20_N_2_O_2_, MWt=284.15), Calcd C: 71.81, H: 7.09, N: 9.85; Found: C: 71.55, H: 6.72, N: 9.60; IR (KBr, ν, cm^-1^): 3412 (OH), 3245 (NH), 3040 (CH_arom._), 2944 (CH_aliphatic_), 1674 (C=O); ^1^H-NMR (400 MHz, δ, CDCl_3_): δ 1.26-1.30 (m, 4H, 2CH_2_), 1.77-1.82 (t, *J* = 8.1 Hz, 2H, CH_2_), 1.88-1.93 (t, *J* = 8.0 Hz, 2H, CH_2_), 2.11, (s, 3H, CH_3_), 3.25-3.36 (m, 4H, 2CH_2_), 6.10 (s, 1H, OH exchangeable with D_2_O), 7.36-7.48 (m, 3H, CH_arom_), 11.36 (s, 1H, NH exchangeable with D_2_O); ^13^C NMR (100 MHz, CDCl_3_): δ 21.33, 23.65, 28.55, 32.56, 37.63, 39.73, 42.23, 122.23, 123.88, 127.87, 128.67, 131.44, 132.74, 135.43, 147.22, 158.65, 188.32; MS (EI, m/z): 284 M^+^. |
| --- | --- |

***Synthesis of N-(2-(3-phenylureido[thioueido])ethyl)-1,2,3,4-tetrahydroacridine-9-carboxamide derivatives 7a-f:***

To a solution of compound **3a-d** (0.01 mol) in 50 mL absolute ethanol, 4-phenyl semicarbazide or 4-phenyl thiosemicarbazide (0.012 mol) was added. The reaction mixture was heated under reflux and after reaction completion (2 hrs as monitored by TLC), solvent was removed under reduced pressure and the residual mass was quenched with crushed ice water, left overnight. The formed solid was collected by filtration, washed thoroughly (water) and then recrystallized from proper solvent where compounds 7a-f were obtained.

***N-(2-(3-Phenylureido)ethyl)-1,2,3,4-tetrahydroacridine-9-carboxamide 7a:***

|  | Yellow needles (MeOH), mp: 266-268 °C, yield 80%, (C_23_H_24_N_4_O_2_, MWt=388.46), Calcd C: 71.04, H: 6.17, N: 14.41; Found: C: 69.68, H: 5.90, N: 14.02; IR (KBr, *ν*, cm^-1^): 3255, 3210, 3176 (3NH), 3054 (CH_arom._), 2938 (CH_aliphatic_), 1678 (C=O), 1655 (C=O); ^1^H-NMR (400 MHz, *δ,* CDCl_3_): δ 1.26-1.30 (m, 4H, 2CH_2_), 1.77-1.82 (t, *J* = 8.0 Hz, 2H, CH_2_), 1.87-1.92 (t, *J* = 8.0 Hz, 2H, CH_2_), 3.28-3.36 (m, 4H, 2CH_2_), 7.32-7.44 (m, 4H, CH_arom_), 8.66 (s, 1H, NH exchangeable with D_2_O), 9.26 (s, 1H, NH exchangeable with D_2_O), 11.25 (s, 1H, NH exchangeable with D_2_O); ^13^C NMR (100 MHz, CDCl_3_): δ 21.03, 23.22, 28.34, 32.50, 41.88, 42.14, 121.66, 121.98, 122.25, 123.02, 123.55, 124.51, 127.89, 128.40, 131.51, 132.75, 135.21, 147.30, 158.65, 169.78, 188.65; MS (EI, m/z): 388 M^+^. |
| --- | --- |

***7-Bromo-N-(2-(3-phenylureido)ethyl)-1,2,3,4-tetrahydroacridine-9-carboxamide 7b:***

|  | Brownish crystals (MeCN), mp: 230-23 °C, yield 86%, (C_23_H_23_BrN_4_O_2_, MWt=467.35), Calcd C: 59.05, H: 4.92, N: 11.98, Br: 17.09; Found: C: 58.67, H: 4.56, N: 11.62, Br: 16.76; IR (KBr, ν, cm^-1^): 3278, 3212, 3185 (3NH), 3050 (CH_arom._), 2942 (CH_aliphatic_), 1676 (C=O), 1658 (C=O); ^1^H-NMR (400 MHz, *δ*, CDCl_3_): δ 1.27-1.32 (m, 4H, 2CH_2_), 1.78-1.82 (t, *J* = 8.0 Hz, 2H, CH_2_), 1.88-1.92 (t, *J* = 8.0 Hz, 2H, CH_2_), 3.26-3.36 (m, 4H, 2CH_2_), 7.38-7.45 (m, 3H, CH_arom_), 8.67 (s, 1H, NH exchangeable with D_2_O), 9.22 (s, 1H, NH exchangeable with D_2_O), 11.34 (s, 1H, NH exchangeable with D_2_O); ^13^C NMR (100 MHz, CDCl_3_): δ 21.25, 23.43, 28.56, 32.76, 41.89, 42.23, 121.71, 121.90, 122.33, 123.21, 123.32, 124.42, 127.61, 128.68, 131.31, 132.67, 135.05, 147.62, 158.73, 169.44, 188.63; MS (EI, m/z): 469 M^+2^. |
| --- | --- |

***7-Chloro-N-(2-(3-phenylureido)ethyl)-1,2,3,4-tetrahydroacridine-9-carboxamide 7c:***

|  | Brownish crystals (MeCN), mp: 230-23 °C, yield 86%, (C_23_H_23_ClN_4_O_2_, MWt=422.15), Calcd C: 65.32, H: 5.48, Cl: 8.38, N: 13.25; Found: C: 65.02, H: 5.14, Cl: 8.01, N: 10.87; IR (KBr, ν, cm^-1^): 3278, 3212, 3185 (3NH), 3050 (CH_arom._), 2942 (CH_aliphatic_), 1676 (C=O), 1658 (C=O); ^1^H-NMR (400 MHz, *δ,* CDCl_3_): δ 1.27-1.32 (m, 4H, 2CH_2_), 1.78-1.82 (t, *J* = 8.0 Hz, 2H, CH_2_), 1.88-1.92 (t, *J* = 8.0 Hz, 2H, CH_2_), 3.26-3.36 (m, 4H, 2CH_2_), 7.38-7.45 (m, 3H, CH_arom_), 8.67 (s, 1H, NH exchangeable with D_2_O), 9.22 (s, 1H, NH exchangeable with D_2_O), 11.34 (s, 1H, NH exchangeable with D_2_O); ^13^C NMR (100 MHz, CDCl_3_): δ 21.25, 23.43, 28.56, 32.76, 41.89, 42.23, 121.71, 121.90, 122.33, 123.21, 123.32, 124.42, 127.61, 128.68, 131.31, 132.67, 135.05, 147.62, 158.73, 169.44, 188.63; MS (EI, m/z): 424 M^+2^. |
| --- | --- |

***7-Methyl-N-(2-(3-phenylureido)ethyl)-1,2,3,4-tetrahydroacridine-9-carboxamide 7d:***

|  | Brownish crystals (MeCN), mp: 230-23 °C, yield 86%, (C_24_H_26_N_4_O_2_, MWt=402.21), Calcd C: 71.62, H: 6.51, N: 13.92; Found: C: 71.40, H: 6.23, N: 13.67; IR (KBr, ν, cm^-1^): 3278, 3212, 3185 (3NH), 3050 (CH_arom._), 2942 (CH_aliphatic_), 1676 (C=O), 1658 (C=O); ^1^H-NMR (400 MHz, *δ*, CDCl_3_): δ 1.27-1.32 (m, 4H, 2CH_2_), 1.78-1.82 (t, *J* = 8.0 Hz, 2H, CH_2_), 1.88-1.92 (t, *J* = 8.0 Hz, 2H, CH_2_), 2.12 (s, 3H, CH_3_), 3.26-3.36 (m, 4H, 2CH_2_), 7.38-7.45 (m, 3H, CH_arom_), 8.67 (s, 1H, NH exchangeable with D_2_O), 9.22 (s, 1H, NH exchangeable with D_2_O), 11.34 (s, 1H, NH exchangeable with D_2_O); ^13^C NMR (100 MHz, CDCl_3_): δ 21.25, 23.43, 28.56, 32.76, 41.89, 42.23, 44.68, 121.71, 121.90, 122.33, 123.21, 123.32, 124.42, 127.61, 128.68, 131.31, 132.67, 135.05, 147.62, 158.73, 169.44, 188.63; MS (EI, m/z): 402 M^+^. |
| --- | --- |

***N-(2-(3-Phenylthioureido)ethyl)-1,2,3,4-tetrahydroacridine-9-carboxamide 7e:***

|  | Pale yellow crystals (dioxane), mp: 186-188 °C, yield 72%, (C_23_H_24_N_4_OS, MWt=404.52), Calcd C: 68.22, H: 5.93, N: 13.84, S: 7.91; Found: C: 67.87, H: 5.90, N: 13.56, S: 7.50; IR (KBr, *ν*, cm^-1^): 3260, 3217, 3176 (3NH), 3066 (CH_arom._), 2940 (CH_aliphatic_), 1676 (C=O), 1657 (C=O), 1325 (C=S); ^1^H-NMR (400 MHz, *δ*, CDCl_3_): δ 1.26-1.30 (m, 4H, 2CH_2_), 1.77-1.82 (t, *J* = 8.0 Hz, 2H, CH_2_), 1.87-1.92 (t, *J* = 8.0 Hz, 2H, CH_2_), 3.28-3.36 (m, 4H, 2CH_2_), 7.32-7.44 (m, 4H, CH_arom_), 8.66 (s, 1H, NH exchangeable with D_2_O), 9.26 (s, 1H, NH exchangeable with D_2_O), 11.25 (s, 1H, NH exchangeable with D_2_O); ^13^C NMR (100 MHz, CDCl_3_): δ 21.33, 23.41, 28.42, 32.56, 41.81, 42.22, 121.34, 121.90, 122.21, 123.22, 123.50, 124.67, 127.87, 128.55, 131.67, 132.88, 135.32, 147.42, 158.65, 181.08, 188.45; MS (EI, m/z): 404 M^+^. |
| --- | --- |

***7-Bromo-N-(2-(3-phenylthioureido)ethyl)-1,2,3,4-tetrahydroacridine-9-carboxamide 7f:***

|  | Light brown crystals (CH_3_CN), mp: 166-168 °C, yield 76%, (C_23_H_23_BrN_4_OS, MWt=483.42), Calcd C: 57.09, H: 4.75, N: 11.58, S: 6.61, Br: 16.52; Found: C: 56.76, H: 4.43, N: 11.42, S: 6.35, Br: 16.08; IR (KBr, ν, cm^-1^): 3266, 3216, 3170 (3NH), 3065 (CH_arom._), 2944 (CH_aliphatic_), 1677 (C=O), 1658 (C=O), 1320 (C=S); ^1^H-NMR (400 MHz, *δ*, CDCl_3_): δ 1.27-1.31 (m, 4H, 2CH_2_), 1.78-1.82 (t, *J* = 8.0 Hz, 2H, CH_2_), 1.88-1.92 (t, *J* = 8.0 Hz, 2H, CH_2_), 3.28-3.35 (m, 4H, 2CH_2_), 7.32-7.44 (m, 3H, CH_arom_), 8.63 (s, 1H, NH exchangeable with D_2_O), 9.25 (s, 1H, NH exchangeable with D_2_O), 11.27 (s, 1H, NH exchangeable with D_2_O); ^13^C NMR (100 MHz, CDCl_3_): δ 21.30, 23.43, 28.40, 32.56, 41.87, 42.28, 121.37, 121.93, 122.24, 123.23, 123.54, 124.66, 127.84, 128.50, 131.63, 132.80, 135.35, 147.47, 158.60, 181.23, 188.87; MS (EI, m/z): 485 M^+2^. |
| --- | --- |

***7-Chloro-N-(2-(3-phenylthioureido)ethyl)-1,2,3,4-tetrahydroacridine-9-carboxamide 7g:***

|  | Light brown crystals (CH_3_CN), mp: 181-183 °C, yield 75%, (C_23_H_23_ClN_4_OS, MWt=438.97), Calcd C: 62.93, H: 5.28, Cl: 8.08, N: 12.76, S: 7.30; Found: C: 62.55, H: 5.01, N: 12.66, S: 7.09; IR (KBr, *ν,* cm^-1^): 3266, 3216, 3170 (3NH), 3065 (CH_arom._), 2944 (CH_aliphatic_), 1677 (C=O), 1658 (C=O), 1320 (C=S); ^1^H-NMR (400 MHz, *δ,* CDCl_3_): δ 1.27-1.31 (m, 4H, 2CH_2_), 1.78-1.82 (t, *J* = 8.0 Hz, 2H, CH_2_), 1.88-1.92 (t, *J* = 8.0 Hz, 2H, CH_2_), 3.28-3.35 (m, 4H, 2CH_2_), 7.32-7.44 (m, 3H, CH_arom_), 8.63 (s, 1H, NH exchangeable with D_2_O), 9.25 (s, 1H, NH exchangeable with D_2_O), 11.27 (s, 1H, NH exchangeable with D_2_O); ^13^C NMR (100 MHz, CDCl_3_): δ 21.30, 23.43, 28.40, 32.56, 41.87, 42.28, 121.37, 121.93, 122.24, 123.23, 123.54, 124.66, 127.84, 128.50, 131.63, 132.80, 135.35, 147.47, 158.60, 181.23, 188.87; MS (EI, m/z): 440 M^+2^. |
| --- | --- |

***7-Methyl-N-(2-(3-phenylthioureido)ethyl)-1,2,3,4-tetrahydroacridine-9-carboxamide 7h:***

|  | Light brown crystals (CH_3_CN), mp: 202-205 °C, yield 75%, (C_24_H_26_N_4_OS, MWt=418.18), Calcd C: 68.87, H: 6.26, N: 13.39, S: 7.66; Found: C: 68.54, H: 6.01, N: 13.12, S: 7.39; IR (KBr, *ν,* cm^-1^): 3266, 3216, 3170 (3NH), 3065 (CH_arom._), 2944 (CH_aliphatic_), 1677 (C=O), 1658 (C=O), 1320 (C=S); ^1^H-NMR (400 MHz, *δ*, CDCl_3_): δ 1.27-1.31 (m, 4H, 2CH_2_), 1.78-1.82 (t, *J* = 8.0 Hz, 2H, CH_2_), 1.88-1.92 (t, *J* = 8.0 Hz, 2H, CH_2_), 2.11 (s, 3H, CH_3_), 3.28-3.35 (m, 4H, 2CH_2_), 7.32-7.44 (m, 3H, CH_arom_), 8.63 (s, 1H, NH exchangeable with D_2_O), 9.25 (s, 1H, NH exchangeable with D_2_O), 11.27 (s, 1H, NH exchangeable with D_2_O); ^13^C NMR (100 MHz, CDCl_3_): δ 21.30, 23.43, 28.40, 32.56, 41.87, 42.28, 121.37, 121.93, 122.24, 123.23, 123.54, 124.66, 127.84, 128.50, 131.63, 132.80, 135.35, 147.47, 158.60, 181.23, 188.87; MS (EI, m/z): 418 M^+^. |
| --- | --- |

***Synthesis of 2-(1,2,3,4-tetrahydroacridine-9-carbonyl)hydrazinecarbothioamide derivatives 8a-d:***

A mixture of compound **3a-d** (0.01 mol) in 50 mL absolute ethanol was treated with thiosemicarbazide (1.1g, 0.012 mol). The reaction mixture was heated under reflux for 3 hrs, after reaction completion (monitored by TLC), solvent was removed under reduced pressure and the crushed-ice water was added. The formed solid was collected by filtration, washed (water) and recrystallized from acetonitrile into compounds **8a-d**.

***2-(1,2,3,4-Tetrahydroacridine-9-carbonyl)hydrazinecarbothioamide 8a:***

|  | Pale yellow needles mp: 174-176 °C, yield 82%, (C_15_H_16_N_4_OS, MWt=300.38), Calcd C: 59.92, H: 5.32, N: 18.64, S: 10.65; Found: C: 59.58, H: 5.01, N: 18.34, S: 10.33; IR (KBr, *ν,* cm^-1^): 3318, 3232, 3170 (NH_2_, 2NH), 3058 (CH_arom._), 2943 (CH_aliphatic_), 1672 (C=O), 1452 (C=S); ^1^H-NMR (400 MHz, *δ,* CDCl_3_): δ 1.27-1.31 (m, 4H, 2CH_2_), 1.78-1.82 (t, *J* = 8.0 Hz, 2H, CH_2_), 1.88-1.92 (t, *J* = 8.0 Hz, 2H, CH_2_), 7.32-7.44 (m, 4H, CH_arom_), 8.12 (s, 2H, NH_2_ exchangeable with D_2_O), 9.23 (s, 1H, NH exchangeable with D_2_O), 11.20 (s, 1H, NH exchangeable with D_2_O); ^13^C NMR (100 MHz, CDCl_3_): δ 21.19, 23.28, 28.74, 32.51, 121.62, 122.28, 123.50, 124.54, 127.81, 128.46, 135.27, 147.33, 158.61, 169.67, 180.60; MS (EI, m/z): 300 M^+^. |
| --- | --- |

***2-(7-Bromo-1,2,3,4-tetrahydroacridine-9-carbonyl)hydrazinecarbothioamide 8b:***

|  | Brownish crystals mp: 158-160 °C, yield 86%, (C_15_H_15_BrN_4_OS, MWt=379.27), Calcd C: 47.45, H: 3.95, N: 14.76, S: 8.43, Br: 21.06; Found: C: 47.07, H: 4.01, N: 14.39, S: 8.20, Br: 20.77; IR (KBr, ν, cm^-1^): 3323, 3230, 3178 (NH_2_, 2NH), 3045 (CH_arom._), 2956 (CH_aliphatic_), 1678 (C=O), 1458 (C=S); ^1^H-NMR (400 MHz, δ, CDCl_3_): δ 1.26-1.31 (m, 4H, 2CH_2_), 1.77-1.82 (t, *J* = 8.0 Hz, 2H, CH_2_), 1.88-1.92 (t, *J* = 8.0 Hz, 2H, CH_2_), 7.32-7.44 (m, 3H, CH_arom_), 8.18 (s, 2H, NH_2_ exchangeable with D_2_O), 9.25 (s, 1H, NH exchangeable with D_2_O), 11.28 (s, 1H, NH exchangeable with D_2_O); ^13^C NMR (100 MHz, CDCl_3_): δ 21.22, 23.23, 28.77, 32.55, 121.66, 122.23, 123.51, 124.57, 127.85, 128.41, 135.20, 147.88, 158.66, 169.87, 180.77; MS (EI, m/z): 381 M^+2^. |
| --- | --- |

***2-(7-Chloro-1,2,3,4-tetrahydroacridine-9-carbonyl)hydrazinecarbothioamide 8c:***

|  | Yellow crystals, mp: 162-164 °C, yield 80%, (C_17_H_19_N_5_O_2_S, MWt=357.43), Calcd C: 57.07, H: 5.13, N: 19.58, S: 8.95; Found: C: 56.76, H: 4.87, N: 19.26, S: 8.65; IR (KBr, *ν,* cm^-1^): 3318, 3268, 3215, 3170 (2NH, NH_2_), 3054 (CH_arom._), 2948 (CH_aliphatic_), 1677 (C=O), 1658 (C=O), 1350 (C=S); ^1^H-NMR (400 MHz, *δ*, CDCl_3_): δ 1.28-1.32 (m, 4H, 2CH_2_), 1.78-1.82 (t, *J* = 8.0 Hz, 2H, CH_2_), 1.88-1.92 (t, *J* = 8.0 Hz, 2H, CH_2_), 2.22 (s, 3H, CH_3_), 6.62 (br, 2H, NH_2_ exchangeable with D_2_O), 7.36-7.45 (m, 3H, CH_arom_), 9.22 (s, 1H, NH exchangeable with D_2_O), 11.23 (s, 1H, NH exchangeable with D_2_O); ^13^C NMR (100 MHz, CDCl_3_): δ 21.30, 23.44, 28.35, 32.53, 42.28, 121.61, 122.26, 123.15, 127.22, 128.47, 131.50, 132.73, 135.24, 147.31, 169.61, 185.12, 188.60; MS (EI, m/z): 359 M^+^. |
| --- | --- |

***2-(7-Methyl-1,2,3,4-tetrahydroacridine-9-carbonyl)hydrazinecarbothioamide 8d:***

|  | Pale yellow crystals mp: 173-175 °C, yield 84%, (C_16_H_18_N_4_OS, MWt=314.12), Calcd C: 61.12, H: 5.77, N: 17.82, S: 10.20; Found: C: 60.89, H: 5.50, N: 17.61, S: 10.02; IR (KBr, *ν,* cm^-1^): 3323, 3230, 3178 (NH_2_, 2NH), 3045 (CH_arom._), 2956 (CH_aliphatic_), 1678 (C=O), 1458 (C=S); ^1^H-NMR (400 MHz, *δ*, CDCl_3_): δ 1.26-1.31 (m, 4H, 2CH_2_), 1.77-1.82 (t, *J* = 8.0 Hz, 2H, CH_2_), 1.88-1.92 (t, *J* = 8.0 Hz, 2H, CH_2_), 2.12 (s, 3H, CH_3_), 7.32-7.44 (m, 3H, CH_arom_), 8.18 (s, 2H, NH_2_ exchangeable with D_2_O), 9.25 (s, 1H, NH exchangeable with D_2_O), 11.28 (s, 1H, NH exchangeable with D_2_O); ^13^C NMR (100 MHz, CDCl_3_): δ 21.22, 23.23, 28.77, 32.55, 121.66, 122.23, 123.51, 124.57, 127.85, 128.41, 135.20, 147.88, 158.66, 169.87, 180.77; MS (EI, m/z): 314 M^+^. |
| --- | --- |


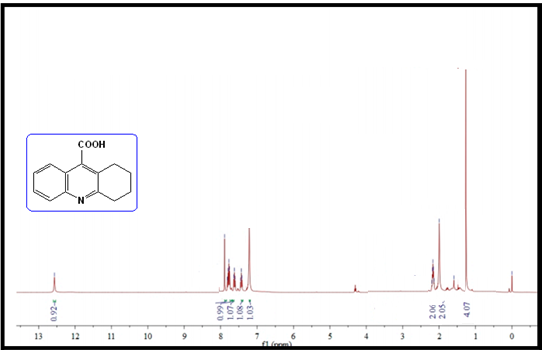


^1^H-NMR spectrum of compound **2a**.


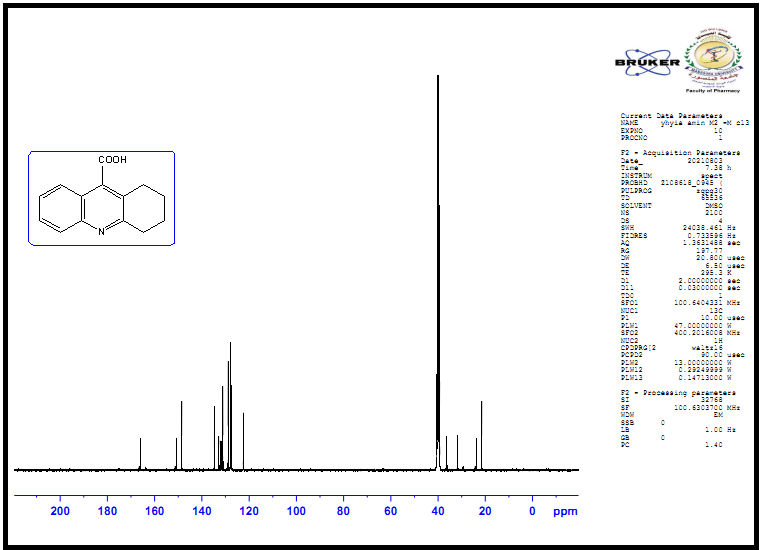


^13^CMR spectrum of compound **2a**.


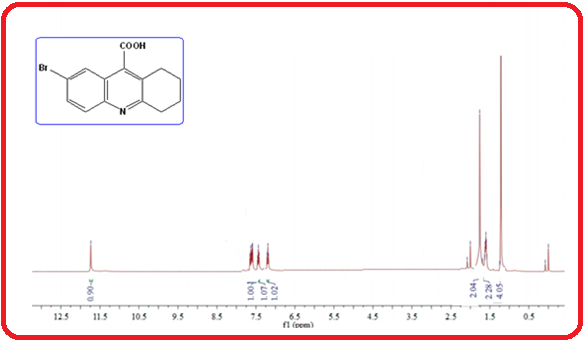


^1^H-NMR spectrum of compound **2b**.

***
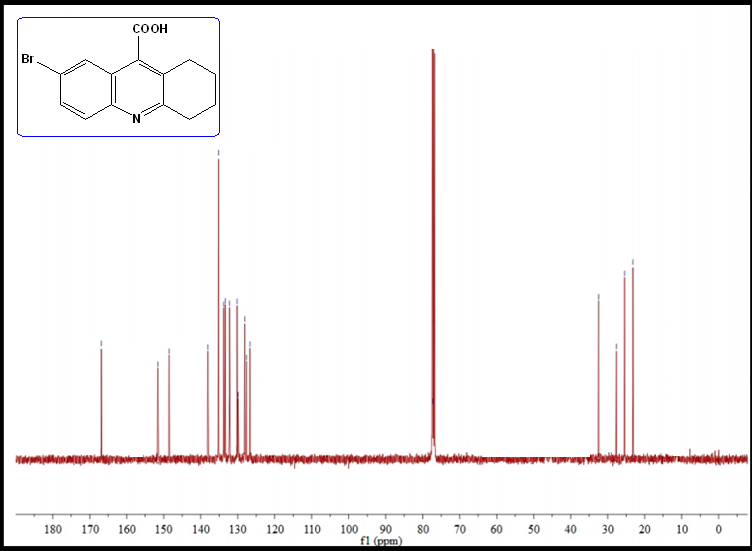
***

^13^CMR spectrum of compound **2b**.


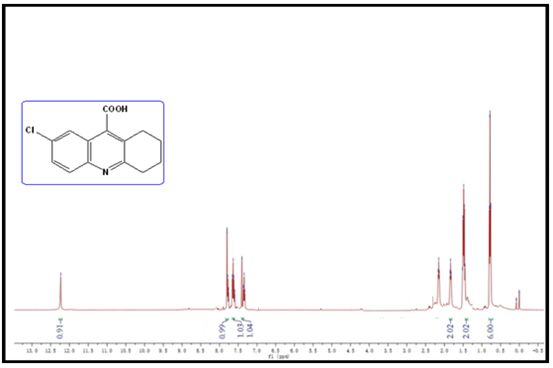


^1^H-NMR spectrum of compound **2c**:


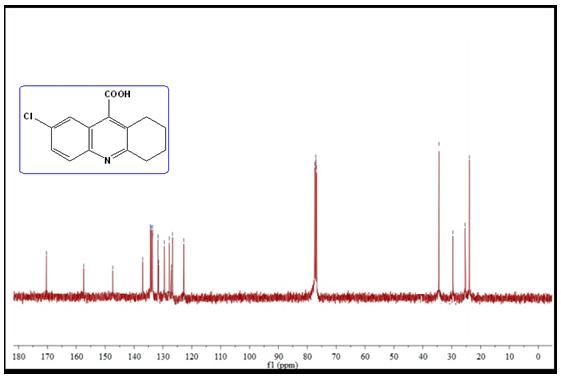


^13^CMR spectrum of compound **2c**:


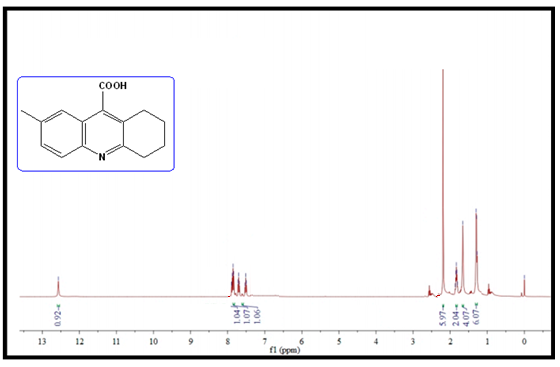


^1^H-NMR spectrum mof compound **2d**.


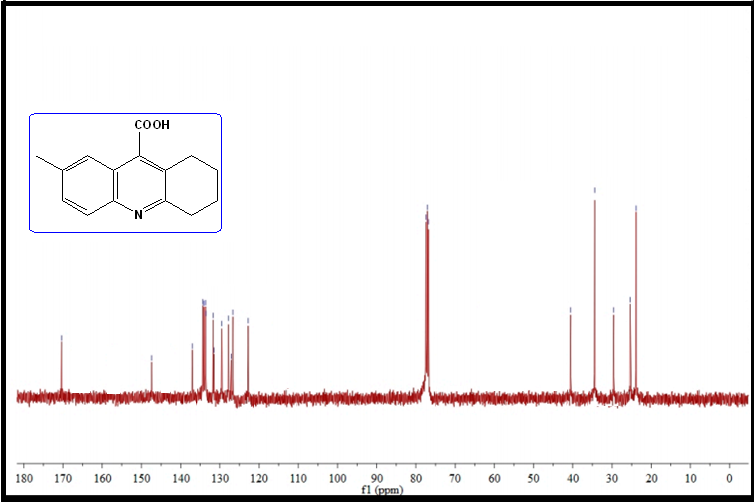


^13^CMR spectrum mof compound **2d**.


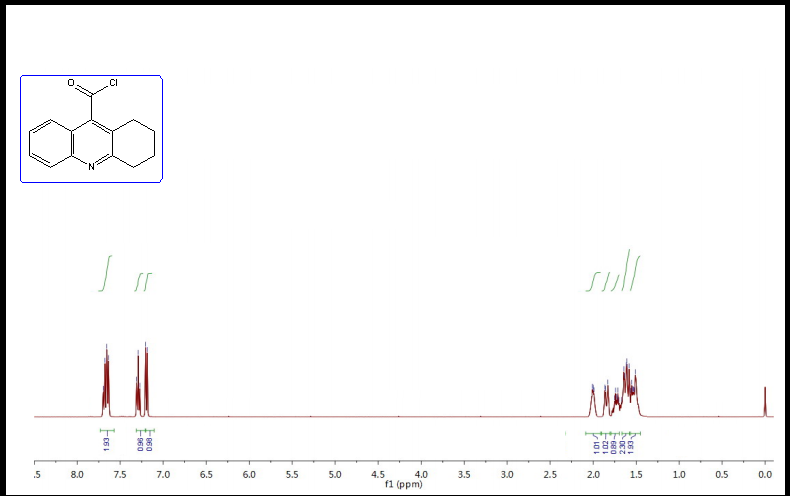


^1^H-NMR spectrum of compound **3a**.


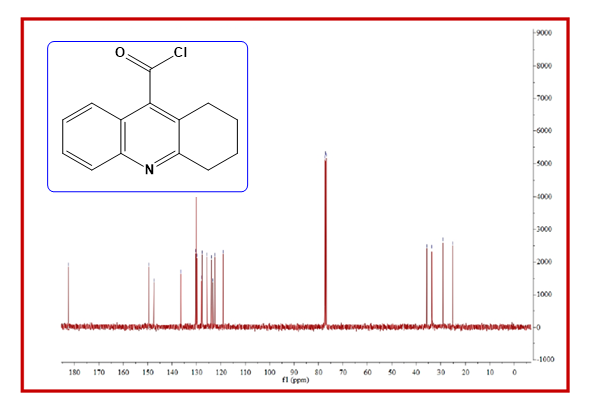


^13^CMR spectrum of compound **3a**.


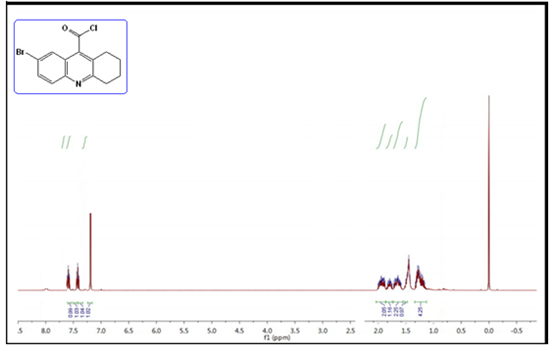


^1^H-NMR spectrum of compound **3b**.


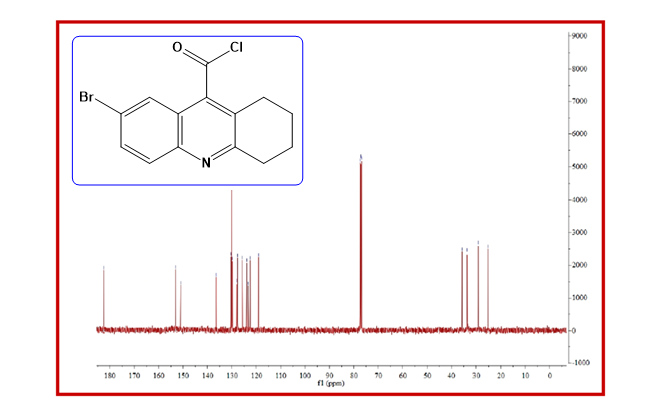


^13^CMR of compound **3b**.

***
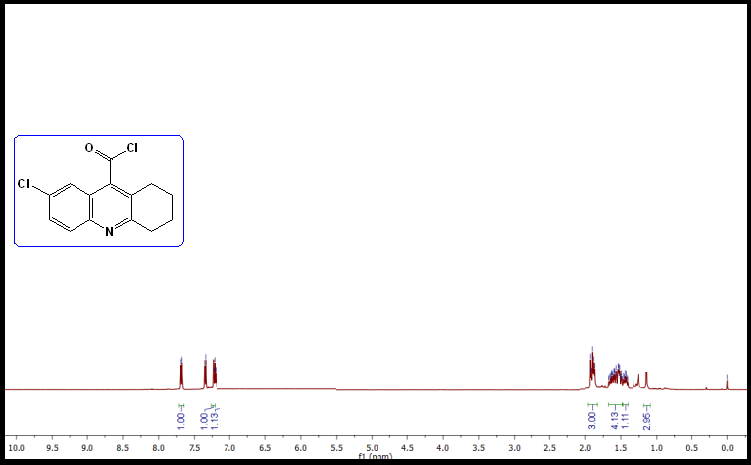
***

^1^H-NMR spectrum of compound **3c**.


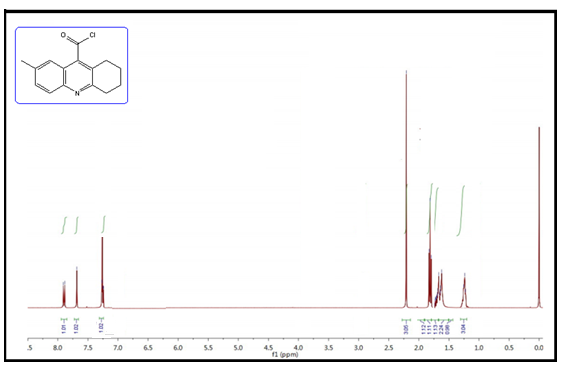


^1^H-NMR spectrum of compound **3d**.


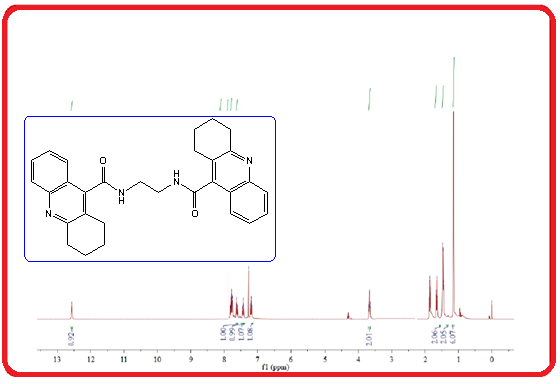


^1^H-NMR spectrum of compound **4a**.


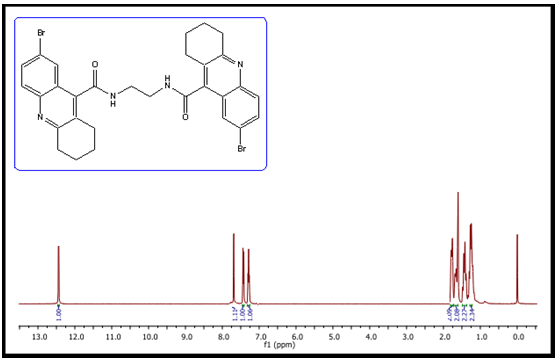


^1^H-NMR spectrum of compound **4b**.


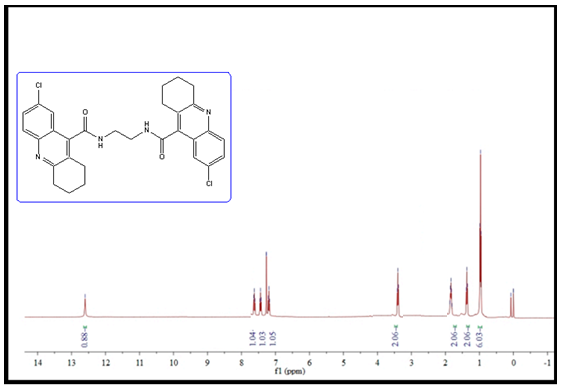


^1^H-NMR spectrum of compound **4c**.


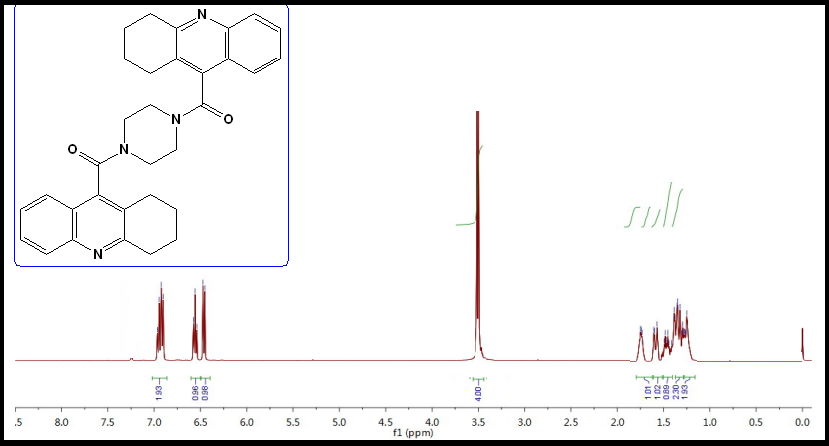


^1^H-NMR spectrum of compound **5a**.


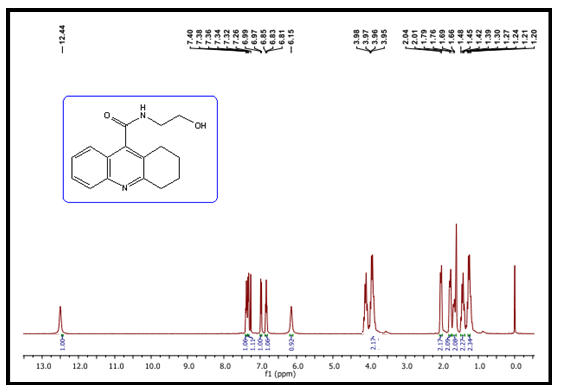


^1^H-NMR spectrum of compound **6a**.


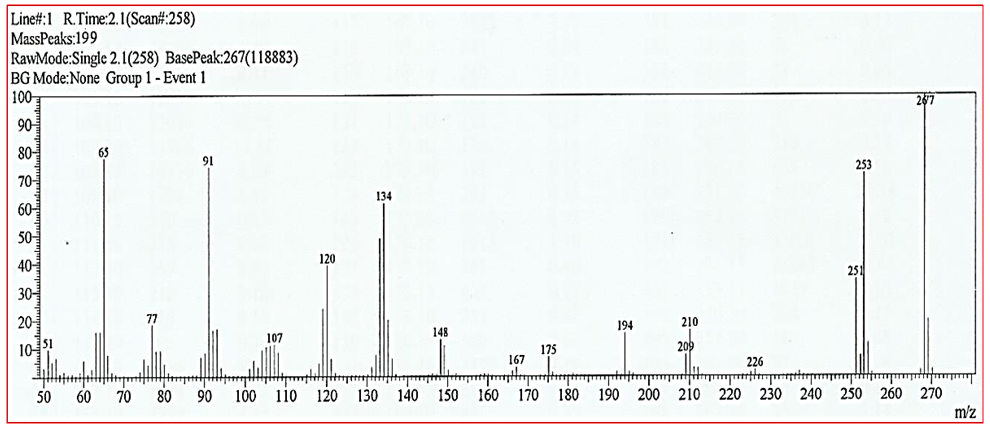


Mass spectrum of compound **6a**.


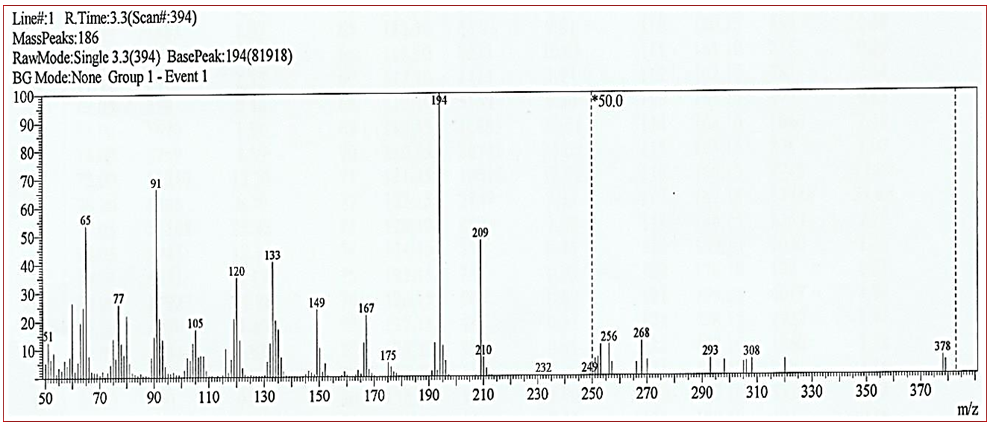


Mass spectrum of compound **8b**.

Supplementary Information of *in-silico* study

Figures (S1-S4) of DPP-IV (PDB ID: 4a5s) for **Comp5a, Comp4d, Comp4c** and **Comp 4b**, respectively.


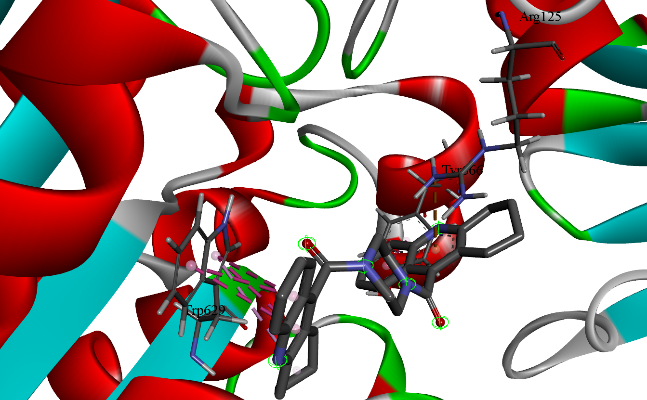

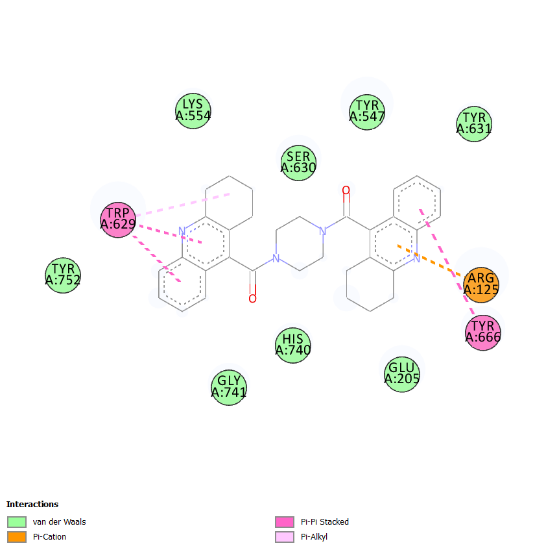


**Figure S1: Comp 5a**


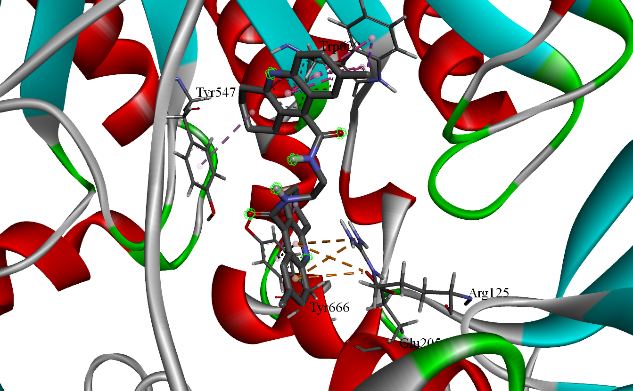

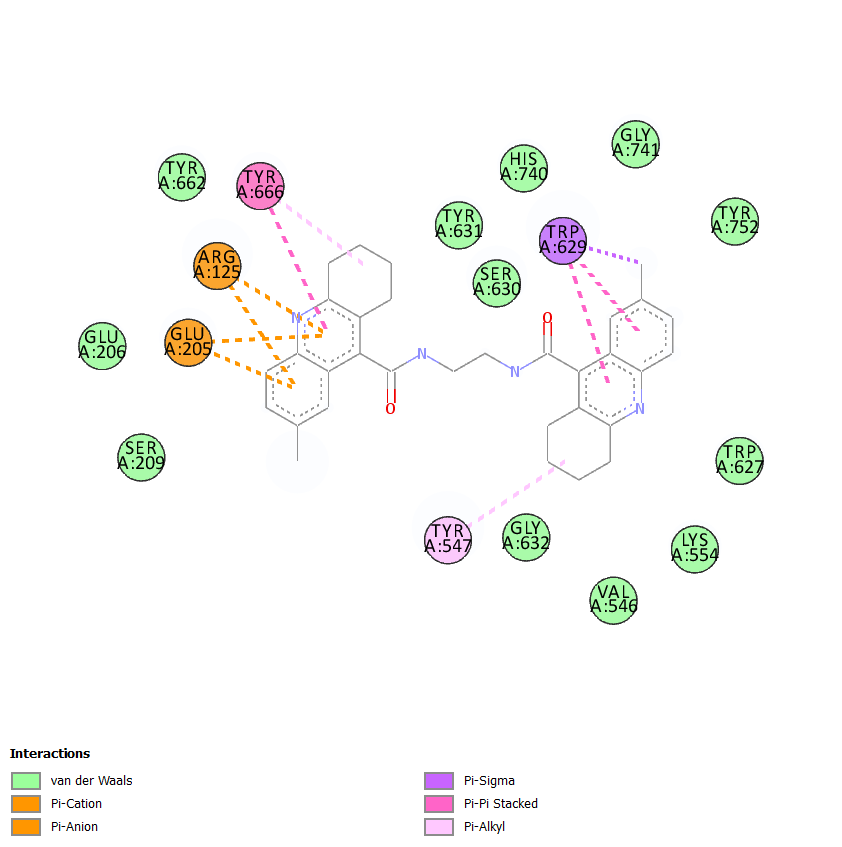


**Figure S2: Comp 4d**


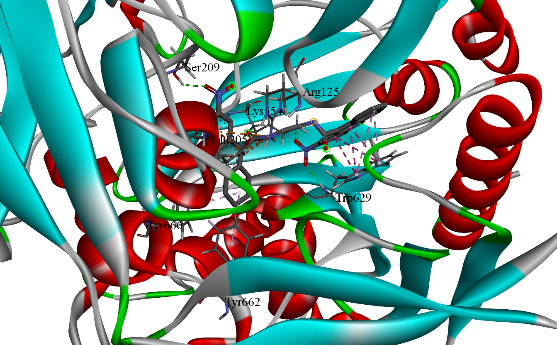

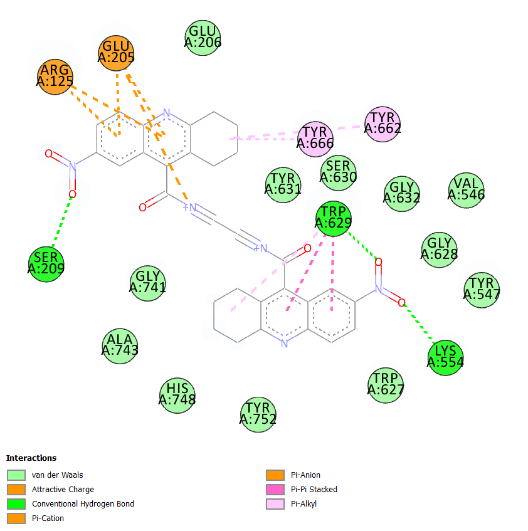


**Figure S3: Comp 4c**


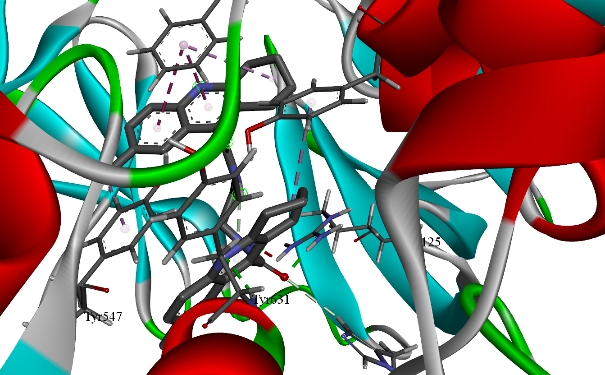

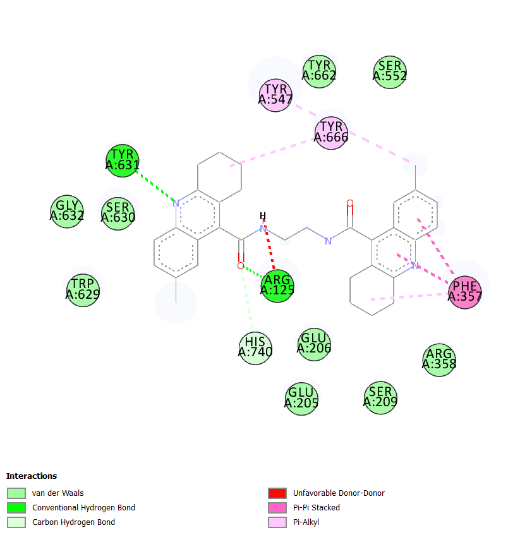


**Figure S4: Comp 4b**

***Figures (S5-S8) of SGLT1 (PDB ID: 3dh4) for Comp4a, Comp4b, Comp5a and Comp7a, respectively.***


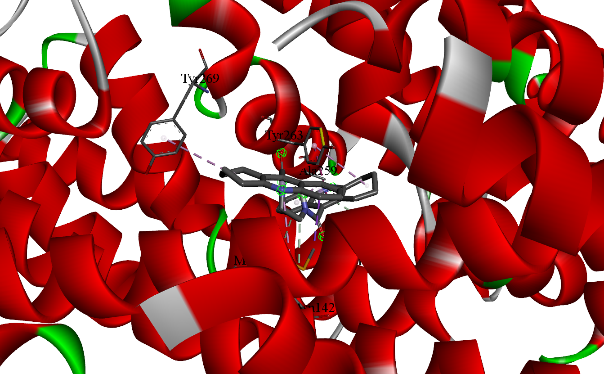

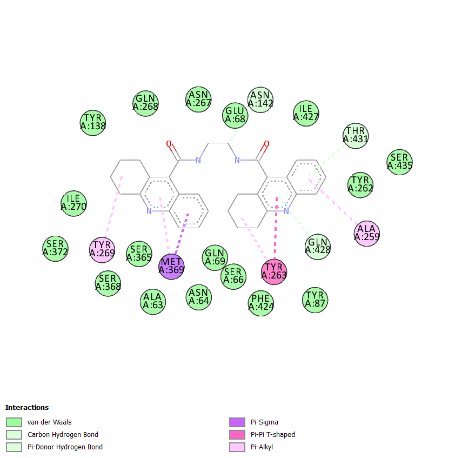


**Figure S5: Comp4a**


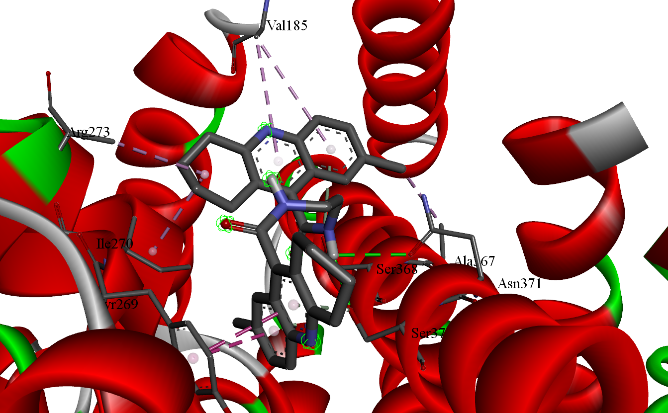

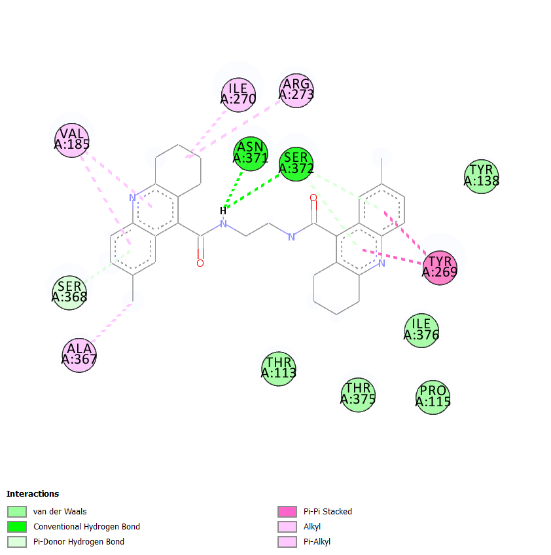


**Figure S6: Comp4b**


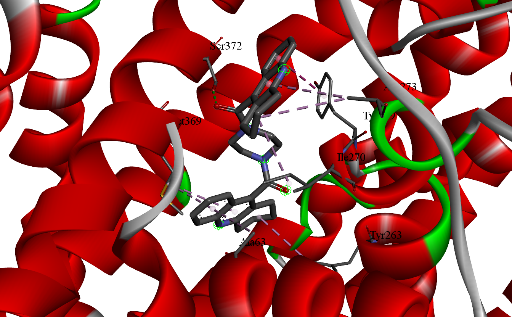

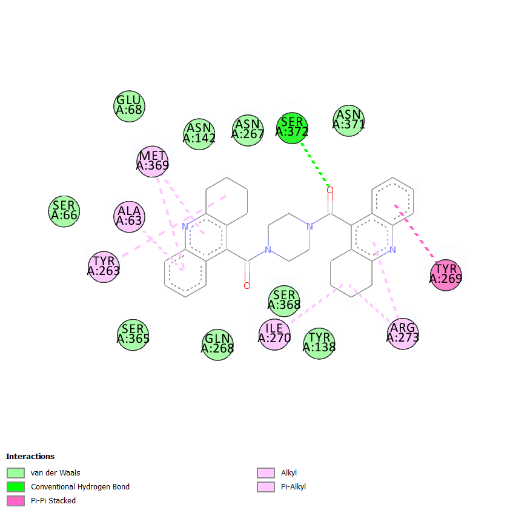


**Figure S7: Comp 5a**


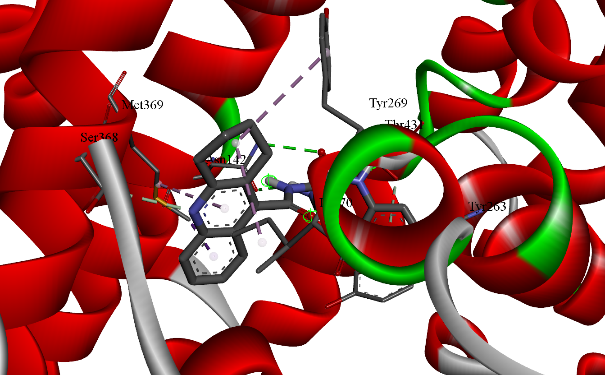

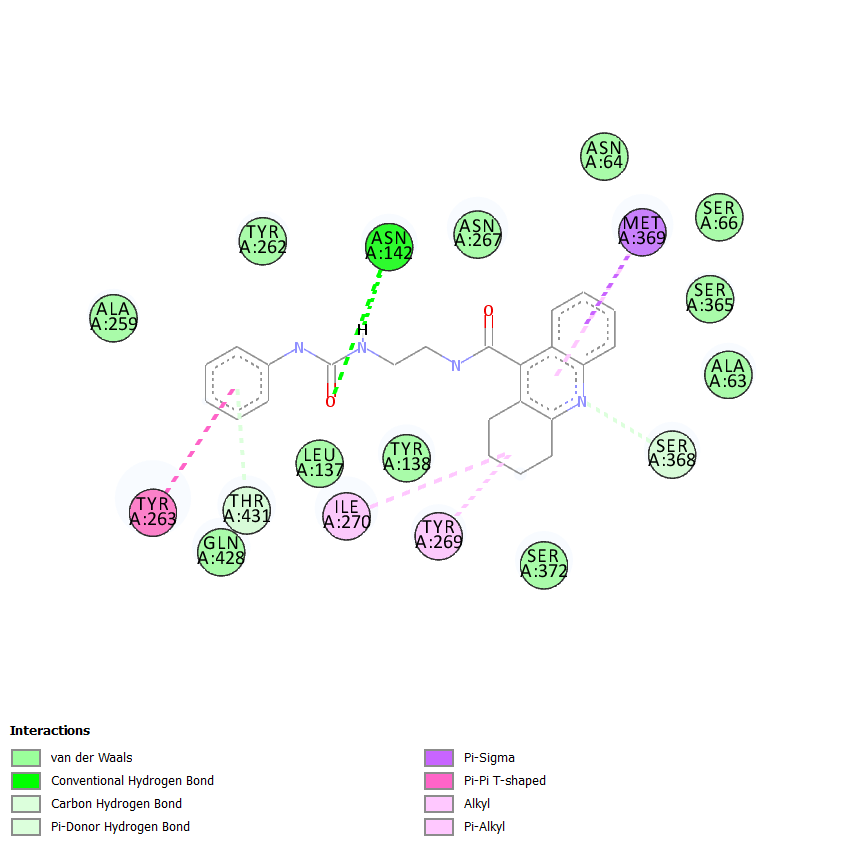


**Figure S8: Comp7a**

***Figures (S9-S13) of GLUT2 (PDB ID: 4pyp) for Comp4d, Comp4c, Comp4b, Comp4a and Comp7a, respectively.***


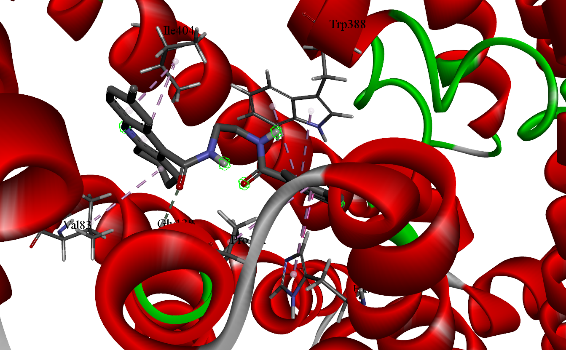

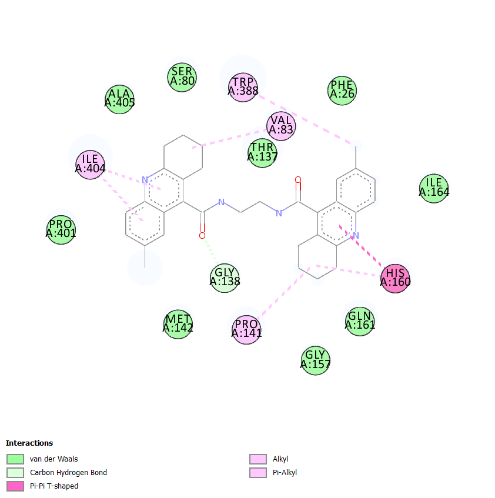


**Figure S9: Comp 4d**


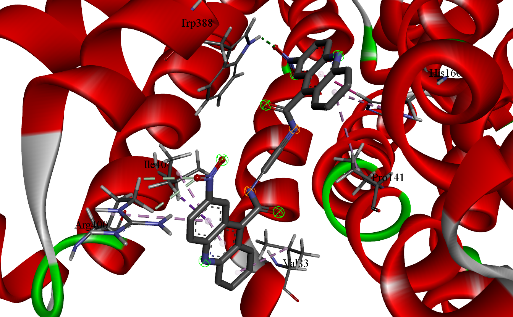

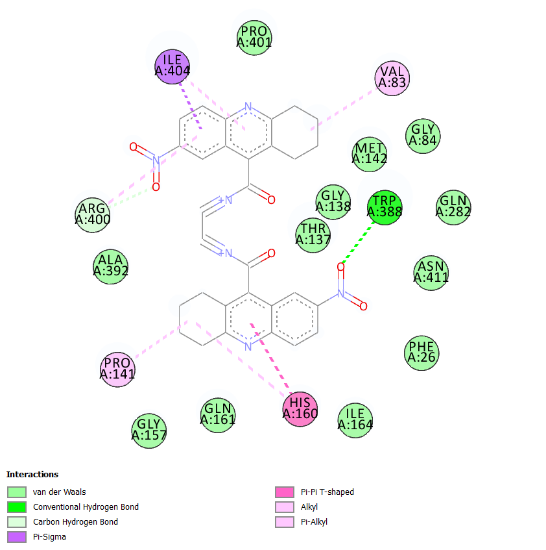


**Figure S10: Comp 4c**


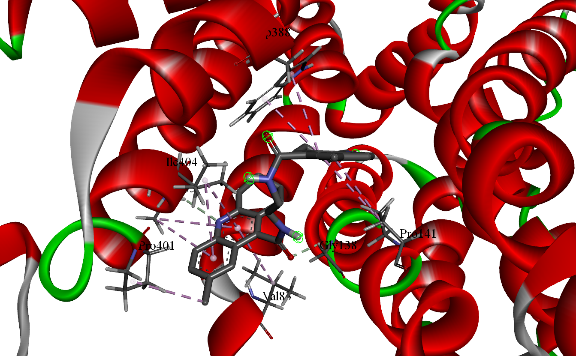

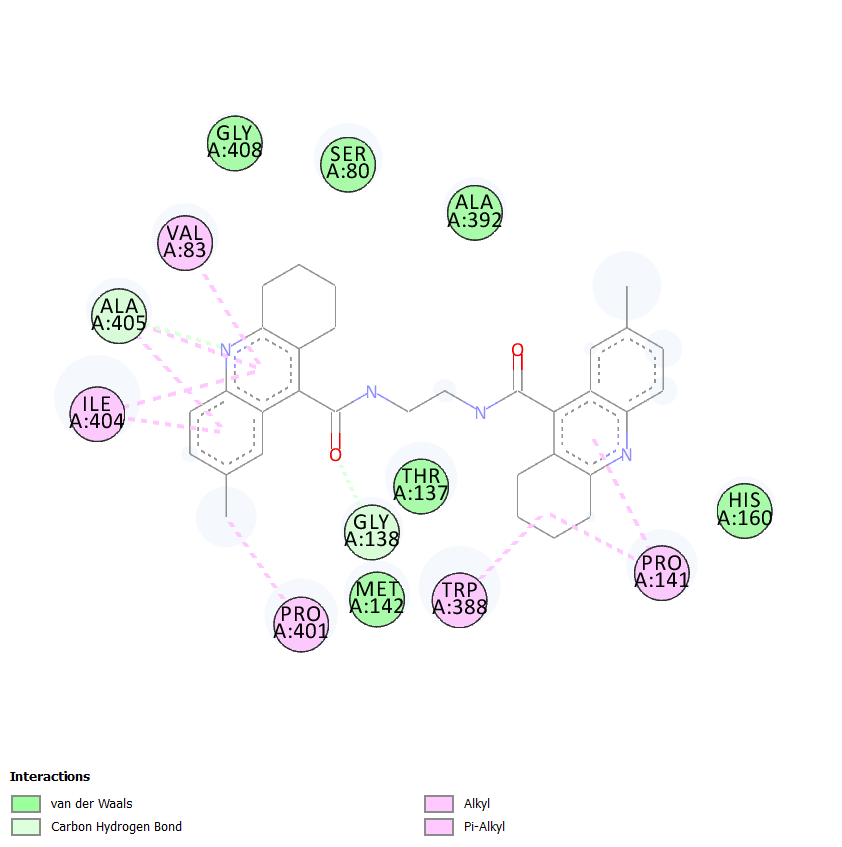


Figure S11: Comp4b


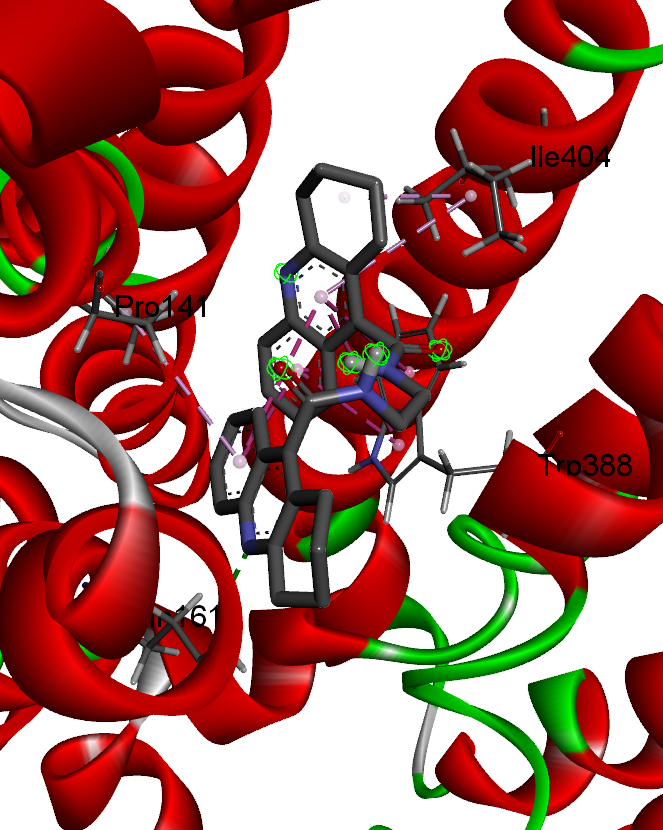

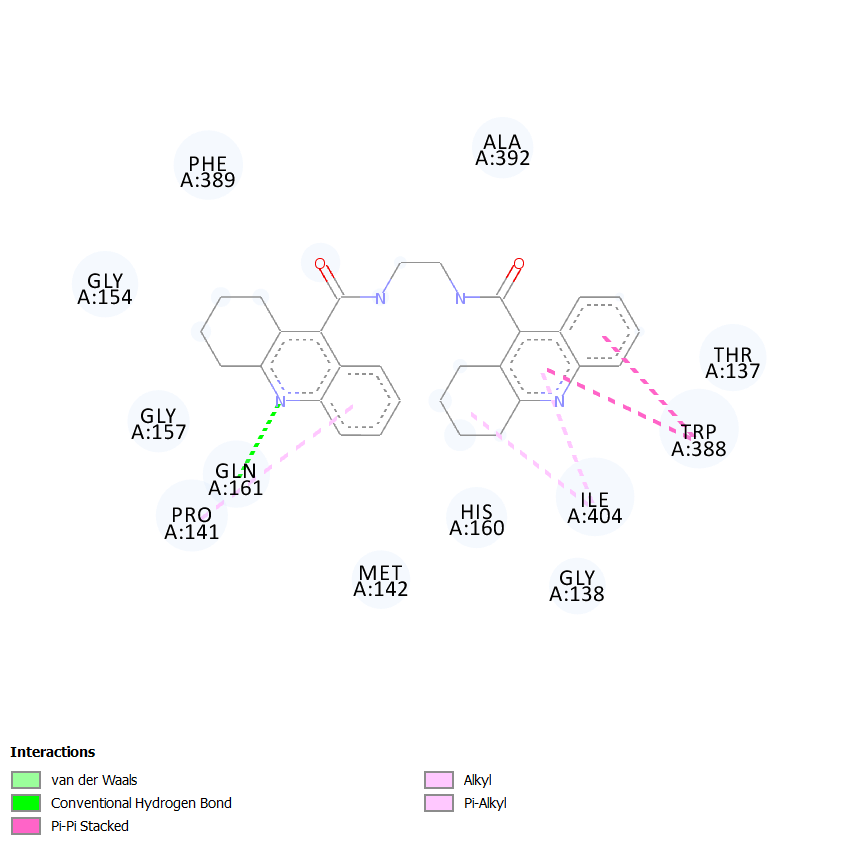


Figure S12: Comp4a


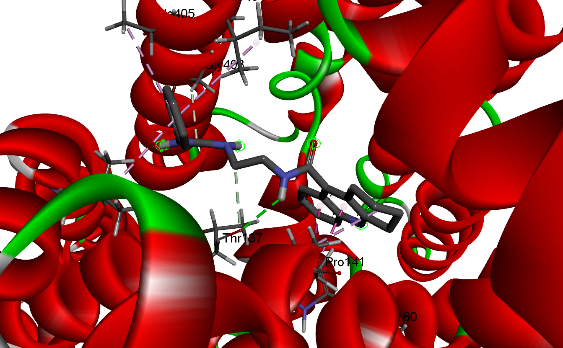

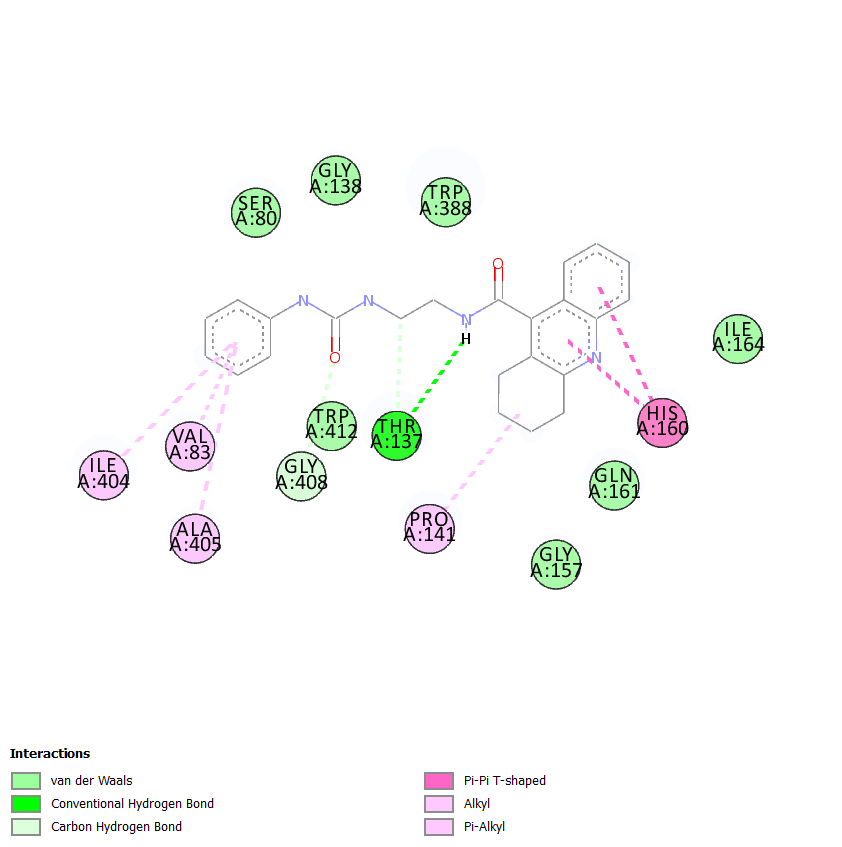


Figure S13: Comp 7a

***Figures (S14-S16) of Gliclazide (reference ligand) with PDB(ID): 4a5s, 3d4h, 4pyp respectively.***


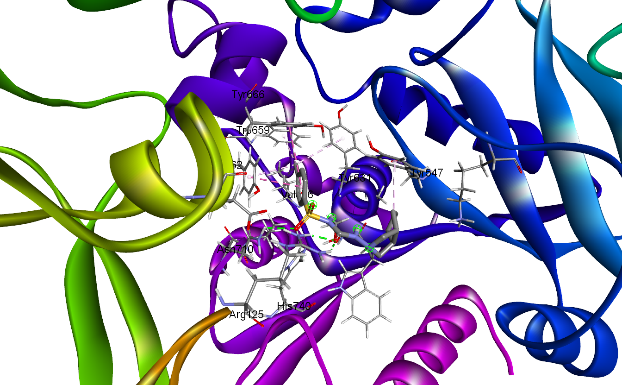

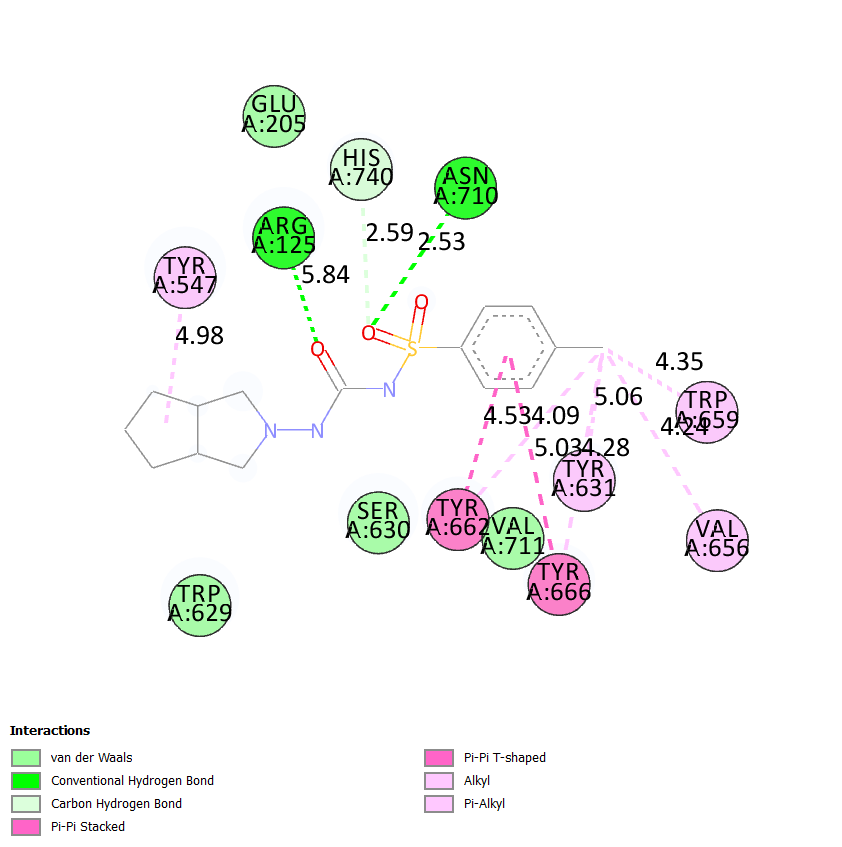


Figure S14; Gliclazide with 4a5s


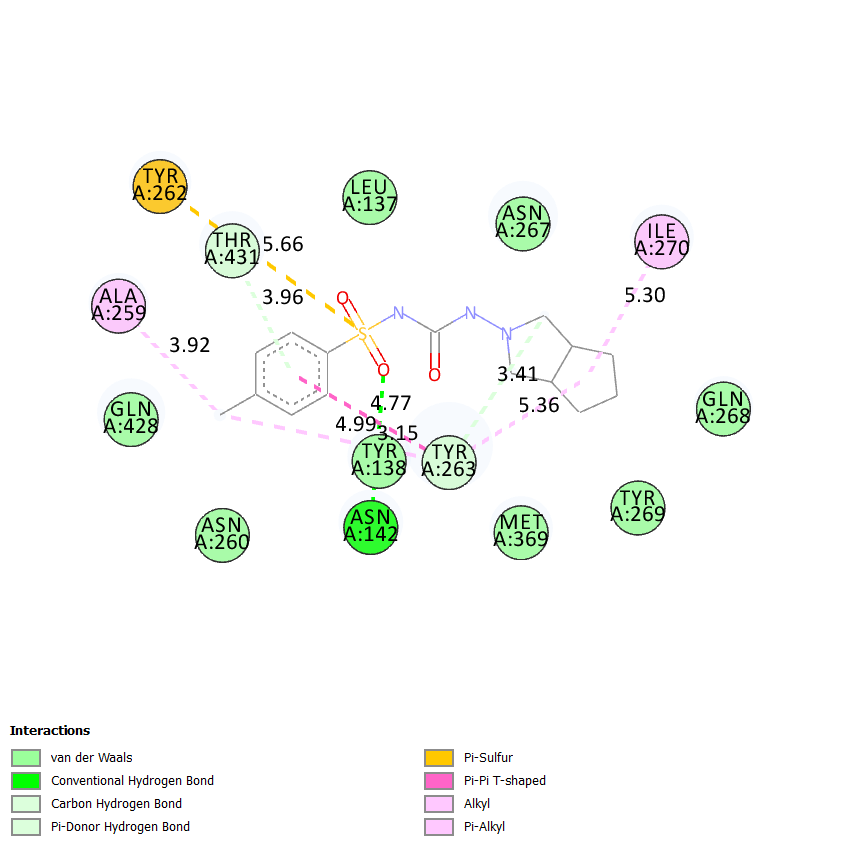

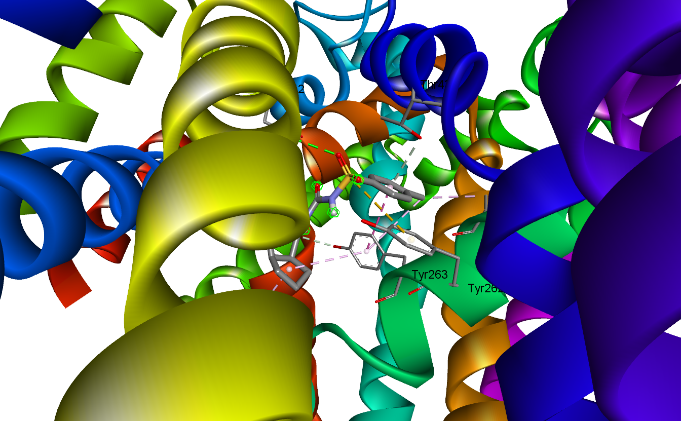

Figure S15: Gliclazide with 3d4h


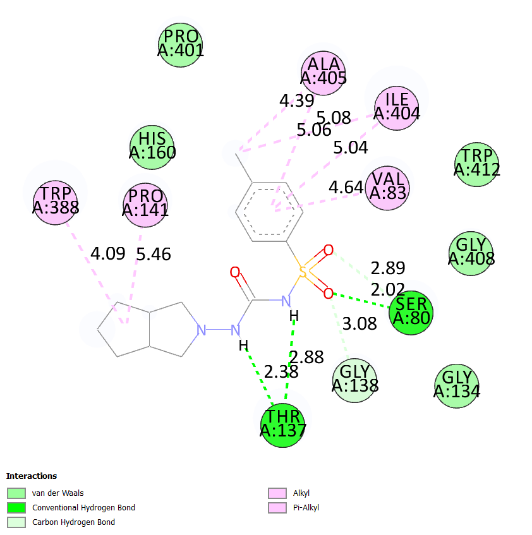

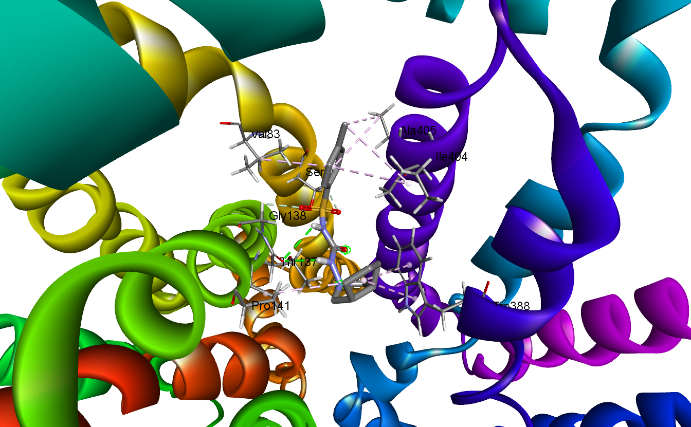

Figure S16: Gliclazide with 4pyp
